# Physical activity and sitting over 9 years of the Women’s Health Initiative Strong & Healthy (*WHISH*) randomized physical activity intervention trial in older women

**DOI:** 10.1093/gerona/glag189

**Published:** 2026-07-31

**Authors:** Marcia L Stefanick, Sally F Mackey, Joseph C Larson, Lesley F Tinker, Valerie McGuire, Corey M Rovzar, Marily Oppezzo, Michael J LaMonte, Charles Kooperberg, Andrea Z LaCroix, Abby C King

**Affiliations:** Department of Medicine (Stanford Prevention Research Center), School of Medicine, Stanford University, Stanford, California, United States; Department of Obstetrics and Gynecology, School of Medicine, Stanford University, Stanford, California, United States; Department of Medicine (Stanford Prevention Research Center), School of Medicine, Stanford University, Stanford, California, United States; Division of Public Health Sciences, Fred Hutchinson Cancer Center, Seattle, Washington, United States; Division of Public Health Sciences, Fred Hutchinson Cancer Center, Seattle, Washington, United States; Department of Medicine (Stanford Prevention Research Center), School of Medicine, Stanford University, Stanford, California, United States; Department of Medicine (Stanford Prevention Research Center), School of Medicine, Stanford University, Stanford, California, United States; Department of Medicine (Stanford Prevention Research Center), School of Medicine, Stanford University, Stanford, California, United States; Department of Epidemiology, State University of New York at Buffalo, Buffalo, New York, United States; Division of Public Health Sciences, Fred Hutchinson Cancer Center, Seattle, Washington, United States; Herbert Wertheim School of Public Health and Human Longevity Science, University of California, San Diego, La Jolla, California, United States; Department of Medicine (Stanford Prevention Research Center), School of Medicine, Stanford University, Stanford, California, United States; Department of Epidemiology and Population Health, School of Medicine, Stanford University, Stanford, California, United States; (Medical Sciences Section)

**Keywords:** Walking, Muscle strengthening, Sedentary behavior, Aging, Lifestyle change

## Abstract

**Background:**

The Women’s Health Initiative (WHI) Strong & Healthy (*WHISH*) randomized trial implemented a 9-year remotely delivered “light touch” multicomponent physical activity intervention (PA-I) aimed at increasing physical activity (PA) and reducing sitting time in a large U.S. cohort of older women.

**Methods:**

Using a randomized consent design, 49 331 women, aged 66–99 years, were assigned to Intervention (*N* = 24 657), 95.9% (*N* = 23 653) of whom provided passive consent to receive the *WHISH* PA-I, or “usual activity” Comparison (*N* = 24 674). Differences between intention-to-treat arms in self-reported walking, sitting and other PA, averaged over the course of the trial, were derived from a linear regression model with log-transformed outcomes as a function of arm, adjusted for participant selection strata.

**Results:**

Pre-randomization mean age (79.7 years), total recreational PA (11.6 MET-hours/week), RAND-36 physical function score, and other characteristics did not differ between trial arms. Over a median 8.7 years, Intervention participants reported higher average walking (3.2%), aerobic (3.4%), and total (3.7%) exercise, as MET-hours/week, and strength training sessions per week (4.4%), and fewer hours/week of sitting (1.5%) than Comparison (*p* < .001 for all). Intervention versus Comparison differences were greater for aerobic exercise (6.92%) and sitting (−2.48%) in the youngest age group (66 − 76) compared with other age groups (*p* <.001 and .02 for interaction , respectively).

**Conclusion:**

A 9-year “light touch” remotely-delivered PA intervention modestly attenuated age-related PA decreases and sitting time increases in older women. Building on the pragmatic *WHISH* PA intervention to augment behavioral changes, especially in adults aged over 80 years, is an important direction for population health.

## Introduction

Nationally recommended levels of physical activity (PA) and exercise^1^^,^^2^ are positively associated with better health and well-being in older adults.^3–11^ Despite strong associations of active aging, involving regular physical activity and avoiding prolonged sitting, with reduced risk of premature mortality and morbidity, physical inactivity and sedentary time remain high among older adults worldwide.^11^

Structured exercise programs tailored to individual needs and capacities of older adultsare effective strategies to enhance functional independence and increase PA within 6- to 12-months in community-dwelling older adults.^1–12^ However, few randomized controlled trials (RCTs) have followed older adults for more than two years or demonstrated maintenance of improved PA over time,^11^^,^^12^ defined as six months of sustained regular PA.^13^ Therefore, no intervention has been shown to prevent or even attenuate the well-established age-related decline in physical activity.

Evidence that increasing PA and reducing sedentary behavior can promote healthy aging at a population level is even more limited, largely because it is challenging to bring about even short-term behavior change at a community or broader population level. Most physical activity intervention studies attract only individuals who believe they are ready to commit to structured exercise sessions with specified PA goals, whether supervised at scheduled times or encouraged through remote strategies, or occurring at well-equipped (climate-controlled) facilities or in home- and neighborhood settings, with or without social support.^14–17^ Most physical activity RCTs also exclude individuals who are already physically active, despite well-known challenges and barriers to maintaining PA levels over time. Considering the high proportion of older adults who could possibly benefit from increasing current aerobic and multi-component PA levels and decreasing sedentary behavior by any amount, developing low-cost, scalable interventions that attenuate the age-related decline in PA has high public health value.

Motivated by a call from leaders of the National Heart Lung and Blood Institute in 2011–2013 to conduct pragmatic, low-cost trials, including exercise intervention trials, that would be more generalizable to the U.S. population,^18–20^ the Women’s Health Initiative (WHI) Strong & Healthy (*WHISH*) RCT deployed a randomized consent design to assign women within the large, well-characterized cohort of the WHI Extension Study,^21^ to a centralized pragmatic physical activity intervention (PA-I) or “usual activity” Comparison, with major cardiovascular disease (CVD) events as the primary health outcome.^22^^,^^23^

This paper describes the design and implementation of the “light touch,” ie delivered remotely with no individualized customization, *WHISH* Physical Activity Intervention (PA-I) and key differences in physical activity patterns between women aged 66–99 (mean 79.7) years, who were randomly assigned to the *WHISH* Intervention versus Comparison arms over a median of 8.7 years. The *WHISH* PA-I was designed to motivate a large number of older women residing across the United States to move more and sit less and become familiar with current national multi-component physical activity recommendations.^1^^,^^2^ The paper focuses on key differences between the intention-to-treat *WHISH* Intervention and Comparison arms with respect to self-reported walking, aerobic, and total exercise, measured as MET-hours per week, muscle strengthening sessions per week, and sitting hours per day.

## Methods

The *WHISH* trial protocol (Appendix A in the Supplementary Materials) was approved by the Institutional Review Boards of the WHI Clinical Coordinating Center (CCC) at the Fred Hutchinson Cancer Center and at Stanford University, and by the NHLBI-appointed Data Safety and Monitoring Board. The authors attest to the accuracy of the data analysis and fidelity of the intervention protocol.

### Study population

As previously described,^22^^,^^23^  *WHISH* trial participants were among 161 808 women who provided written consent, at ages 50–79 years (1993–1998) at 40 clinical centers across the United States, to be randomized to active versus control treatment arms within the WHI Clinical Trial (CT) or to enroll in the WHI Observational Study (OS) through 2005.^24^ In 2005, 115 407 (77% of eligible) CT/OS participants consented to continued follow-up through 2010, at which time 93 567 (87% of eligible) women consented to ongoing follow-up in the WHI-Extension Study 2 (WHI-ES2; 21). The WHI-ES2 consent enabled collection of health outcomes and all data needed for the *WHISH* trial.

In 2015, 61 444 of WHI-ES2 participants were alive and active and assessed for eligibility for the *WHISH* trial. Exclusion criteria included known dementia, living in a nursing home, self-reported inability to walk, or indicating Spanish language preference, based on WHI data available at *WHISH* randomization in 2015. To be eligible for *WHISH*, participants also had to have cardiovascular outcomes available through either WHI-ES2 outcomes adjudication^21^ or linkage to the Centers for Medicare and Medicaid Services (CMS) database (see Consort Diagram, Appendix B). Using a randomized consent design,^25^ 49 331 eligible participants were randomly assigned, within this community-based trial, stratified by 2015 age tertiles (66–76, 77–82, 83–99 years), U.S. census regions (Northeast, Southeast, Midwest, and West), and WHI-ES2 outcomes data source (adjudicated Medical Records or Self-report; 21) to Intervention (*N* = 24 657) or “usual activity” Comparison (*N* = 24 674) arms in parallel groups.

The WHI Clinical Coordinating Center at the Fred Hutchinson Cancer Center, Seattle, WA mailed all participants randomized to the *WHISH* Intervention a study description, an initial “*WHISH*ful Actions” newsletter and the NIA “*Go4Life: Workout to Go*” pamphlet.^26^ The mailing included a letter informing Intervention participants that the Stanford University *WHISH* Team would be contacting them to provide more information unless they called the CCC to let them know that they preferred not receiving any other *WHISH* materials. Passive consent to receive the PA-I was thereby obtained from 23 653 (95.6%) of *WHISH* Intervention participants. Names and contact information of Intervention participants who “opted out” of receiving the *WHISH* PA-I were not provided to the Stanford team. The Stanford team tracked the PA-I participants closely to ensure that PA-I mailings and any other contact with the Stanford team were stopped if a participant requested discontinuation at any time or was learned to have died. Participants in the comparison group were not informed of their role in the *WHISH* trial.

### Data collection and physical functioning assessments

Sociodemographic and lifestyle data were collected by questionnaire at WHI baseline (1993–1998) and during the main WHI study and WHI-ES2 follow-up. History of prior clinical CVD and use of antihypertensive and anti-hyperlipidemic medications were based on data collected at WHI enrollment and through follow-up to randomization in *WHISH* (2015). All *WHISH* participants received annual WHI-ES2 mailings with medical history update forms and a form with the 10-item RAND-36 Physical Functioning (PF) scale,^27^ for which scores ranged from 0 to 100 (in 5-point increments) and were categorized as lower (< 65), moderate (65–89), and higher (90–100) PF.

### Primary physical activity assessment and outcomes

Total recreational PA (presented roughly as tertiles: < 3, 3–< 14, ≥ 14 metabolic equivalent hours per week; MET-hrs/wk) and walking (MET-hrs/wk), described in detail previously,^28^ were collected on a WHI-ES2 form a median of 3.1 years (QR: 2.8–3.3) before randomization into the *WHISH* trial. After randomization, *WHISH* participants in both groups received annual PA questionnaires from the WHI CCC, labeled as WHI forms to maintain blinding of randomized trial arms (Appendix C1; revised in 2020, Appendix C2) which ascertained walking,^28^ sitting (hours per day), and other PA behaviors based on a modified Community Healthy Activities Model Program for Seniors (CHAMPS) questionnaire,^29^^,^^30^ including aerobic and total PA (MET-hr/wk), muscle strengthening (sessions per week), and sitting (hours per week).

### The physical activity intervention and annual PA-I surveys

The *WHISH* PA-I was a remotely-delivered “light touch” intervention, ie with no individualized customization or in-person physical activity coaching, in keeping with the pragmatic trial design. It was informed by state-of-the science behavioral theories, including Social Cognitive Theory,^31^ the Transtheoretical Model,^32^ and Self-determination Theory.^33^ Intervention messages aimed to motivate participants to increase their regular PA by any amount and informed them of national physical activity recommendations for older adults, ie, to accumulate 150 minutes a week of moderate-intensity aerobic activity, if possible, or aim to achieve PA levels corresponding to their current physical function status.^1^^,^^2^

Multi-component physical activity was emphasized with strong encouragement to walk and reduce sedentary behavior, particularly (inactive) sitting, every day, and do both muscle-strengthening sessions, including upper body and lower body exercises, and balance and flexibility activities at least two days a week. The PA-I was delivered primarily through quarterly (seasonal) newsletters, usually accompanied by inserts that provided instructions on specific exercises that were recommended or reviewed and approved by a physical therapist with geriatric expertise. Other communication channels included additional print materials, monthly automated outbound telephone messages, monthly emails (if participants provided email addresses), and a *WHISH* website.

The first three “*WHISH*ful Actions” newsletters were mailed within a 2-month window. These mailings included the NIA *Go4Life® Exercise & Physical Activity: Your Everyday Guide* book,^34^  *WHISH*-branded pedometers and easy-to-use PA tracking calendars with instructions on how to use them. Subsequent newsletters with “seasonal” themes were mailed every 3–4 months. The themes and content summary of the 36 *WHISH*ful Actions newsletter packets mailed over the 9-year intervention appear in Appendix D. Mailings also provided *WHISH*-branded resistance bands with specific exercise recommendations, pedometer belts, sun visors, neckerchiefs designed to promote specific exercises, fanny packs, and “Stick-with-it” notepads of postable logs, designed to create intentional weekly action plans over the course of the intervention. Participants could request replacements of any items at any time.

Brief (< 1 minute) motivational phone messages were delivered monthly by an automated telephone system using a familiar human voice, “Adriana,” unless a participant requested to not get these calls (requested by fewer than 2%). PA-I participants who provided email addresses, ie, 25% at the outset of the intervention, increasing to 35% by the final 2–3 years, received monthly emails with informational messages and links to vetted sites, such as *Go4Life®*^35^ and Move Your Way.^36^ A *WHISH* website provided additional resources and exercises and a PA self-tracking tool. Participants could also access an algorithm-driven interactive voice response (IVR) system, designed specifically for *WHISH* PA-I participants to offer tailored advice and support from “Adriana” and provided personal goal-setting features. Participants could also reach staff through email, phone, and postal mail throughout the intervention.

Participants were encouraged to complete action plans regularly, do self-assessments of physical function (and do specific exercises to improve their scores), come up with 2 minutes of an activity to replace sitting time throughout the day, complete 24-hour activity graphs (of their hours spent sleeping, resting or inactive sitting, active sitting and light activity, and moderate-to-vigorous activity), design exercises to be done in each room of their home, map out walking destinations of various distances from their home, complete Bingo-like cards of multi-component activities, lay out values that motivate them to move, and other projects designed to help them create personalized physical activity programs.

An array of motivational strategies to bring about PA change were encouraged through the above remote communication channels,^37^^,^^38^ including providing informational content (eg, news and research stories), emphasizing affective (thoughts and feelings) and social (friends, family, and caregiver activities) themes, and suggesting environmental cues and contexts (eg, coping with weather, strategically posting PA reminders, etc.). PA-I participants also served as “coping” role models by sharing personal stories, photos, and videos about overcoming barriers to PA (eg, recovery from injuries, illnesses, and personal losses) in “Life Happens” newsletter features and on the *WHISH* website.

The intervention was adapted in response to participant input, obtained primarily through annual PA-I surveys that were designed to solicit information on PA-I participant activity and exercise preferences, personal motivational drivers, barriers to physical activity and reactions to intervention materials and messages. The number (and percent) of respondents to each mailed or online survey, postcard, and other engagement materials were tracked closely, and reports were generated by age strata, race and ethnicity, and other factors to fine-tune the intervention, as warranted. Survey data were shared with PA-I participants in “All About You” newsletters.

Early feedback suggested that exercise materials were too challenging for some participants and not challenging enough for others. Therefore, starting in Fall 2018, alternative inserts were developed to present either less challenging or more challenging exercises than the “generic” insert. The 19 201 PA-I participants who were still receiving the *WHISH* PA-I at that time were triaged, based on self-reported PF and PA levels on recent WHI/*WHISH* forms, to receive the “Lower” (36.8%), “Middle” (31.9%), or “Higher” (31.3%) level PA-I inserts. Survey response rates were subsequently tracked by these “targeted” PF/PA insert groups.

Another example of adapting the intervention was a greater focus on balance after the majority of respondents to the 2021 Survey #6 indicated that they would like to make changes in their current physical activity. Improving balance was the most common specific change women in all three target groups checked, among five (“all that apply”) choices, followed by, in the same order for all target groups, strength, endurance, flexibility, and time spent sitting. From December 2022 through 2023, newsletters and targeted exercise inserts emphasized balance and how balance, strength, flexibility, and endurance related to mobility.

### Statistical analysis of physical activity by randomized arm

Demographics at *WHISH* randomization by trial arm, and for the PA-I subset of women, are presented with means and standard deviations for continuous characteristics and frequencies and percentages for categorical characteristics, with differences between arms assessed using *t*-tests and chi-square tests, respectively.

Trial PA and sitting per participant were calculated as the average of all yearly measurements over the course of the trial. Differences in participant average PA measures and sitting time are presented by trial arm with Intervention—Comparison percent differences and corresponding 95% confidence intervals from a linear regression model with the log-transformed trial average participant PA or sitting time outcome as a function of trial arm, adjusted for age strata, census region, and WHI-ES2 outcomes data source.^21^ The resulting absolute outcome difference between arms is calculated using the model-derived percent differences at the mean comparison outcome level.

To determine how differences in trial average PA and sitting outcomes by trial arm varied by age, subgroup models were fit to log-transformed outcomes as a function of trial arm, age group, and their interaction. Interaction *p*-values are from a separate linear regression model with the log-transformed outcome as a function of trial arm, linear trend over age groups, and their interaction. All linear models were adjusted for census region strata and WHI-ES2 sub-cohort.

Trial average PA and sitting outcomes were analyzed overall and by pre-randomization PF and PA level tertiles using least squares means at each calendar year, with means derived from a linear regression model with the outcome as a function of trial arm, adjusted for age strata, region strata, and WHI-ES2 outcomes sub-cohort.

Analyses were conducted in SAS for Windows version 9.4 (SAS Institute, Cary, NC) and *R* version 4.1.2 (R Foundation for Statistical Computing, Vienna, Austria).

## Results

There were no differences between Intervention (*N* = 24 657) and Comparison (*N* = 24 674) arms in participant characteristics at the time of randomization, including mean age (79.7 years) or race (86.8% White; 8.8% Black; 1.9% Asian/Pacific Islander; 0.2% American Indian/Alaska Native) or ethnicity (3.7% Hispanic/Latina). Nor did groups differ by pre-*WHISH* mean total recreational PA (11.5 MET-hr/wk) and walking (4.1 MET-hr/wk), mean RAND-36 PF score (69.9), most recent BMI (mean 28 kg/m^2^), current smoking (2.5%), history of CVD (13.9%), or use of antihypertensive (73.6%) or antihyperlipidemic (53.8%) medications (Table 1).

**Table 1:** Demographics at the time of WHISH randomization by intervention arm, all participants (*n* = 49 331).

	Intervention (*n* = 24 657)		Comparison (*n* = 24 674)	
Characteristic	*N*	%	*n*	%
**Age, mean (*SD*)**	79.8	(6.2)	79.7	(6.2)
66–76	8421	34.2	8457	34.3
77–82	8122	32.9	8098	32.8
83–99	8114	32.9	8119	32.9
**Ethnicity**				
Not Hispanic/Latina	23 700	96.1	23 729	96.2
Hispanic/Latina	915	3.7	900	3.6
Unknown/Not reported	42	0.2	45	0.2
**Race**				
American Indian/AK Native	48	0.2	56	0.2
Asian	452	1.8	423	1.7
Native Hawaiian/Other Pacific Islander	16	0.1	22	0.1
Black	2190	8.9	2161	8.8
White	21 398	86.8	21 439	86.9
More than one race	291	1.2	311	1.3
Unknown/not reported	262	1.1	262	1.1
**Region**				
Northeast	6010	24.4	6040	24.5
South	7063	28.6	7045	28.6
Midwest	5737	23.3	5751	23.3
West	5847	23.7	5838	23.7
**Education**				
≤High school/GED	4249	17.2	4126	16.7
School after high school	8730	35.4	8677	35.2
≥College degree	11 523	46.7	11 746	47.6
**Body mass index, kg/m^2^, mean (*SD*)**	28.0	(5.8)	28.0	(5.8)
< 25	8438	34.2	8418	34.1
25–< 30	8770	35.6	8754	35.5
≥ 30	7434	30.1	7490	30.4
**Physical function (0–100), mean (*SD*)**	69.8	(25.6)	70.0	(25.6)
< 65	8240	33.4	8142	33.0
65–89	8248	33.5	8230	33.4
≥ 90	8023	32.5	8143	33.0
**Smoking**				
Never	12 627	51.2	12 648	51.3
Past	11 295	45.8	11 270	45.7
Current	607	2.5	609	2.5
**Alcohol drinks/wk**				
< 1	14 736	59.8	14 632	59.3
1–< 7	6293	25.5	6404	26.0
≥ 7	3628	14.7	3638	14.7
**Physical Activity (MET-hr/wk), mean (*SD*)**	11.5	(12.3)	11.6	(12.3)
< 3	7117	28.9	7064	28.6
3–< 14	7758	31.5	7657	31.0
≥ 14	7698	31.2	7842	31.8
**Walking (MET-hr/wk),^a^ mean (*SD*)**	4.1	(5.8)	4.1	(5.8)
**CVD**	3469	14.1	3396	13.8
MI	1159	4.7	1087	4.4
CABG/PTCA	1683	6.8	1619	6.6
Stroke	1017	4.1	1042	4.2
HF	891	3.6	868	3.5
**Antihypertensive use**	18 148	73.6	18 182	73.7
**Antihyperlipidemic use**	13 238	53.7	13 318	54.0

Abbreviations: CABG, coronary artery bypass graft; CVD, cardiovascular disease; HF, heart failure; MET, metabolic equivalent of task; MI, myocardial infarction; PTCA, percutaneous transluminal coronary angioplasty; *SD*, standard deviation.

Missing data: education, *n* = 280; BMI, *n* = 27; physical function, *n* = 305; smoking, *n* = 275; physical activity, *n* = 4195; walking, *n* = 4195.

a Walking Pre-WHISH measurement is a median (IQR) of 3.1 (2.8–3.3) years prior to WHISH randomization.

Demographics are presented by age strata [66–76, 77–82, and 83–99 years] for all *WHISH* participants (eTable S1 in the Supplementary Materials). The 23 653 PA-I participants who consented to receiving the *WHISH* PA-I closely resembled the full Intervention group and showed similar patterns across the three age strata as the entire *WHISH* cohort (eTable S2 in the Supplementary Materials). Follow-up completeness was comparable between randomized groups based on the proportion of returned physical activity questionnaires (Expected 7.1; Intervention 5.4 [73.1%] versus Comparison 5.6 [75.8%] and the proportion of participants returning at least 5 questionnaires (76.8% vs 78.9%)).

Response rates to PA-I annual surveys (corresponding to year of PA-I data collection) were as follows: Survey 1 (2015), 49.1%; S2 (2016), 53.3%; S3 (2017), 63.0%; S4 (2018), 71.8%; S5 (2019), 70.8%; S6 (2021), 67.7%; S7 (2022–2023), 63.8%; and S8 (2024), 64.8%. Response rates were consistently and considerably higher in the youngest pre-randomization age tertile than older age tertiles and considerably lower in the oldest tertile (eTable S3 in Supplementary Materials).

Over a median of 8.7 years, Intervention participants reported 3.24% higher MET-hr/wk of walking compared to Comparison participants (Table 2), which equated to a mean difference of 0.11 (95% CI: 0.06, 0.17) MET-hr/wk, and 1.5% lower hours/week of sitting (*p* < .001 for both), which equated to a mean difference of −0.76 (95% CI: −1.13, −0.39) hours/week. The Intervention group also reported modestly higher aerobic exercise (3.4%) and total exercise MET-hr/wk (3.7%), and higher muscle strengthening exercise sessions per week (4.4%) (*p* < .001 for all).

**Table 2:** Self-reported physical activity by intervention arm.

	Intervention (*n* = 24 657)		Comparison (*n* = 24 674)		Intervention vs comparison		
Trial average physical activity	Mean^a^	*SD*	Mean	*SD*	% difference (95% CI)^b^	Difference at comparison mean (95% CI)^c^	*p*-Value^b^
**Walking, MET-hr/wk**	3.60	4.58	3.40	4.37	3.24 (1.63, 4.87)	0.11 (0.06, 0.17)	< .001
**Sitting, hr/wk**	51.46	19.35	52.27	19.60	−1.46 (−2.17, −0.74)	−0.76 (−1.13, −0.39)	< .001
***CHAMPS physical activity***							
Total exercise, MET-hr/wk	16.37	15.70	15.52	14.99	3.71 (1.78, 5.67)	0.58 (0.28, 0.88)	< .001
Aerobic exercise, MET-hr/wk	4.05	6.17	3.86	5.98	3.40 (1.58, 5.26)	0.13 (0.06, 0.20)	< .001
Strength training, sessions/wk	0.97	1.34	0.86	1.24	4.35 (3.30, 5.41)	0.04 (0.03, 0.05)	< .001

Abbreviations: CHAMPS, Community Healthy Activities Model Program for Seniors; CI, confidence interval; MET, metabolic equivalent of task; *SD*, standard deviation.

a Mean and standard deviations by arms derived from average participant physical activity/sitting over the course of the trial, weighted on follow-up time.

b Intervention–comparison percentage differences, corresponding 95% confidence intervals, and *p*-values are from a linear regression model with the log-transformed trial average outcome as a function of intervention arm, stratified by age strata, region strata, and WHI-ES2 sub-cohort.

c Differences at comparison mean values are the difference between arms applying the model-derived percent difference at the mean comparison group physical activity level.

**Figure 1:**
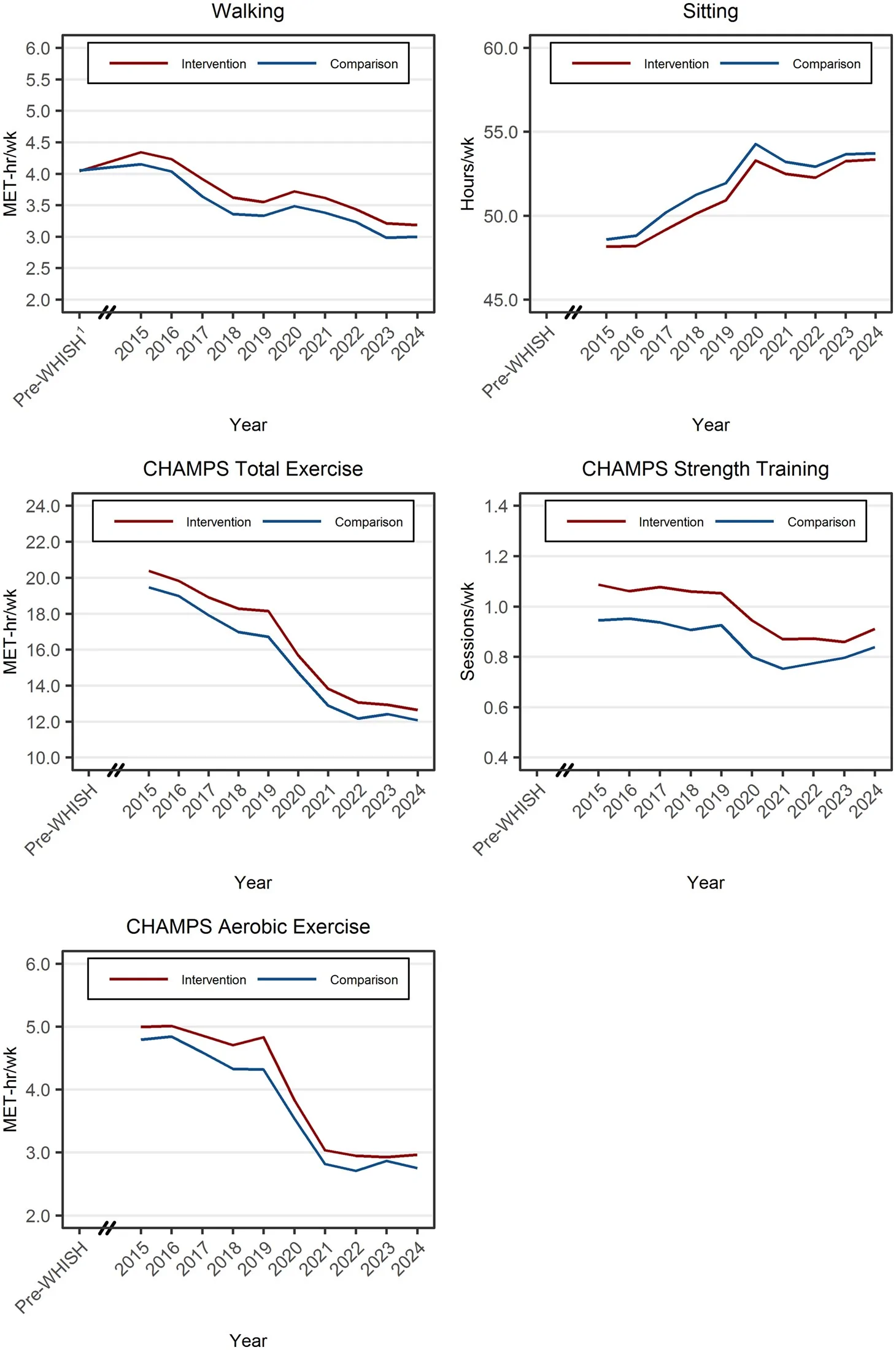
Self-reported physical activity outcomes by intervention arm. ^1^Walking Pre-*WHISH* measurement is a median (IQR) of 3.1 (2.8–3.3) years prior to *WHISH* randomization.

As seen in Figure 1, walking was higher in the Intervention versus Comparison group by the first post-randomization assessment, a median of 3.1 (IQR: 2.8–3.3) years after the pre-*WHISH* assessment, at which time there were no differences between trial arms. PA declined and sitting time increased steadily in both groups over the median 8.7 years after randomization, with noticeable changes in most measures coinciding with the 2020–2021 COVID-19 pandemic. In fact, walking increased in 2020 in both groups, then resumed its steady decline, while sitting time increased more steeply in 2020, followed by a decrease in both groups, and subsequent resumption of steadily increasing sitting time in the final two years. However, Intervention participants reported modestly higher MET-hr/wk of walking, aerobic and total PA and strength training sessions/week and fewer hours/week of sitting than Comparison across the full length of the trial, including during the 2020–21 COVID-19 era (Figure 1).

**Figure 2:**
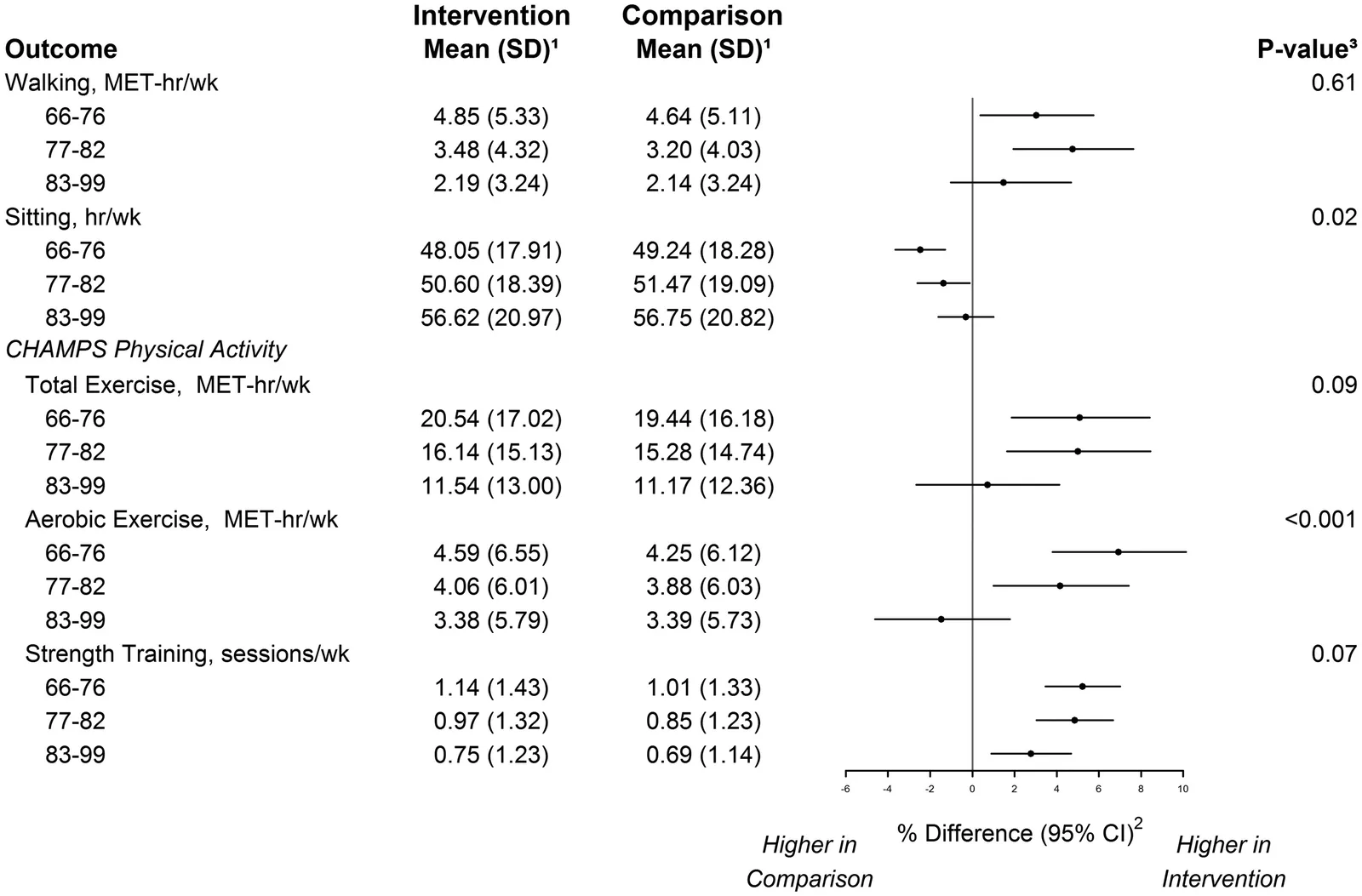
Physical activity by intervention arm by age at *WHISH* enrollment subgroups. ^1^Mean and standard deviations by arms derived from average participant physical activity/sitting over the course of the trial, weighted on follow-up time. ^2^Intervention–comparison percentage differences and corresponding 95% confidence intervals are from a linear regression model with the log-transformed trial average outcome as a function of intervention arm, age group and their interaction, stratified by age strata, region strata, and WHI-ES2 sub-cohort. ^3^Interaction *p*-Values from a separate model with the log-transformed trial average outcome as a function of intervention arm, linear trend over age group and their interaction.

There was a significant interaction between the linear trend across age at randomization group and the effectiveness of the intervention in reducing average aerobic exercise and sitting time (Figure 2). In the youngest age group (66–76 years), the Intervention group reported 6.9% more aerobic exercise than the Comparison group, as opposed to the reported I-C differences of 4.2% and −1.5% in the middle (77–82) and oldest (≥ 83) age groups, respectively (*p* <.001 for interaction). Also in the youngest age group, the Intervention group reported 2.5% less sitting time than the Comparison group, as opposed to the reported I-C differences of 1.4% and 0.3% less sitting time for the middle and oldest age groups, respectively (*p* .02 for interaction).

**Figure 3:**
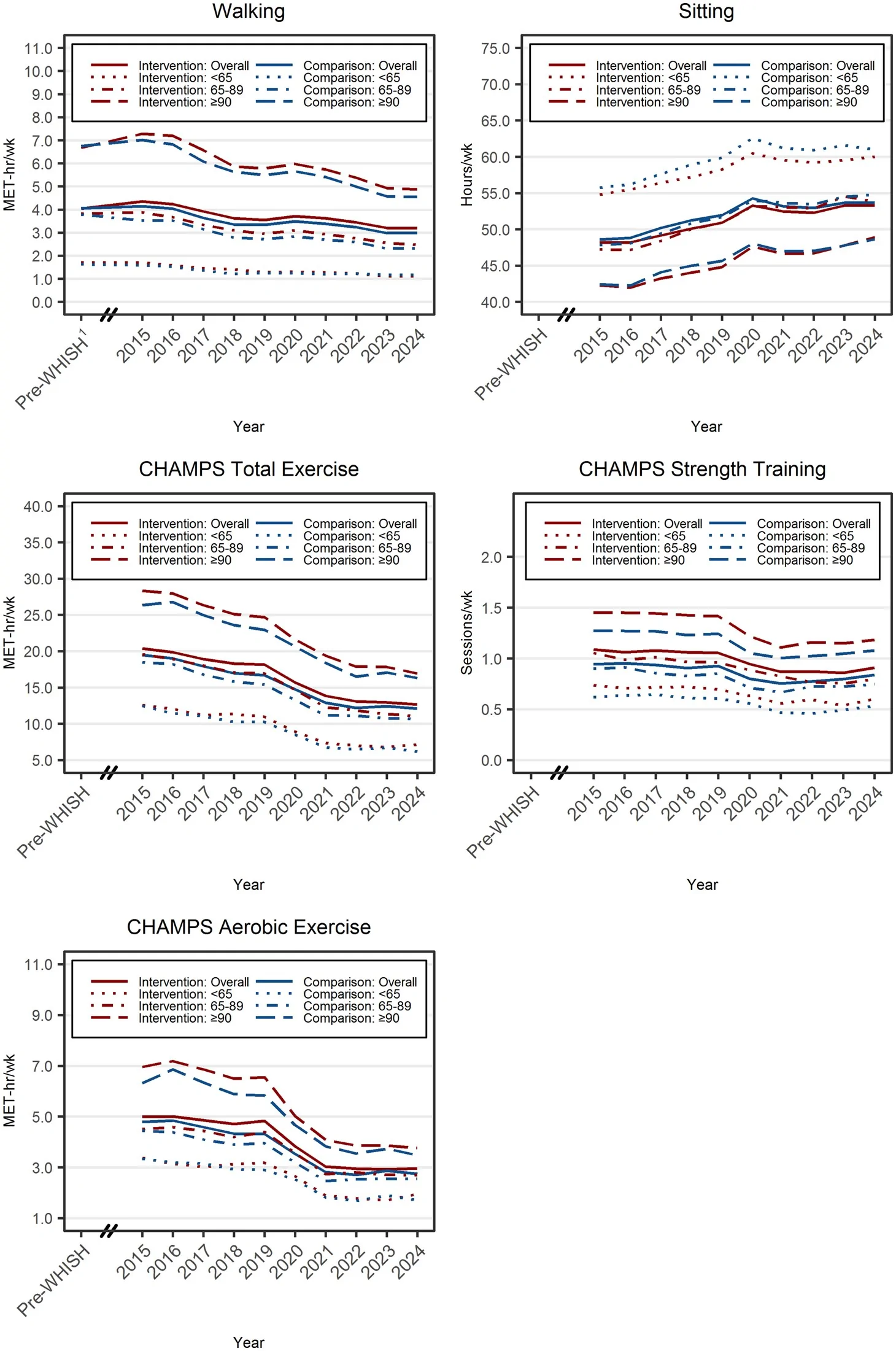
Self-reported physical activity outcomes by intervention arm and physical function (0–100) at *WHISH* enrollment. ^1^Walking Pre-*WHISH* measurement is a median (IQR) of 3.1 (2.8–3.3) years prior to *WHISH* randomization.

As seen in Figure 3, the intervention appeared to be effective in increasing walking and all PA measures in women with higher and middle pre-*WHISH* PF scores (PF ≥ 90 and PF = 65–89, respectively), but not in participants with lower pre-randomization PF scores. The intervention also appeared to be more effective in increasing walking and PA measures in women with higher pre-*WHISH* PA levels 3–< 14 and ≥ 14 MET-hr/wk, but not in participants who reported lower (< 3 MET-hr/wk) pre-randomization PA levels (eFigure S1 in the Supplementary Materials). Overall, the intervention appeared to increase the number of strength training sessions/week in all PF groups (Figure 3) and in all PA groups (eFigure S1 in the Supplementary Materials) over the course of the trial.

## Discussion

The Women’s Health Initiative (WHI) Strong & Healthy trial demonstrated that a remotely-delivered, “light touch,” multicomponent physical activity intervention can attenuate the age-related decline in self-reported physical activity (PA) and increase in sitting time over 9 years of follow-up in a large U.S. cohort of women, aged 66–99 years (mean 79.7 years). While all PA measures decreased and sitting increased as participants aged in both trial arms, women assigned to the Intervention arms reported statistically significant (*p* < .001 for all) higher MET-hours per week of walking (3.2%), aerobic (3.4%) and total (3.7%) exercise, as well as strength training sessions per week (4.4%), and fewer hours per week of sitting (−1.5%), than the Comparison arm over the course of the trial. Acknowledging that most *WHISH* participants were doing a mix of walking and light intensity PA, and relatively little moderate-to-vigorous activity, the difference in total exercise of 0.58 MET-hr/wk (95% CI: 0.28, 0.88) between trial arms is roughly equivalent to a difference of 14 minutes per week of walking at a slow pace of 2.5 METS. Clearly, the magnitude of PA improvements was dwarfed by the secular decline in PA measures and increase in sitting in the aging cohort (Figure 1); nonetheless, the dearth of long-term randomized controlled trial data on physical activity in geriatric populations makes these findings unique and of considerable public health value.

Identifying strategies to enhance the effectiveness of a remotely delivered PA intervention to achieve greater increases in physical activity and decreases in sedentary behavior than achieved by the *WHISH* PA intervention has considerable scientific merit, particularly for testing hypotheses regarding health benefits of improving PA and reducing sitting time. In keeping with the pragmatic nature of the *WHISH* trial, PA-I participants were not required to return PA logs or action plans; however, annual PA-I surveys collected a wealth of information which will be evaluated, including whether participants used any of the *WHISH* PA self-tracking tools. Many reported using the annual *WHISH* Activity calendars, which provided space to enter active minutes and steps, and check whether a participant did upper-body and lower-body muscle strengthening and/or balance and flexibility exercises each day of the week. On the other hand, most PA-I participants did not access the website; therefore, few tried the web PA self-tracking tool. Even fewer participants attempted to use the *WHISH* IVR system, and therefore only a small percent used its goal-setting feature.

The IVR system had been inspired by the success of an automated telephone-linked computer system which delivered physical activity counseling in the Community Health Advice by Telephone (CHAT) trial to motivate adults aged 55 years and older (mean 61.6) to increase PA above a target of 150 minutes per week within 6 months and maintain it for another 6 months.^39^ We had demonstrated the feasibility and acceptability of a *WHISH* IVR-prototype as a means to deliver tailored physical activity counseling, designed around the Go4Life® educational materials^26^^,^^34^^,^^35^ in a 10-week pilot study of 30 WHI-ES2 participants before initiating the *WHISH* trial.^40^ Unfortunately, scheduling regular IVR contacts for over 23, 000 women was not feasible within the budget constraints of the pragmatic trial, rendering that technology suboptimal for the *WHISH* PA-I. The system was, however, repurposed to deliver monthly outbound motivational messages, which most participants appreciated, with only 2% requesting that the calls be stopped, but the mailings continued.

PA-I surveys also asked participants about their use of each of the intervention materials. A 2019 analysis of 18 080 *WHISH* PA-I participants found that, except the IVR system, engagement with each of the *WHISH* PA-I delivery channels, eg, targeted inserts, email (opened), email (clicked links), website (logging in), and website (tracking), was associated with more hours/week of physical activity.^41^ Consistently across channels, PA-I subgroups with higher engagement included younger age, a subgroup which also returned PA-I surveys at a much higher rate (eTable S3 in the Supplementary Materials), and higher levels of PA and physical function.^41^

PA-I surveys also asked about motivational drivers of physical activity, as did a postcard that was mailed to 19 598 PA-I participants with a simple prompt, “Think about the last time you didn’t feel like being active. How did you get yourself to move or get up?” Among the 4108 (20.9%) participants who returned the postcard, women who endorsed more strategies had more hours of PA and walking.^38^ In addition, strategic categories that correlated with more PA included focusing on the benefits and utilizing the surrounding environment to help motivate movement.^38^

Further systematic exploration of PA-I participants who particularly benefited from this resource-efficient light-touch intervention versus those who did not is warranted in each age group, given the heterogeneous composition of the older population. For example, the noticeable increase in walking in 2020, coinciding with the COVID-19 pandemic, and the steep increase in sitting time in women in both trial arms were particularly observable in participants who had reported the highest Pre-*WHISH* PF scores (Figure 3) and PA levels (eFigure S1 in the Supplementary Materials). Meanwhile, the *WHISH* PA-I seemed to have had virtually no effect on women in the lowest pre-*WHISH* PF and PA tertiles (Figure 3 and eFigure S1 in the Supplementary Materials), except for the modest increase in reported muscle strengthening sessions/week, which was also increased in the Intervention versus Comparison arms in all three age strata (Figure 2).

The design and conduct of the *WHISH* pragmatic trial differ from most previous PA trials first and foremost by not having actively recruited individuals who were interested, ready and willing to be randomized to a PA intervention or excluding individuals who were already meeting aerobic physical activity goals. The randomized consent design^25^ deployed in the *WHISH* trial aimed to simulate real-world implementation of a program that could be disseminated and implemented at a national level, ie, be generalizable to older women. Women assigned to the Comparison group, as well as Intervention women who opted out of receiving the PA-I materials, had access to the Go4LifeR® materials^26^^,^^34^ and national websites^35^^,^^36^ that PA-I participants were encouraged to use and may have been fully aware of national PA recommendations. Nonetheless, the “light touch” *WHISH* PA intervention resulted in significant, albeit quite modest, self-reported behavior changes in all PA measures and sitting time, particularly in women in younger age strata (66–76 and 77–82). Further investigation of scalable, resource-efficient methods for improving PA in the oldest old, ie, ≥ 85 years of age, seems particularly warranted as this group is one of the fastest growing segments of the U.S. population.^42^ Additionally, and speculatively, targeting adults aged 65–79 years, or even younger, might help them maintain PA behaviors into their oldest old years. While the “oldest old” are overrepresented in the *WHISH* subgroups with low initial PF and PA levels, the younger women with low PF and low PA levels are clearly another group to target for investigation of effective, cost-efficient strategies to increase PA within the community setting.

Strengths of the *WHISH* trial include the large number of women aged 66–99 years and the long-term “light touch” intervention, which resembles many booster strategies to promote maintenance of physical activity,^14^ and the many motivational principles that were promoted. As WHI participants, *WHISH* participants have been followed since 1993–1998 during which their physical activity, diet, and other lifestyle habits, as well as health outcomes, have been well documented. They currently reside in every state in the United States and are reasonably representative of the racial and ethnic composition of U.S. women of this age.^43^ They will also continue to be followed for several more years for physical activity and health outcomes.

A major weakness is that physical activity and sedentary behaviors are based on self-report, and the Intervention participants were not blinded to their randomization assignment, as is true for almost all lifestyle intervention studies. An intervention weakness is that we were not able to tailor it to the individual needs of the participants. Initially, only 25% of PA-I participants even used email, and although this increased to about a third as the trial continued, largely due to deaths of the oldest participants, the *WHISH* PA-I cohort was not, in general, tech savvy. Future cohorts of older adults already familiar with email, text, Zoom, and other communication channels, including IVR systems, will likely engage more readily with these channels, including IVR systems, making it easier to tailor remotely-delivered PA interventions, even in a pragmatic trial design.

Whether the modest changes in PA and sitting translate into clinically meaningful health benefits is currently being investigated and will be the focus of additional analyses. We will also analyze the vast amount of data collected from the PA-I surveys and explore factors associated with greater PA changes with aging. Considering the diversity of the *WHISH* cohort, we hope to gain insights into how to customize remotely-delivered PA interventions to different segments of this growing and diverse population for whom maintaining the ability to move is critical to enhancing the healthspan of older adults and preserving their independence. Building on the pragmatic *WHISH* physical activity intervention to augment behavioral changes, especially in the oldest age groups, is an important direction for population health.

## Supplementary Material

glag189_Supplementary_Data

## Data Availability

Deidentified participant data can be accessed by permission of the WHI, per The WHI Data Sharing Statement. Statistical code, data dictionary, and other materials can be requested by contacting the WHI Help Desk (helpdesk@whi.org).
