# Supplementary material for: Physical activity and sitting over 9 years of the Women’s Health Initiative Strong & Healthy (WHISH) randomized physical activity intervention trial in older women
Source: J Gerontol A Biol Sci Med Sci. 2026 Jul 31;81(9):glag189. doi: 10.1093/gerona/glag189 (PMC13500112; doi:10.1093/gerona/glag189)
Supplement: Supplementary file 1 [file glag189_supplementary_data.pdf]

Physical Activity and Sitting over 9 years of the Women's Health Initiative Strong & Healthy (WHISH) Randomized Physical Activity Intervention Trial in Older Women

SUPPLEMENTARY TABLES, FIGURES, AND APPENDICES

eTable 1. Demographics at the Time of WHISH Randomization Overall and by Age Group, All Participants (n=49,331)

eTable 2. Demographics at the Time of WHISH Randomization Overall and by Age Group, Participants who Started Intervention (n=23,653)

eTable 3. WHISH PA-I Survey Responders, Overall and by Age at Randomization

eFigure 1. Self-reported Physical Activity Outcomes by Intervention Arm and Physical Activity (MET-hr/wk) at WHISH Enrollment

Alt-Text: A composite figure containing five line graphs tracking self-reported physical activity physical activity and sitting trends from 2015 to 2024 for the Intervention (red line) and Comparison (blue line) groups, with separate line types for all participants (solid lines) as well as pre-trial physical activity (MET-hr/ wk) levels of <3 (dotted lines), 3-<14 (alternating dash/dot lines), and ≥14 (dashed lines). The panels monitor Walking, Sitting hours, CHAMPS Total Exercise, CHAMPS Strength Training, and CHAMPS Aerobic Exercise. Across all panels, exercise metrics generally decline over time while sitting hours increase. The Intervention group consistently maintains higher average exercise levels and lower average sitting hours than the Comparison group throughout the study timeframe. Participants with higher physical activity prior to trial start consistently report higher exercise and less sitting.

Appendix A. WHISH Protocol

Appendix B. WHISH Trial Design CONSORT

Alt-Text: a CONSORT diagram for the WHISH trial. 61,444 potential participants were assessed for eligibility, of which 49,331 were randomized. Of the 24,657 participants allocated to the intervention all 24,657 contributed follow-up, and 21,860 were analyzed for physical activity and/or sitting outcomes. Of the 24,674 participants allocated to the comparison group, all 24,674 contributed follow-up, and 22,502 were analyzed for physical activity and/or sitting outcomes.

Appendix C1. Form 521

Appendix C2. Form 521 v2

Appendix D. Table of WHISHful Actions Newsletters

Appendix E. CONSORT Checklist

eTable 1. Demographics at the time of WHISH Randomization Overall and by Age Group, All Participants (n=49,331)

	All Participants (n=49331)		66 – 76 (n=16878)		77 – 82 (n=16220)		83 – 99 (n=16233)	
**Characteristic**	n	%	n	%	n	%	n	%
Age, mean (SD)	79.7	(6.2)	73.1	(2.2)	79.4	(1.7)	87.0	(3.2)
Ethnicity								
Not Hispanic / Latina	47429	96.1	15991	94.7	15640	96.4	15798	97.3
Hispanic / Latina	1815	3.7	847	5.0	556	3.4	412	2.5
Unknown / Not reported	87	0.2	40	0.2	24	0.1	23	0.1
Race								
American Indian / AK Native	104	0.2	55	0.3	28	0.2	21	0.1
Asian	875	1.8	339	2.0	263	1.6	273	1.7
Native Hawaiian / Other PI	38	0.1	21	0.1	11	0.1	6	0.0
Black	4351	8.8	1883	11.2	1441	8.9	1027	6.3
White	42837	86.8	14069	83.4	14105	87.0	14663	90.3
More than one race	602	1.2	257	1.5	208	1.3	137	0.8
Unknown / Not reported	524	1.1	254	1.5	164	1.0	106	0.7
Region								
Northeast	12050	24.4	3473	20.6	4250	26.2	4327	26.7
South	14108	28.6	5453	32.3	4620	28.5	4035	24.9
Midwest	11488	23.3	3765	22.3	3851	23.7	3872	23.9
West	11685	23.7	4187	24.8	3499	21.6	3999	24.6
Education								
High school / GED	8375	17.0	2208	13.1	2960	18.2	3207	19.8
School after high school	17407	35.3	5775	34.2	5770	35.6	5862	36.1
≥College degree	23269	47.2	8781	52.0	7394	45.6	7094	43.7
Body Mass Index, kg/m^2, mean (SD)	28.0	(5.8)	28.4	(6.3)	28.2	(5.8)	27.4	(5.2)
<25	16856	34.2	5766	34.2	5295	32.6	5795	35.7
25 - <30	17524	35.5	5538	32.8	5840	36.0	6146	37.9
≥30	14924	30.3	5562	33.0	5077	31.3	4285	26.4
Physical Function (0-100), mean (SD)	69.9	(25.6)	78.8	(22.1)	71.0	(24.4)	59.6	(26.5)
<65	16382	33.2	3373	20.0	5043	31.1	7966	49.1
65 - 89	16478	33.4	5354	31.7	5842	36.0	5282	32.5
≥90	16166	32.8	8101	48.0	5245	32.3	2820	17.4
Smoking								
Never	25275	51.2	8273	49.0	8130	50.1	8872	54.7
Past	22565	45.7	7922	46.9	7616	47.0	7027	43.3
Current	1216	2.5	603	3.6	385	2.4	228	1.4
Alcohol drinks/wk								
<1	29368	59.5	9973	59.1	9554	58.9	9841	60.6

1 - <7	12697	25.7	4506	26.7	4238	26.1	3953	24.4
≥7	7266	14.7	2399	14.2	2428	15.0	2439	15.0
Physical Activity (MET-hr/wk), mean (SD)	11.6	(12.3)	13.6	(13.50)	11.7	(12.2)	9.2	(10.6)
<3	14181	28.7	4036	23.9	4474	27.6	5671	34.9
3 - <14	15415	31.2	5192	30.8	5189	32.0	5034	31.0
≥14	15540	31.5	6303	37.3	5250	32.4	3987	24.6
Walking[1] (MET-hr/wk), mean (SD)	4.1	(5.8)	5.1	(6.5)	4.2	(5.7)	3.0	(4.7)
CVD	6865	13.9	1361	8.1	2187	13.5	3317	20.4
MI	2246	4.6	408	2.4	726	4.5	1112	6.9
CABG/PTCA	3302	6.7	661	3.9	1092	6.7	1549	9.5
Stroke	2059	4.2	405	2.4	648	4.0	1006	6.2
HF	1759	3.6	297	1.8	512	3.2	950	5.9
Antihypertensive Use	36330	73.6	11099	65.8	12040	74.2	13191	81.3
Antihyperlipidemic use	26556	53.8	8354	49.5	9057	55.8	9145	56.3

Note: Abbreviations: CABG, Coronary Artery Bypass Grafting; CVD; cardiovascular disease; HF, heart failure; MET, Metabolic Equivalent of Task; MI, myocardial infarction; PTCA, Percutaneous Transluminal Coronary Angioplasty; SD, standard deviation

Missing data: education, n=280; BMI, n=27; physical function, n=305; smoking, n=275; physical activity, n=4195; walking, n=4195

[1]Walking Pre-WHISH measurement is a median (IQR) of 3.1 (2.8-3.3) years prior to WHISH randomization.

eTable 2. Demographics at the time of WHISH Randomization Overall and by Age Group, Participants who Started Intervention (n=23,653)

	All Participants (n=23653)		66 – 76 (n=8243)		77 – 82 (n=7804)		83 – 99 (n=7606)	
**Characteristic**	**n**	**%**	**n**	**%**	**n**	**%**	**n**	**%**
Age, mean (SD)	79.6	(6.2)	73.1	(2.2)	79.4	(1.7)	87.0	(3.2)
Ethnicity								
Not Hispanic / Latina	22724	96.1	7800	94.6	7528	96.5	7396	97.2
Hispanic / Latina	887	3.8	422	5.1	264	3.4	201	2.6
Unknown / Not reported	42	0.2	21	0.3	12	0.2	9	0.1
Race								
American Indian / AK Native	47	0.2	26	0.3	9	0.1	12	0.2
Asian	439	1.9	172	2.1	129	1.7	138	1.8
Native Hawaiian / Other PI	14	0.1	7	0.1	5	0.1	2	0.0
Black	2140	9.0	931	11.3	713	9.1	496	6.5
White	20479	86.6	6877	83.4	6763	86.7	6839	89.9
More than one race	280	1.2	108	1.3	105	1.3	67	0.9
Unknown / Not reported	254	1.1	122	1.5	80	1.0	52	0.7
Region								
Northeast	5761	24.4	1697	20.6	2042	26.2	2022	26.6
South	6787	28.7	2672	32.4	2220	28.4	1895	24.9
Midwest	5484	23.2	1836	22.3	1857	23.8	1791	23.5
West	5621	23.8	2038	24.7	1685	21.6	1898	25.0
Education								
High school / GED	4029	17.0	1081	13.1	1417	18.2	1531	20.1
School after high school	8347	35.3	2814	34.1	2748	35.2	2785	36.6
≥College degree	11130	47.1	4293	52.1	3583	45.9	3254	42.8
Body Mass Index, kg/m^2, mean (SD)	28.0	(5.8)	28.3	(6.2)	28.2	(5.8)	27.5	(5.2)
<25	8063	34.1	2871	34.8	2521	32.3	2671	35.1
25 - <30	8386	35.5	2680	32.5	2806	36.0	2900	38.1
≥30	7189	30.4	2685	32.6	2473	31.7	2031	26.7
Physical Function (0-100), mean (SD)	70.1	(25.5)	78.8	(22.0)	70.9	(24.4)	59.9	(26.3)
<65	7802	33.0	1668	20.2	2427	31.1	3707	48.7
65 - 89	7940	33.6	2597	31.5	2843	36.4	2500	32.9
≥90	7771	32.9	3953	48.0	2499	32.0	1319	17.3
Smoking								
Never	12072	51.0	4014	48.7	3889	49.8	4169	54.8
Past	10873	46.0	3901	47.3	3689	47.3	3283	43.2
Current	586	2.5	296	3.6	185	2.4	105	1.4
Alcohol drinks/wk								
<1	14095	59.6	4872	59.1	4593	58.9	4630	60.9

1 - <7	6061	25.6	2188	26.5	2037	26.1	1836	24.1
≥7	3497	14.8	1183	14.4	1174	15.0	1140	15.0
Physical Activity (MET-hr/wk), mean (SD)	11.6	(12.4)	13.6	(13.5)	11.7	(12.3)	9.2	(10.6)
<3	6752	28.5	1983	24.1	2148	27.5	2621	34.5
3 - <14	7446	31.5	2538	30.8	2512	32.2	2396	31.5
≥14	7442	31.5	3088	37.5	2506	32.1	1848	24.3
Walking[1] (MET-hr/wk), mean (SD)	4.2	(5.8)	5.0	(6.4)	4.3	(5.8)	3.1	(4.7)
CVD	3290	13.9	660	8.0	1076	13.8	1554	20.4
MI	1088	4.6	210	2.5	380	4.9	498	6.5
CABG/PTCA	1602	6.8	329	4.0	550	7.0	723	9.5
Stroke	958	4.1	187	2.3	308	3.9	463	6.1
HF	843	3.6	143	1.7	252	3.2	448	5.9
Antihypertensive Use	17388	73.5	5401	65.5	5808	74.4	6179	81.2
Antihyperlipidemic use	12683	53.6	4061	49.3	4356	55.8	4266	56.1

Note: Note: Abbreviations: CABG, Coronary Artery Bypass Grafting; CVD, cardiovascular disease; HF, heart failure; MET, Metabolic Equivalent of Task; MI, myocardial infarction; PTCA, Percutaneous Transluminal Coronary Angioplasty; SD, standard deviation

Missing data: education, n=147; BMI, n=15; physical function, n=140; smoking, n=122; physical activity, n=2013; walking, n=2013

[1]Walking Pre-WHISH measurement is a median (IQR) of 3.1 (2.8-3.3) years prior to WHISH randomization.

eTable 3. *WHISH* PA-I Survey Responders, Overall and by Age at Randomization

		Overall		Age at randomization (years)					
		Number of women who participated		66-77 N returned/Total in age category		77-82 N returned/Total in age category		83-99 N returned/Total in age category	
	Total N	N	%	N	%	N	%	N	%
Survey 1 (2015)	23653	11621	49.1	4637/8243	56.3	3888/7800	49.9	3096/7611	40.7
Survey 2 (2016)	21623	11519	53.3	4636/7781	59.6	3929/7229	54.3	2954/6613	44.7
Survey 3 (2017)	20062	12620	63.0	5059/7375	68.6	4319/6759	63.9	3242/5928	54.7
Survey 4 (2018)	19201	13790	71.8	5545/7219	76.4	4782/6521	72.6	3463/5461	62.9
Survey 5 (2019)	18080	12800	70.8	5414/7037	76.9	4439/6193	71.7	2947/4850	60.8
Survey 6 (2021)	16625	11248	67.7	5150/6793	75.8	3980/5787	68.8	2116/4045	52.3
Survey 7 (2022-2023)	14294	9124	63.8	4483/6363	70.5	3219/5101	63.1	1422/2830	50.3
Survey 8 (2024)	12994	8417	64.8	4427/6105	72.5	2913/4653	62.6	1077/2236	48.2

Note: Percentages are out of the total number of survey respondents, not the number of respondents in each category.

eFigure 1. Self-reported Physical Activity Outcomes by Intervention Arm and Physical Activity (MET-hr/wk) at WHISH Enrollment

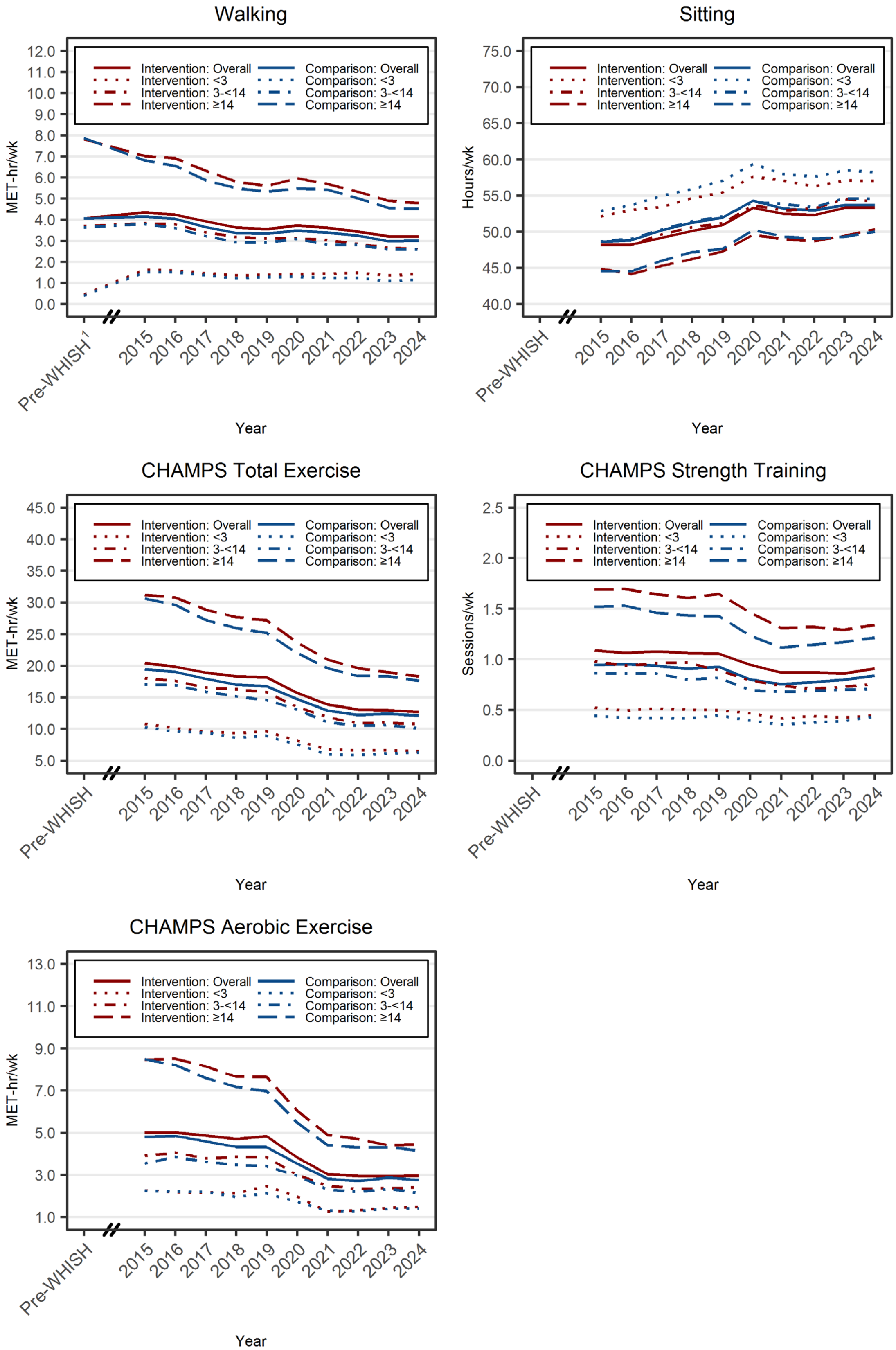

[1]Walking Pre-WHISH measurement is a median (IQR) of 3.1 (2.8-3.3) years prior to WHISH randomization.

Alt-Text: A composite figure containing five line graphs tracking self-reported physical activity physical activity and sitting trends from 2015 to 2024 for the Intervention (red line) and Comparison (blue line) groups, with separate line types for all participants (solid lines) as well as pre-trial physical activity (MET-hr/wk) levels of <3 (dotted lines), 3-<14 (alternating dash/dot lines), and ≥14 (dashed lines). The panels monitor Walking, Sitting hours, CHAMPS Total Exercise, CHAMPS Strength Training, and CHAMPS Aerobic Exercise. Across all panels, exercise metrics generally decline over time while sitting hours increase. The Intervention group consistently maintains higher average exercise levels and lower average sitting hours than the Comparison group throughout the study timeframe. Participants with higher physical activity prior to trial start consistently report higher exercise and less sitting.

Appendix A. WHISH Protocol

Official Title: Women's Health Initiative Strong and Healthy Study

NCT number: NCT02425345

Document name: Phase1_Phase2_Merged_PDFA for CTgov
[Version 080515 Version 2 = Phase 1. Phase 2 labeled as such.]

Document date last approved: January 27, 2021 [see Table of Contents for Phase 2: 1R61HL151885-01 (9/15/20-8/31/21); R33HL151885 (subsequent years)]

IRO REC'D OCT 25 2016

The Women's Health Initiative Strong & Healthy (WHISH) Trial

Protocol Version 2

IRO REC'D NOV 17 2015

IRO REC'D OCT 25 2016

The *Women's Health Initiative Strong & Healthy* (*WHISH*) Trial -

Physical Activity to Improve Cardiovascular Health in Women: a Pragmatic Trial

Section	Topic	Page
1.0	Introduction	2
2.0	Study Objectives	2
3.0	Background	3
4.0	Study Design	4
4.1	Schema	6
4.2	Inclusion Criteria	6
4.3	Exclusion Criteria	6
4.4	Ethical Considerations	6
4.5	Randomization and Blinding	7
4.6	Recruitment and Consent	7
4.7	Risks and Benefits	8
4.8	Early Withdrawal Plans	8
5.0	Outcomes	9
5.1	Outcomes of Interest	9
5.2	Collection of Outcomes	9
5.3	Timeliness of Outcomes Collection	10
6.0	Study Procedures	11
6.1	Vanguard Start-up	11
6.2	Intervention	11
6.3	Safety and Adverse Events	17
6.4	Data Collection	[illegible]
7.0	Statistical Plan	20
8.0	Data Management	22
9.0	Study Monitoring	22
10.0	Study Administration and Organization	23
11.0	Study Timeline	25
12.0	Publication Plan	25
13.0	Attachments	25
14.0	References	25

FHCRC IRB Approval
NOV 25 2015
Document Released Date

FHCRC IRB Approval
AUG 07 2015
Document Released Date

FHCRC IRB Approval
NOV 23 2016
Document Released Date

1.0 Introduction

The number of older Americans is projected to double to 88 million by 2050, with the majority being women, creating a public health mandate to identify effective interventions for preserving the health and independence of older women. Compelling evidence supports the importance of physical activity (PA) for reducing the risk of fatal cardiovascular disease (CVD), myocardial infarction (MI), and stroke, specifically ischemic stroke, as well as preserving physical and cognitive health and reducing functional limitations in older adults.[1] Despite the steady emergence of evidence-based interventions for increasing PA in older adults that can be delivered on a large scale, there is a lack of adequate randomized trial data showing that these interventions prevent clinical events or impact the public.[2]

The ***Women's Health Initiative (WHI) Strong & Healthy*** (***WHISH***) trial, a large-scale, randomized, controlled, pragmatic trial, is designed to address this critical gap. This multi-site clinical trial will be embedded in the ongoing WHI Extension 2010-2015 cohort of women, now aged 63-99 years. The trial tests the hypothesis that a centralized, public health intervention designed to increase and/or maintain PA levels will reduce major CV events (CVD death, MI, and stroke) in older women over approximately a 4-year period. Outcomes will be ascertained using established methods in place in the WHI Extension Study, including annual mailings with physician adjudication of events in a Medical Records Cohort of ~17,000 women, and use of Centers of Medicare & Medicaid Services (CMS) records for an additional ~34,500 participants, supplemented by National Death Index (NDI) for all. The WHI infrastructure, combined with innovative, evidence-based means to deliver the PA intervention at a population level, provide a unique opportunity to address this important question. Demonstrating that an inexpensive, easily delivered PA intervention can reduce the CV health burden of older women has major public health significance.

The *WHISH* trial applies state-of-the science behavioral principles and currently available technologies to deliver a physical activity intervention without face-to-face contact to ~21,000 older U.S. women expected to consent. It includes an algorithm-driven interactive voice response (IVR) system, built to complement the National Institute of Aging (NIA) *Go4Life®* Exercise & Physical Activity materials,[3] which provides tailored advice on how to achieve nationally recommended levels of PA and overcome barriers to exercise, with a means for self-monitoring and setting personal goals. The intervention builds upon evidence-based behavioral science principles and intervention components that have proven to be effective in increasing PA in older women, with innovative adaptive approaches to tailoring the delivery to meet individual (personal) needs.

2.0 Study Objectives

The specific aims of the *WHISH* trial are:

<u>Primary Aim</u>: Conduct a pragmatic, randomized controlled trial in ~52,000 older women enrolled in the WHI Extension Study allocating women to a tailored physical activity (PA) intervention or "usual activity" (observational control comparison) and following them for CVD outcomes over approximately 4 years.

> Primary hypothesis: A tailored PA intervention designed to improve levels of aerobic PA, combined with muscle strengthening, balance and flexibility exercises, and reduced sedentary behavior, will reduce major CV events (defined as a composite outcome of MI, stroke, and CV death), compared to "usual activity" controls.

<u>Safety Aims</u>: Evaluate whether the tailored PA intervention increases the individual risks of total clinical fracture, hip fracture, falls, and non-CVD mortality over the follow-up period.

<u>Secondary Aim</u>: Evaluate whether the tailored PA intervention reduces risk of selected exercise-linked secondary outcomes of tremendous importance to maintaining independence in older

women and potentially ameliorable through regular physical activity participation.

Secondary hypotheses: Women randomized to the intervention (compared to control) will have:

a. Lower rates of venous thromboembolic events (VTE) and peripheral artery disease (PAD);

b. Less loss of physical function and higher rates of ongoing mobility.

Secondary Safety Aim: Evaluate whether increased PA increases coronary revascularization (CABG or PCI).

3.0 Background

There is general consensus that physical activity (PA) is important for physical and cognitive health in older adults.[1] PA is associated with substantial health benefits for older adults, including lower risk of premature mortality from several chronic diseases (i.e., CVD and colon cancer) and lower incidence of functional limitations, i.e., impaired walking. Recent reviews estimate a 25-30% risk reduction for stroke with PA, particularly ischemic stroke. Evidence is also accruing that reducing sedentary behavior, such as prolonged sitting, is important in older adults. Both walking and vigorous exercise were associated with substantially lower cardiovascular event rates among nearly 74,000 WHI Observational Study participants aged 50-79 over a mean 3.2 years.[4] Prolonged sitting also predicted CV risk, independent of leisure-time physical activity in the WHI cohort and a combination of low PA and prolonged sitting (≥10 hours/day) augmented CV risk.[5] Aerobic exercise has also been shown to augment the treatment of lipid abnormalities, hypertension, diabetes, weight loss, and obesity and it is currently recommended as part of treatment regimens for hypertension[6] and arthritis.[7] Aerobic fitness training may also improve cognitive function among sedentary older adults.[8] In addition, resistance training helps maintain muscle strength and mass, bone mineral density, and functional capacity.

There is, however, no clinical trial evidence that increasing (or maintaining) PA or that decreasing inactivity will prevent or delay the onset of coronary heart disease (CHD), i.e. fatal and nonfatal MI and revascularization (CABG/PCI), or other CVD, (e.g., ischemic stroke, VTE, PAD) or prevent functional and health status declines known to occur with advancing age. Older women who choose to be active may differ from sedentary women physically, psychologically, and in other characteristics that may underlie both their lifestyle and better health. Nonetheless, national recommendations for physical activity in older women, i.e., the DHHS Guidelines for Older Adults,[1] promote a minimum PA dose, i.e., volume, or total weekly amount of PA, as a product of frequency (episodes/week), duration (time per episode), and intensity (level of effort per episode, i.e., light, moderate, vigorous, in METS), for all adults. Specifically, DHHS guidelines recommend that older adults: (a) engage in at least 150 minutes per week of moderate-intensity aerobic activity, such as walking, or 75 minutes per week of vigorous-intensity PA, or, if unable to achieve this level, because of chronic conditions, to be as physically active as one's abilities and conditions allow; (b) avoid inactivity, in particular, reduce sitting; (c) include muscle-strengthening activities that involve the major muscle groups at least 2 days per week; (d) If at risk for falling, include exercises to improve balance; and also, (e) follow principles of injury prevention.

Despite an extraordinary body of literature demonstrating the ability of exercise capacity to predict CVD risk, we have inadequate randomized trial data showing that interventions that improve exercise capacity prevent clinical events, and even if exercise interventions improve outcome in some people with low levels of exercise capacity, it is not clear that these interventions would have a substantial impact on the public. It has been recommended that the next phase of PA research emphasize large-scale randomized pragmatic trials to determine whether achieving or maintaining nationally recommended PA levels can reduce CVD in older adults at a population level.[2]

Demonstrating that a cost- and resource-efficient PA intervention could reduce the health burden of older women would be of major public health significance.

4.0 Study Design

WHISH employs a randomized consent design within the large cohort of WHI participants who have consented to ongoing follow-up. Using the WHI database, all key aspects of eligibility are assessed. It is estimated that about 52,000 WHI participants will be eligible for *WHISH* at the onset of the trial. Those considered to be eligible will be randomized in equal proportions (about 26,000 each) to the usual activity arm (no-intervention comparison) or the physical activity intervention arm. See Figure 1 for a schematic representation of the study design.

It is envisioned that towards the end of the enrollment period (end of 2015) several thousand more WHI women will be eligible for the *WHISH* trial, because while they do not have outcomes collected at the onset of the *WHISH* trial, they will have outcomes collected after they enroll in the COSMOS trial. The exact number of these women is unknown. These women will be enrolled at the end of the enrollment period, potentially increasing the number of subjects enrolled in *WHISH* by a few thousand participants.

Women assigned to the PA intervention arm will be mailed a letter from the Principal Investigator of the WHI Clinical Coordinating Center which informs them that Stanford University *WHISH* investigators will mail them "further written materials" designed to help them increase their daily activity, to meet national recommendations for physical activity for older adults unless they do not want to receive these mailings, in which case they will be instructed to call a 1-800 number. Thus, women who do not want to receive materials in the mail will actively decline, or "opt-out"; whereas, others will passively consent to receiving materials by mail from the Stanford *WHISH* PA intervention site. This initial mailing will include three items: (1) a cover letter from the WHI CCC Principal Investigator with information about *WHISH* trial and the 800-number to "opt out" of the *WHISH* PA Intervention; (2) *WHISHful Actions: National Recommendations for Physical Activity in Older Adults* (i.e., basic information on the national D.H.H.S. PA recommendations with health and safety tips); (3) the *Go4Life® pamphlet "Workout to Go: A Sample Exercise Routine from the National Institute of Aging at NIH"*.[3]. Therefore, all women assigned to the PA intervention will have been provided with the basic *WHISH* PA intervention goals to engage in regular physical activity. Of the women randomized to the PA intervention arm, it is estimated that only 5% (~1300) will opt-out, i.e., inform us at the outset that they do not want PA intervention mailings.

Participants randomized to the WHISH intervention who do not opt out will receive "further written materials" from the Stanford *WHISH* PA Intervention site in a **Welcome Packet** that will include seven items: (1) a cover letter with information on how to access the *Go4Life®* and *WHISH* websites, information on how to contact the Area Agency on Aging to learn about local PA facilities and resources for older adults, and how to stop future mailings by contacting the 800-#; (2) a slightly more detailed (*WHISH*ful Actions: Getting Moving) handout that expands on the goals of the *WHISH* PA intervention, informed by the DHHS recommendations; (3) a (*Go4Life®*) book that describes the benefits of exercise and physical activity for older adults and how to set exercise goals and stick with them (and information on how to obtain the Spanish version if desired); (4) a Survey requesting information on (i) current PA level and interest in increasing specific activities; (ii) preferred activities and use of walking aids or information on personal barriers to exercise; (iii) preferred communication mode, i.e., mail, Email, website, etc., and interest in receiving a (*Go4Life®*) DVD on how to exercise; (5) suggestions/instructions on how to develop an initial Action Plan; (6) postage paid monthly physical activity logs to record minutes of aerobic exercise each day, strengthening exercises, balance and flexibility exercises, hours of sedentary behavior (particularly, sitting) and an assessment of relative intensity, i.e., Borg scale; and (7) description and a consent form regarding the additional telephone "health coach" support program Stanford

will offer, as an automated interactive voice response (IVR) system, for which they can consent through a telephone consent process or by returning a written consent.

Whether women actively consent to the IVR support or not, they will continue to receive mailings and information on how to access the *Go4Life®* and *WHISH* websites, unless they choose to opt-out of receiving the written materials at any time. As soon as they engage with the *WHISH* PA intervention team by returning the survey, they will be provided with a pedometer and information on how to record daily steps and activity on *WHISH* Activity Calendars, with instructions to return these on a monthly basis. We anticipate that about 2/3 of participants (~15,000) will actively consent to additional support with the IVR "Health Coach". These women will receive special instructions on how to access the IVR Training program (IVR script) and staff will provide assistance, as needed, to ensure they know how to take advantage of this program. Women assigned to the comparison arm will not receive a Welcome Packet mailing or other mailed materials. Both intervention and comparison participants will be followed according to the WHI Extension Study protocol.

The *WHISH* Data Coordinating Center (DCC), located at the WHI Clinical Coordinating Center at the Fred Hutchinson Cancer Research Center conducts all activities related to screening, randomizing, and mailing of the initial information letter regarding the *WHISH* trial and materials described above and below to women assigned to PA intervention. The Stanford Clinical Coordinating Center (CCC), i.e., the *WHISH* PA intervention site, mails the Welcome Packets and delivers the physical activity intervention, manages the consent process and all aspects of the algorithm-driven interactive voice response system (IVR) "Health Coach" program, and monitors and works to maintain and/or improve adherence to the PA intervention.

The steps of the *WHISH* trial are, in brief:

1) Using the WHI database, the *WHISH* DCC screens all WHI Extension Study women to select the women eligible for *WHISH* randomization, based on study inclusion/exclusion criteria.
2) The DCC randomizes all eligible women to either the PA intervention or comparison control arm.
3) The DCC mails the initial introductory letter and other materials to women assigned to the PA intervention group to inform them that Stanford will provide the PA intervention unless they opt out by calling the 800-number.
4) If after one month the DCC does not receive an opt-out message, the Stanford CCC receives notice that a *WHISH* PA intervention participant has (passively) consented, and the Intervention protocol will commence.
5) The Stanford CCC mails consented PA intervention women a Welcome Packet which contains the *Go4Life®* book and information on the PA goals, a survey, plus a consent form regarding the additional telephone (IVR) support program, which they can either sign and return by mail or call into an IVR system that allows for telephone-based consenting. See4.6. Recruitment and Consent for details of the Welcome Packet.
6) The Stanford CCC conducts the study intervention:
   - Activities for "mailings only" (primary) PA intervention include: mailed materials + website: all basic written and *Go4Life®* materials; pedometers to track steps; logging of monthly PA on postage-paid postcards; newsletters.
   - Activities for "Health Coach" (additional support) IVR-based program: IVR-delivered advice and support, PA self-monitoring; and, as needed, staff contact (live advisor).

- At any time during the study it will be possible for participants to consent to the additional IVR support. Participants can also request temporary or permanent cessation of mailings or IVR contacts at any time during the study.

7) The DCC mails the baseline *WHISH* questionnaire to all (usual activity comparison and intervention) participants in their annual medical history update mailing.
8) Both intervention and comparison group participants will be followed as described in the WHI Extension Study protocol.

4.1 Schema

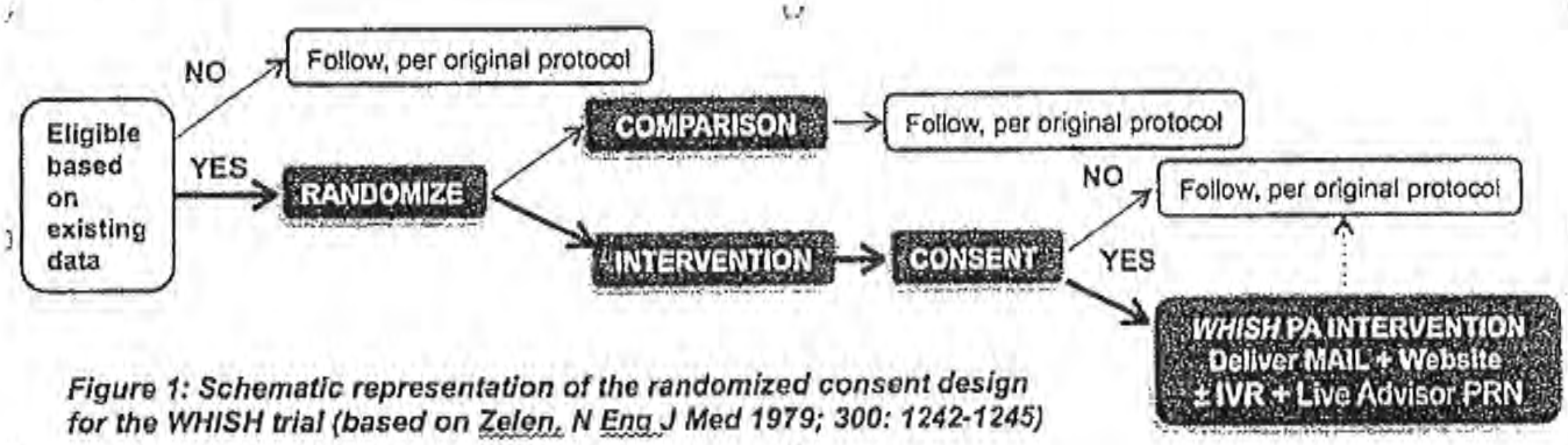

Figure 1: Schematic representation of the randomized consent design for the WHISH trial (based on Zelen, N Eng J Med 1979; 300: 1242-1245)

4.2 Inclusion Criteria

- Currently enrolled in the WHI Extension Study
- Known to be alive
- CV outcomes will be available (i.e., enrolled in the WHI Records Cohort [MRC] or linked to Medicare data)
  - WHI subjects who have their outcomes collected because they are enrolled in the COSMOS trial are also eligible for the *WHISH* trial, and will for the remainder of this protocol be considered as part of the MRC cohort.

4.3 Exclusion Criteria

Women will be excluded if they cannot realistically be expected to participate fully in the intervention due to:

- Inability to walk
- Dementia
- Residing in a nursing home

4.4 Ethical Considerations

Enrollment and randomization are deemed ethical in that key materials provided to participants randomized to the PA intervention arm are available to the general public and therefore to participants randomized to the "usual activity" comparison.

Women randomized to the PA intervention arm who participate in the "Mailings only (+website)" program will have passively consented. Thus, not "opting out" serves as the consent for the WHISH Trial whereas a signed (or telephone entered) consent is required to receive and engage in the *WHISH* IVR "health coach" program. Women randomized to the "usual activity" comparison will not receive the *WHISH* intervention and will be followed per standard WHI protocol. It should

be noted that all *Go4Life®* materials and information regarding DHHS recommendations for physical activity for older adults that will be mailed to these women are available to the general public, and thus to all women in the comparison group, for free. Women randomized to the PA intervention arm who participate in the IVR "Health Coach" program will provide informed consent (by mail or phone).

4.5 Randomization and Blinding

The *WHISH* DCC screens all WHI Extension Study women using the WHI database to select the women eligible to be randomized, based on inclusion/exclusion criteria. Eligible women are then randomized to one of two groups using a 1:1 allocation scheme:

1) Physical Activity (PA) Intervention group (intervention)
2) Usual Activity group (comparison)

To establish and equalize the "baseline" in both the intervention and comparison groups, a mailing order/date is randomly assigned at the *WHISH* DCC; this date is used to establish the baseline for determining start of follow-up in both groups.

To protect blinding of Stanford Outcomes staff to *WHISH* intervention randomization assignment, the DCC will ensure that the WHI Outcomes database is not accessible to Stanford *WHISH* intervention staff. In addition, if the Stanford Intervention staff members receive information about disease events from women enrolled in *WHISH*, this information will not be used in any way to inform outcomes ascertainment in the *WHISH* Intervention group. This protects against bias in outcomes ascertainment from additional contact with the *WHISH* Intervention group.

As the outcomes collection is very different for women in the Medical Records Cohort and those that are not in the MRC (see Sec 5.2), women will be stratified on being in the MRC or not. As exercise levels differ considerable by age and region, the randomization will also be stratified by age (divided in tertiles, <76, 76-81, 82+ at time of randomization) and region (North-East, South-East, Midwest, West). The primary analyses will be stratified by these 2x3x4=24 strata.

4.6 Recruitment and Consent

Women randomized to the PA intervention group are contacted by mail by the DCC to obtain (passive) informed consent. This initial mailing will include three items: (1) a cover letter from the WHI CCC Principal Investigator with information about *WHISH* trial and the 800-number to "opt out" of the *WHISH* PA Intervention; (2) *WHISHful Actions: National Recommendations for Physical Activity in Older Adults* (i.e., basic information on the national D.H.H.S. PA recommendations with health and safety tips)*;* (3) the *Go4Life® pamphlet "Workout to Go: A Sample Exercise Routine from the National Institute of Aging at NIH".*

Women randomized to the usual activity group are not contacted for consent.

Women who do not "opt out" will receive the following from the Stanford University *WHISH* PA Intervention site:

The Welcome packet:

1) a cover letter with information on how to access the *Go4Life®* and *WHISH* websites, how to contact the Area Agency on Aging to learn about local PA facilities and resources for older adults, and how to stop future mailings by contacting the 800-#;
2) *WHISHful Actions: Getting Moving (a slightly more detailed handout from what was included in the mailing from the WHI CCC, providing details on the goals of the WHISH PA intervention);*

3) the *Go4Life®* book *"Exercise & Physical Activity: Your Everyday Guide from the National Institute on Aging" (with information on how to obtain a Spanish version, if desired;*
4) A Survey (to be returned to Stanford) requesting information on (i) current PA level and interest in increasing specific activities; (ii) preferred activities and use of walking aids or information on personal barriers to exercise; (iii) preferred communication mode, i.e., mail, Email, website, etc., and interest in receiving a (*Go4Life®*) DVD on how to exercise;
5) Suggestion/Instruction on setting personal goals and developing an achievable Action Plan;
6) Postage paid monthly physical activity logs to record minutes of aerobic exercise each day, strengthening exercises, balance and flexibility exercises, hours of sedentary behavior (particularly, sitting) and an assessment of relative intensity, i.e., Borg scale; and
7) A description of the *WHISH* "Health Coach" (IVR) additional support program and consent form to join this optional study component.

At subsequent mailings women in the "Mailings only (+website)" group will again be offered the opportunity for additional support through the "Health Coach" IVR-based program.

See Section 6 for additional details on the levels of study participation, including the study materials and specific intervention activities for each group.

4.7 Risks and Benefits

The PA intervention was designed to promote the key DHHS guidelines for Older Adults and to ensure that the risks for participating in this physical activity program are minimized to the extent possible. Participants' PA prescriptions will be tailored based on their baseline levels of endurance capacity, strength, balance, and behavioral readiness for the proposed regimen. *WHISH* participant PA information, collected via the "Welcome Packet" survey will be used initially to tailor recommendations for individual intervention starting points. Walking will be the primary mode of PA; other forms of activity (e.g., stationary cycling, swimming, dancing) will be encouraged, particularly when regular walking is medically contraindicated. Participants who cannot do moderate levels of PA because of chronic conditions will be encouraged to be as physically active as their abilities and conditions allow, per DHHS guidelines. Materials will be provided for women to share with their health care providers, using American College of Sports Medicine "Exercise as Medicine" messages.

The major risks of participating in a physical activity program are temporary fatigue and muscle soreness. Cardiovascular, orthopedic (i.e., foot or leg problems), or bone and joint complications can occur during exercise training, but serious complications are rare if a properly designed exercise regimen is followed. Unless there is damage due to previous injury, serious orthopedic complications are uncommon as a result of the exercise programs being recommended. Risk of a sudden heart attack is low, similar to that of other activities of daily living.

The direct benefits of participating in a regular exercise program may be increased fitness, flexibility, and balance, as well as a possible reduction in the risk of developing cardiovascular disease. Women will also learn more about monitoring and improving their fitness level in general. The information learned from this study will help investigators learn about the effects of physical activity on the health of older women.

4.8 Early Withdrawal Plans

As with any WHI study, women enrolled in the PA intervention will be allowed to discontinue participation in the *WHISH* study at any time.

If participants in the Medicare linkage group drop out of Medicare for any reason, so that outcomes data no longer is obtained, Stanford WHI Western Regional Center Outcomes staff will

obtain medical records and process the outcomes for WRC participants as the staff are doing within the broader WHI for the western region. The DCC Outcomes staff will conduct this activity for such women in the remaining WHI regions.

5.0 Outcomes

5.1. Outcomes of Interest

Primary Outcomes

Composite cardiovascular disease (CVD) including definite and probable nonfatal MI, stroke, and CHD and cerebrovascular (CV) death. Definite and probable nonfatal MI require overnight hospitalization and are defined according to an algorithm based on standardized criteria using cardiac pain, cardiac enzymes and troponin levels, and ECG findings. CHD death is defined as death consistent with underlying cause of CHD plus one or more of the following: hospitalization for myocardial infarction within 28 days prior to death, previous angina or myocardial infarction, death due to a procedure related to CHD, or a death certificate consistent with underlying cause of atherosclerotic CHD. Strokes and cerebrovascular death were classified by neurologists according to the Trial of Org 10172 in Acute Stroke Therapy (TOAST) and Oxfordshire subtype classifications.

Safety: Total clinical fracture, hip fracture, falls, and non-CVD mortality.

Figure 2 – Outcomes collection of the WHISH trial

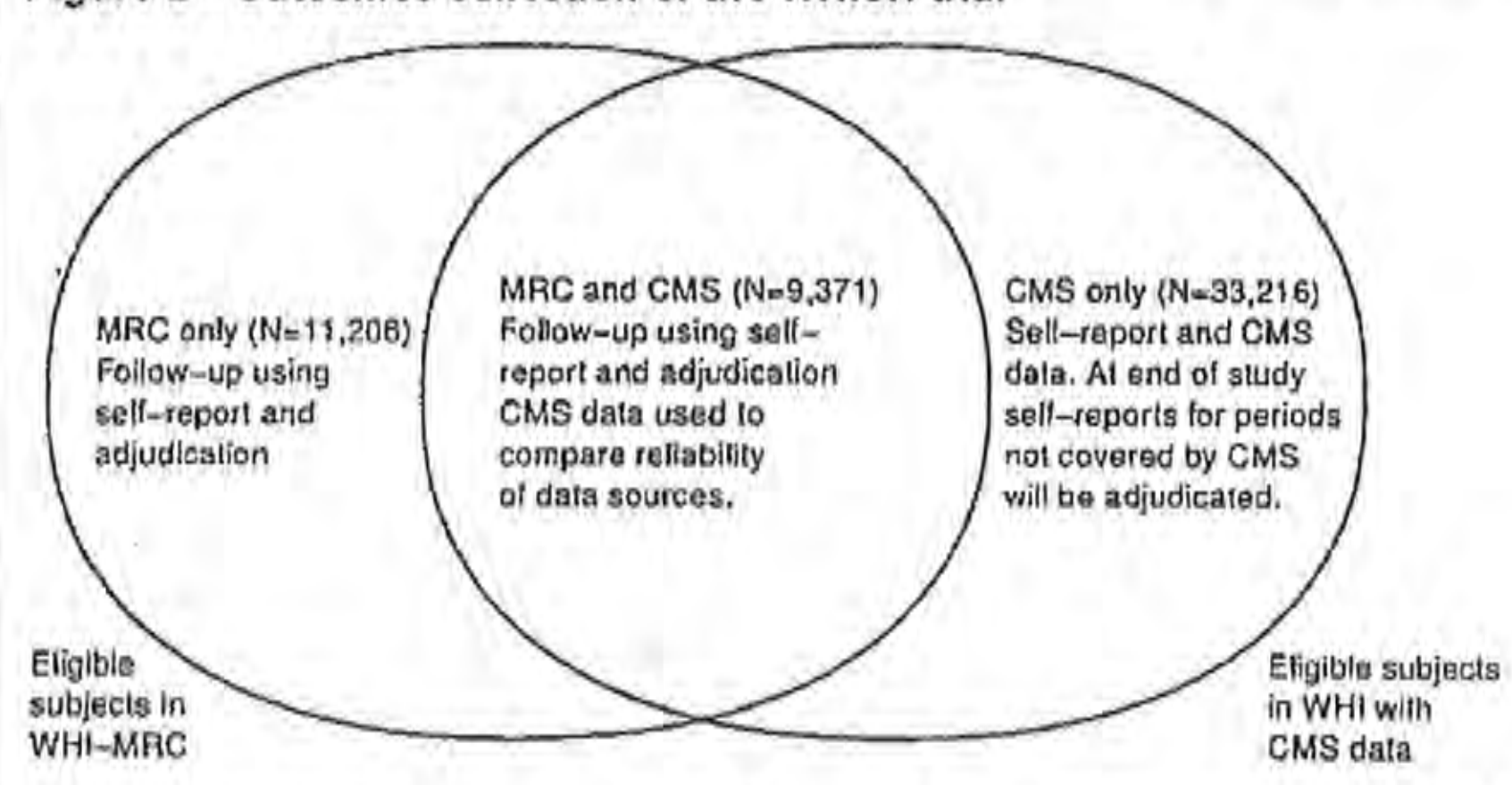

Secondary Outcomes

Venous thromboembolic events (VTE) and peripheral artery disease (PAD), physical function, mobility and independence

Safety: Coronary revascularization (CABG or PCI).

5.2. Collection of Outcomes

The primary and secondary outcomes will be collected in both the intervention and usual activity comparison groups through the main WHI Extension Study.

Outcomes sources include adjudicated self-report among the WHI Medical Records Cohort and Medicare administrative data. These sources will be supplemented by data from the National Death Index (NDI).

Adjudicated self-reports from the WHI Medical Records Cohort

WHI physician-adjudicated self-reports by self or proxy will be collected from the WHI Medical Records Cohort (MRC) annually per WHI Extension protocol (September 13, 2011) and in Curb.[9] Briefly, WHI participants who re-consented to the 2010-2015 Extension are being followed annually, primarily by mailed questionnaires from the WHI-CCC, to collect data on self-reported health events and related conditions, using a modification of the procedures employed previously, with approximately one-twelfth of the participants receiving the mailing each month. Mailing contents vary by year, as indicated in the Extension Study protocol, but always include *Form 33* -

Medical History Update and return envelope. The WHI-CCC manages all of the outcomes data, thus *WHISH* study analyses will require extensive collaboration with the WHI-CCC, particularly for outcomes that require Medicare linkage data. See Figure 2. MRC participants have full outcomes ascertainment and documentation, including: adjudicated medical records for CHD, CABG/PTCA, heart valve problem/repair, aortic aneurysm, stroke, carotid artery disease, heart failure, atrial fibrillation, VTE, PAD, Cancer (all sites except non-melanoma skin cancer), hip fracture, and death from any cause; limited document review for capture of other events/ICD9 coding; and self-reported angina, transient ischemic attack (TIA), other fractures and several other age-related diseases, including chronic obstructive pulmonary disease (COPD), diabetes mellitus requiring therapy (insulin, pills, diet), hypertension requiring therapy, intestinal or colon polyps or adenomas, macular degeneration, osteoarthritis or arthritis with aging, Parkinson's disease, systemic lupus erythematous, moderate/sever memory problems (dementia/Alzheimer's), falls, and hysterectomy.

All WHI physician and neurologist adjudicators, and outcomes staff at the WHI regional centers and the WHI-CCC will be blinded for *WHISH* randomization status; in particular there will be no contact between the Stanford regional center outcomes staff and the staff of the *WHISH*-CCC at the same institution.

<u>Medicare administrative data (Center for Medicare & Medicaid Services, CMS)</u>

Outcomes for the WHI subjects who are enrolled in Medicare Part A+B or Part A only, and who are not enrolled in an HMO, will be obtained from CMS data. During the last year of the *WHISH* trial, to circumvent delay in obtaining Medicare data, WHI non-adjudicated self-report will be adjudicated according to standard WHI procedures. See Figure 2.

Table 1: Expected number of months of follow up.

	MRC (N~17000)	Medicare only (N~34500)	Average
Feb 2016	8	0	2
Feb 2017	20	2	8
Feb 2018	32	14	20
Feb 2019	44	26	32
Feb 2019	44	44 (including outcomes sweep)	43
Feb 2020	56	56 (including outcomes sweep)	56

A proportion of women drop in and out of Medicare criteria for this study (e.g., because they drop in and out of HMOs), and we will build in funds to collect medical records and obtain full outcomes (as is currently done for MRC by the WHI) for self-report cohort (SRC) participants who drop out of Medicare . The SRC is followed with outcomes ascertainment for the same events as the MRC, but limited to self (or proxy) report or passive follow-up sources of information, with the exception of cancer, for which all sites have adjudicated medical records except for non-melanoma skin cancer.

<u>Overlap of WHI adjudicated self-report and Medicare administrative data</u>

Overlap in the subgroups of women with both Medicare fee-for-service outcomes data and WHI medical records adjudication of self-reported outcomes will serve as cross-validation between the two outcomes sources. See Figure 2.

<u>National Death Index (NDI) data.</u> WHI carries out annual NDI searches, which allow us to identify fact and cause of death for all subjects in the *WHISH* trial. NDI data is available annually, approximately at the same time as Medicare data.

5.3. Timeliness of Outcomes Collection

Self-reports on the WHI *Form 33 - Medical History Update* are collected annually. Documentation and adjudication takes on the average about 2-3 months, thus at any time there will be an 8-9 months backlog in outcomes collection (data for a limited number of self-reports that have not yet been fully adjudicated will be avail-able in addition to the adjudicated outcomes). Medicare data is anticipated to be available with approximately 12-18 month after the calendar year (though recently it has never been available earlier). For example, data on outcomes in 2014 will be available in

early to mid 2016. Thus, in early 2019, at the end of the study Medicare data through 2017 is anticipated to be available. Therefore we will plan to adjudicate outcomes that are self-reported during 2018 and 2019. At the end of the *WHISH* study we will have an average of approximately 44 months follow-up while with an additional year we will have 56 months of follow-up (Table 1).

6.0 Study Procedures

6.1. Vanguard Start-up to Optimize Intervention Elements

A vanguard cohort (N~1000) will be initiated 2 months before implementing the full trial intervention. During the vanguard period, investigators will test refinements and cost-effective augmentations to the intervention in women who are not meeting adherence goals after an initial 3-month adoption period. The aim is to determine participant characteristics that may be important to "tailoring" the intervention to individuals, i.e., *a personalized approach*.

During the Vanguard start-up, participants who either do not interact with the IVR sufficiently or consistently fall below their stated PA goals will be randomly assigned to a modified (e.g., more intensive) IVR contact schedule, as well as more varied and personalized motivational messages, to ascertain how well such refinements improve their IVR engagement and PA goal attainment. These "mini-tests" of program refinements help optimize the amount of IVR contact and breadth of motivational messages (e.g., in response to participant-identified additional PA barriers) going forward in the trial.

6.2 Intervention

The intervention is based upon a social cognitive model of health behavior acquisition and maintenance. Such strategies, combined with those from the trans-theoretical model serve as the evidence-based framework for the *WHISH* personalized stepped-care intervention approach.

The physical activity (PA) intervention will promote the Department of Health and Human Services guidelines for Older Adults of at least 150 minutes per week of moderate-intensity aerobic activity, such as walking, or 75 minutes per week of vigorous-intensity PA (noting that relative intensity varies by fitness level). Participants who cannot do moderate levels of PA because of chronic conditions will be encouraged to be as physically active as their abilities and conditions allow, per DHHS guidelines.

Women will be instructed in the following way:

- perform moderate aerobic PA in episodes of at least 10 minutes, preferably spread throughout the week;
- include simple muscle-strengthening activities that involve the major muscle groups;
- include exercises to improve balance and flexibility, each to be done on 2 or more days a week in ~10- minute bouts or more;
- avoid inactivity, with emphasis on reducing sitting throughout the day; and
- heed instructions for injury prevention that will be provided.

Mailed materials (+website, if relevant) will be the primary means of delivering the PA intervention. If participants would like (and consent to) additional support, they may take advantage of an algorithm-driven, telephone-based interactive voice response (IVR) system,[11,12] custom built to complement the *Go4Life®* and *WHISH* materials. A stepped care approach will be implemented to support three levels of intervention. Multiple delivery channels will be offered in support of individual women's needs and preferences, e.g., mailings, website, IVR, live advisor help, as needed.

All women randomized to the *WHISH* intervention will receive information about being physically active, with safety tips including injury prevention. Women who consent to the *WHISH* intervention will receive additional materials, instructions and support.

See Table 2. Theory-derived elements of *WHISH* Intervention for a tabular presentation of the intervention.

Table 2. Theory-derived elements of *WHISH* Intervention

Intervention Elements	Full Program			Adherence Support for Full or mail only + website, PRN
	Mail only +website program			
	Mail	*WHISH* Website	IVR	Live Advisor
**Social Cognitive Theory**				
Consciousness Raising	NIA *Go4Life®* materials (pamphlet, book, DVD)	Educational content library	Tip sheets mailed on demand	Tip sheets mailed on demand
Personalized Feedback	Printed feedback sent with reply cards	User profile to track PA, creates charts & tables	Audible feedback on performance, success in reaching goals	Live feedback on historical performance
Self-Efficacy through personalized goal-setting & attainment	Recommends goal ranges for different ability levels	Recommends achievable goal ranges	Suggests progressively challenging yet attainable goals	Advisor suggests progressively challenging yet attainable goals
Self-Regulation	Mail-back cards designed to collect self-monitored activity; participant asked to write goals	Uses web to track activity; site generates real-time feedback; participant sets goals on profile	Participant enters physical activity & gets immediate feedback; asked to enter goals for future calls	Participant reports activity; advisor gives immediate feedback; Participant asked to specify goals for next contact
Social Support	Periodic articles about importance of discussion with friends/family	Informational content on how to involve others for support	"Barrier" menu provides problem-solving ideas for enlisting support, asking for help	Advises on how to involve others, helps problem-solve who can be a support person
Social Modeling	Spotlights different participants who are role models for relevant achievements	Spotlights relevant participants	Relates stories from other participants	Refers to Mail, Web & IVR examples; encourages identifying role model
Environmental Management	Periodic articles on barriers & resources; tips offered; referred to website for more info.	Advises on local resources/facilities	"Barrier" menu provides problem-solving ideas for environ. challenges	Advises on local resources/facilities
**Trans-theoretical Model (TTM)**				
Current Assessment	Participant self-assesses current activity, receives specialized information	Assesses current activity, readiness to do more	Assesses current activity & readiness to do more	Assesses current activity & readiness to do more
Stage-Based Tailoring	Participant continues to receive specialized information based on mail-back cards	Site suggests cognitive & behavioral content matched to TTM stage of readiness	Matches cognitive & behavioral content to TTM stage of readiness	Matches cognitive & behavioral content to TTM stage of readiness
Goal Matching	Pt. encouraged to specify goals via mail-back cards	Site suggests goal matched to current TTM stage	Negotiates goal based on current TTM stage	Negotiates goal based on current TTM stage

6.2.1. Program Levels: Primary (Mailings Only + website) plus Additional Support

Women will be involved at one of two levels of participation:

1) Mailings only (+ website). Participants receive all basic written and *Go4Life*® materials, i.e., *Go4Life*® pamphlet, book (available in Spanish) and DVD and information on how to access the *Go4Life*® and *WHISH* websites; pedometers for counting steps (if they return the survey and/or PA log); and newsletters. Participants are asked to log their PA on (pre-paid) postcards that they will mail to the Stanford CCC at regular intervals.
2) Additional Support through the *WHISH* telephone-based "Health Coach" program, i.e., IVR plus mailings/website. Participants who consent to this additional support will receive the *Go4Life*® materials provided to the mailings only group and tailored mailings, plus be engaged in the IVR-based advice and support program which includes instructions for using the IVR and entering PA data into the IVR.

6.2.2. Intervention Levels: Stepped Care Intervention

The intervention will be presented at three levels as part of stepped-care. Women will be encouraged to progress to the next level as they master lower level activities.

1) Getting Started. Participants' PA prescriptions will be tailored based on their baseline levels of endurance capacity, strength, balance, and behavioral readiness for the proposed regimen. WHI participant PA information, collected via "Welcome Packet" surveys will be used initially to individually tailor recommendations for intervention starting points. With the introductory welcome packet, suggestions of where to start, based upon these data, will be given.
2) Moving forward. For the Mailings Only intervention group, ongoing PA information will be regularly recorded via pre-paid mail-back cards. For the additional support group, PA information collected during each IVR contact will be automatically transferred to an electronic database, with automated weekly reports being generated for intervention staff to monitor. This information will be used to determine when it's time to move forward.
3) Achieving or maintaining higher levels. As with moving forward, information collected during the intervention will assist participants in determining their point of maintenance or achieving higher levels of PA.

6.2.3. Intervention Delivery Channels: Materials, Interactive Voice Response (IVR) and Live Staff Help

Intervention delivery choices include materials (written and DVD), IVR, and live staff help. Delivery of the intervention will occur with a consent packet and post-consent welcome and intervention packets.

6.2.3.i. Materials provided to women assigned to the PA intervention will receive an *Initial Mailing from WHI CCC* after randomization to PA intervention:

- Cover letter describing *WHISH* and explaining the "opt out" option (800=#)
- *WHISH*ful Actions: National Recommendations for Physical Activity in Older Adults
- *Go4Life*® pamphlet, *"Workout to Go: A Sample Exercise Routine from the National Institute of Aging at NIH"*[®]

Welcome Packet from the Stanford *WHISH* PA intervention site, after obtaining "passive" consent, women

- Cover letter which welcomes them and reminds them of the 800-number "opt out" option
- WHISHful Actions: Getting Started (full version) provides additional guidance on how to initiate exercise safely
- *Go4Life®*: Exercise & Physical Activity: Your Everyday Guide book (available in Spanish upon request)
- Instructions on how to develop a realistic Action Plan, i.e., set personal goals
- Mail back survey regarding health issues, physical function, and current activity that may be relevant to tailoring future mailings
- Postage paid monthly physical activity logs to record minutes of aerobic exercise each day, strengthening exercises, balance and flexibility exercises, hours of sedentary behavior (particularly, sitting) and an assessment of relative intensity, i.e., Borg scale
- Information on the IVR "Health Coach" program and consent form to be signed and returned by mail or completed by telephone (IVR script)

As soon as a woman engages in the intervention by mailing back the survey and/or a PA log, she will receive a pre-programmed and ready to wear Omron pedometer, with instructions on its use.

Welcome to the *WHISH* "Health Coach" (IVR) Packet. After women consent to the IVR "Health Coach" (additional support) Program) they will receive the following materials:

- Survey question to provide information on the preferred day of the week and time of day for IVR calls;
- *WHISH* IVR PA Calendar (with tear off sheets) to enable the participant to record her PA data exactly as the IVR will request it, including "pedometer steps";
- Information on how to call into the IVR for an "orientation and training" call, which will talk each participant through the steps to enable her to familiarize herself with the system and data entry cues;
- A two-week follow-up phone call for participants who have not called into the IVR within two weeks of the Welcome to the *WHISH* "Health Coach" (IVR) Packet mailing; and
- A week after she has completed the training call (with the IVR or staff), the "Adopting Physical Activity" 3-month period will begin with the first official IVR call.

Intervention Packets. During the intervention period, additional written materials will be developed for the *WHISH* participants' *health care providers,* using American College of Sports Medicine "Exercise as Medicine" messages. Health Care providers will be encouraged to reinforce the participant's adoption of a more active lifestyle. Materials will also be developed to promote activity with *friends, neighbors and family,* e.g., grandchildren, and facilitate social support. These will be mailed quarterly with newsletters.

6.2.3.ii. Interactive Voice Response (IVR).

We will use an algorithm-driven interactive voice response (IVR) system, built to complement National Institute on Aging (NIA) *Go4Life®* Exercise & Physical Activity materials, to provide tailored advice on how to achieve nationally recommended levels of PA and overcome barriers to exercise, with a means for self-monitoring (IVR-based logging activity) and setting personal goals with feedback.

An initial "orientation" IVR call will walk each participant through the steps to enable her to familiarize herself with the system and data entry cues.

IVR Intervention Phases: Adoption and Maintenance

Adopting Physical Activity. This 3-month period will begin one week after the IVR orientation by starting with the first official IVR call. IVR calls will occur weekly for the first six weeks, then every other week for the next six weeks, to total 9 calls in the first 3 months.

Maintaining PA. After the 3-month Adopting PA phase, IVR calls will occur an average of once a month for the remainder of the trial (with some personalization of the call schedule based on participant preferences).

6.2.3.iii. Live Staff Help.

Upon request (via a ***1-800 helpline)***, *WHISH* staff (live staff help) will contact a participant who has any questions regarding the study, PA goals, or IVR.

6.2.4. PA Components

The PA intervention will consist of a multimodal activity program of aerobics, balance, strength, flexibility. The intervention will involve encouraging participants to increase all forms of PA throughout the day and to decrease sedentary time, such as sitting. This may include activities such as leisure sports, gardening, use of stairs instead of escalators, leisurely walks with friends, and less use of remote control devices.

- Aerobics. Walking will be the primary mode of PA for impacting the primary outcome of major CV events. Other forms of endurance activity (e.g., stationary cycling, swimming) will be encouraged when regular walking is contraindicated medically or behaviorally. Participants will be instructed on a brief warm-up and cool-down period surrounding their PA.
- Flexibility. Participants will be instructed to complete simple flexibility exercises following each bout of walking.
- Balance training. Balance training will be introduced as a complement to the endurance and strength components. Participants will be instructed during the initial phase of the program to complete a ~10-minute routine that focuses primarily on overall balance and agility.
- Strength. After balance training is introduced, simple upper and lower extremity strengthening exercises will be added. As done in other strengthening programs for older adults, instructional materials (e.g., DVDs, printed materials) will be offered to participants to reinforce the strengthening component.
- Decreased Sedentary Time. Throughout the program, at all levels of participation and intervention, participants will be encouraged to spend less time sitting. For example, when watching television, not to use (or use less) a remote control device, or when working at the computer or doing stationary activities to get up and stretch and move periodically.

6.2.5. Intensity: Stepped Care

Participants will be introduced to PA in a structured, progressive way such that they begin with lighter intensity and gradually increase over the first 6-8 weeks. Ratings of self-perceived exertion (RPE) will be used as a method to regulate PA intensity. Borg's scale of 6 to 20 RPE represents an effort spectrum from very very light to very very hard.

- Aerobics. Walking at a moderate intensity will be promoted. Participants will be asked to walk at an RPE intensity of 13 (activity perception somewhat hard) and will be discouraged from exercising at levels that approach or exceed 15 (hard) or drop to 11 (fairly light) or below.
- Strength. After 2 weeks of familiarization, participants will be encouraged to perform the strengthening exercises at ~13 on the RPE scale. Participants will be encouraged to record, on the *WHISH* calendar, a specific RPE for each active muscle group being targeted.

6.2.6. Targeted PA Frequency and Duration: Stepped Care

The national PA goal for older adults (i.e., 150 minutes/week of moderate-intensity PA like walking supplemented with strengthening and balance/flexibility exercises 2 or more times/week) will be approached in a *progressive manner* across the intervention period. There are multiple ways that the goal can be achieved, based on the physical abilities and constraints of each participant. A "stepped" approach" will be utilized as a tailored, resource-sparing approach to intervention.

As part of this approach, information will be targeted to women at each of the following PA levels, based upon their current PA levels reported via IVR and postcard logs:

1) very sedentary (<100 MET min/week): encourage getting up, walking, and balance with a light strengthening component (to improve on baseline fitness and functional levels)
2) somewhat active, but below the guidelines (100-500 MET min/week): increase overall PA (incidental and structured) as discussed above
3) meeting the guidelines (>500-1200+ MET min/week): encourage maintenance of existing PA and promote the often missing strength and balance components.

6.2.7. Adherence and Support: Stepped Care

The IVR PA information collected during each contact will be automatically transferred to an electronic database, with automated weekly reports being generated for intervention staff to monitor. Similarly, in the Mailings Only intervention group, ongoing PA information will be regularly recorded via pre-paid mail-back cards and responded to by staff. This information will be used to trigger delivery of the supplemental IVR or staff-based contacts.

1) Basic Support. Participants who complete ~80% of IVR contacts (or mail back ~80% of postcard logs) or who achieve their PA goals will receive small, inexpensive incentives, such as *WHISH* visors for sun protection, bags for transporting shoes, coupons to purchase light hand weights, and other exercise-related items.
2) Higher Support. If a participant fails to interact with the IVR system over two consecutively scheduled calls, or reports ≤70% of her personal PA target in the IVR, she will be contacted either by the IVR (with a special reminder message to re-engage with the system) or, if IVR nonresponse continues, by study staff to encourage PA participation through providing tailored advice for overcoming specific barriers to PA, as well as IVR re-engagement. Participants always have the option of calling into the IVR more frequently if desired. Similarly, if participants in the Mailings Only group fail to return two consecutive mail-back cards, or report achieving ≤70% of their personal PA targets, staff will similarly follow up by phone. Participants will also have access to a toll-free HELP line to leave messages for *WHISH* staff if they have technical difficulty with the system (e.g., forget the IVR telephone number). A live staff person retrieves these messages and responds

within 48 hours. Additionally, community resources will be identified to encourage social support and group motivational techniques.

3) High Support (Live Advisor Help). If the participant still demonstrates a need for additional support due to under-utilization of the IVR system or the interactive mailings system, a research staff member will phone the participant to problem-solve strategies for re-engaging with the IVR or mailings systems, as well as providing additional tailored advice/encouragement for PA behavior change when needed.

6.2.8. Intervention Feedback and Assessment

1) Mailings Only (+ Website) Group. PA logs will be provided as pre-paid mail-back postcards that will be regularly data-entered by *WHISH* staff. Staff will call participants who do not mail back at least 70% of their cards and collect the information by telephone, if possible
2) Additional Support (IVR) Group. During each IVR system phone contact, women will report information on PA type and amount, along with pedometer steps. To track their PA behavior between IVR calls, women will be provided with sets of calendars (that can be posted on their refrigerators or by their phone) to record their daily activity, as a self-monitoring tool. (On-line versions and means to enter the data and download the pedometers will also be available). The IVR system is designed to reliably collect this information. It will also invite participants to report on any difficulties they encountered, and encourage them to take advantage of the tailored messages built into the IVR, which includes the ability to request contact with a staff person through an 800 number. The IVR system automatically summarizes the data in an electronic database that can be readily accessed by intervention staff. Upon evaluating these summary data on a regular (weekly) basis, the intervention team will determine whether a woman should be contacted, initially by the IVR system, which will have tailored messages to address anticipated issues. If the next report shows no improvement, one of the intervention staff, trained in motivational interviewing, will contact the participant and problem-solve methods for re-engaging with the IVR system, or, if that fails, set up an intervention staff-based telephone advice program (while continuing to work with the participant to re-engage the IVR system over time, as appropriate).

6.3 Safety and Adverse Events

All *WHISH*-eligible participants will receive safety tips for physical activity in their introductory packets adopted from the NIA/NIH *Go4Life®* materials[3] and the 2008 Physical Activity Guidelines for Americans from the DHHS.[13]

For both *WHISH* intervention-consented and control participants, outcomes will be collected through the usual standardized WHI procedures via Medical History Update (Form 33).

For *WHISH* IVR PA intervention-consented participants, program materials and the IVR will include a toll-free telephone number help line that participants will be instructed to call if they experience an adverse event that may result in the need to stop or temporarily stop their intervention participation and that may need to be reported as an adverse event.

For *WHISH* IVR PA intervention-consented participants The IVR system first inquires if the participant has experienced chest pains or difficulty breathing. If the participant endorses these symptoms, the IVR directs them to discontinue physical activity and consult urgently with their health care provider, and the system pushes a safety notification to the WHISH IVR administrator.

6.3.1. Safety Tips for all *WHISH*-Eligible Participants

Safety tips for physical activity adopted from the NIA/NIH *Go4Life®* materials and the 2008 Physical Activity Guidelines for Americans from the DHHS will be included with the introductory packets mailed to all *WHISH*-eligible participants. Tips include, but are not limited to, breathing regularly, using smooth steady movements, making slow steady movements, bending from the hips not the waist, stopping if pain is felt. Additionally, tips include slowly increasing intensity and duration, wearing proper shoes and clothing that fit the activity, and choosing safe environments.

6.3.2. Outcomes Collection for *WHISH* Intervention and Control Participants

For both *WHISH* PA intervention and control participants, outcomes will be collected through the usual standardized WHI procedures via Medical History Update (Form 33). The DSMB will monitor the outcomes reports for safety.

WHISH intervention participants who report an incident event contraindicating eligibility for *WHISH* during standard WHI outcomes collection will have their status reviewed to determine whether to continue, stop or stop temporarily participating in the intervention.

6.3.3. Adverse Event Reporting for the Intervention Group

WHISH intervention participants will be instructed to call the *WHISH* toll-free telephone help line to report any of the following *WHISH* outcomes that may occur:

- MI
- Stroke
- revascularization (CABG & PCI)
- hip fracture
- fracture of any kind other than hip
- falls that result in injury

Staff will follow standardized IRB and Human Subjects guidelines for determining whether the event is an adverse event (AE), serious adverse event (SAE), or unanticipated problem (UP) and follow national and institutional reporting requirements. Determination will be made whether the participant should continue with the *WHISH* intervention or stop, either permanently or temporarily by reviewing *WHISH* eligibility criteria of inability to walk or being within 6 months of a CVD event listed above and also by the healed status of any incident fracture. Regardless of intervention status (continue, stop, stop temporarily), participants will continue to be followed for data and outcomes by the standard WHI procedures.

6.4 Data Collection

Monitoring physical activity in the intervention and usual activity control arms will be based on self-reported physical activity through the standardized WHI questionnaires and through periodic accelerometry in a random subsample of *WHISH* participants in both arms. An annual *WHISH* Questionnaire will be developed for the trial which will be a shortened version of the physical activity questionnaire completed for the OPACH ancillary study. The *WHISH* questionnaire will be mailed to women randomized in *WHISH* with their WHI *Form 33 Medical History Update* (outcomes) mailings.

Data collection specifically in the intervention group will additionally incorporate IVR and postcard mail-back logs.

6.4.1. Physical Activity in the intervention and control groups

6.4.1.i. Questionnaire

Form 321 - OPACH Physical Activity Questionnaire is a 14-page form that asks about walking; mild, moderate and vigorous recreational activities; indoor household activities, outdoor yard-related activities; self-perceived exertion; sitting, sleeping and sedentary time; weekday vs. weekend activities, falls; and use of assistive devices for walking.

6.4.1.ii. Accelerometry monitoring will be embedded in Dr. LaCroix's OPACH cohort.

In the OPACH women who are eligible and subsequently randomized in *WHISH*, the WHI DCC will select a random sample sufficient to enroll 1000 women from the *WHISH* PA intervention group and 1000 from the usual activity control group. In this group, the 2012/13 accelerometry data collection will serve as the pre-randomization baseline. In addition, these women already know how to use the accelerometer so the whole activity is streamlined. The WHI DCC will mail consent forms to this subgroup of women to participate in wearing the accelerometer (referred to a monitor for participants) for additional 1-week periods at 6, 18 and 36 months of follow-up.

The protocol for data collection, processing and reduction will be identical to Dr. LaCroix's current Objective Physical Activity for Cardiovascular Health (OPACH) study (see above.) At each time point, determined from their randomly assigned baseline date, this random sample will receive a GT3X accelerometer in the mail. Sleep logs will be provided so that time in bed can be subtracted from daytime activity patterns. Distribution (and return) of the accelerometer, instructions and sleep log will be implemented from the Exercise and Physical Activity Resource Center (EPARC) at the University of California, San Diego.

The instruction sheet will direct women to place the accelerometer on the right hip, above the iliac crest, using a belt worn around the waist. A phone number to the EPARC at the University of California, San Diego will be provided for problems or questions. The participants will wear the accelerometer for 7 days, during waking and sleeping hours except during bathing or swimming. A staff person from the EPARC at the University of California, San Diego, will telephone each participant prior to mailing to inform participants that the monitor is on its way and to answer any questions. After wearing it for 7 days, participants will mail the accelerometer and sleep log to the EPARC at the University of California, San Diego in a postage-paid return envelope. The accelerometer raw data will be downloaded, visually checked for evidence of wear time as quality control, and stored. Reminder calls will be made to women for those accelerometers that have not been returned within 2 weeks of the expected return date. A thank you letter and monetary incentive will be mailed to all participants who return their accelerometer.

Accelerometry data will be summarized as follows:

- Non-wear time will be defined as a period of 60 minutes or more of zeros, as recommended by NHANES[14] and.[15]
- Standard measurement day will be defined and multiple imputation will be used to account for missing accelerometer data.[16] As a sensitivity analysis, we will compare this approach to a complete case analysis using 10 hrs/day, as in NHANES.[14]
- Total volume of physical activity is assessed by mean counts per minute.
- Minutes of sedentary behavior, light-intensity and moderate-to vigorous intensity physical activity will be estimated by setting thresholds (or cutpoints) for counts/minute derived from the OPACH calibration study. We will also characterize the total steps/day taken and steps in each intensity category.
- Other characteristics of physical activity: Number of days per week and time spent in bouts of at least 10 minutes of MVPA, which are currently the only bouts of activity that count toward national PA guidelines, will also be assessed. Another variable of interest

is duration of sedentary time, as emerging research suggests this may be an independent risk factor of CVD.[17,18]

6.4.2. *WHISH* Intervention Self-Monitoring

6.4.2.i. IVR System for full program. During each IVR system phone contact, women will report information on PA type and amount, along with pedometer steps. To track their PA behavior between IVR calls, women will be provided with sets of calendars to record their daily activity, as a self-monitoring tool. (On-line versions and means to enter the data, and download the pedometers will also be available.) The IVR system is designed to reliably collect this information. It will encourage participants to take advantage of the tailored messages built into the IVR, including the ability to request contact with a staff person, through a toll-free 800 number.

The IVR system automatically summarizes the data in an electronic database that can be accessed by intervention staff. Upon evaluating these summary data on a regular (weekly) basis, the intervention team will determine whether a woman should be contacted, initially by the IVR system, which has the tailored messages to address anticipated issues. If the next report shows no improvement, one of the intervention staff, trained in motivational interviewing, will contact the participant and problem-solve methods for re-engaging with the IVR system, or, if that fails, set up an intervention staff-based telephone advice program (while continuing to work with the participant to re-engage the IVR system over time, as appropriate).

In addition to information about PA type, frequency, intensity, pedometer steps, and related participant experiences, the IVR provides a means of collecting information on injuries and health outcomes, and participant choices of specific behavioral messages heard in relation to barriers to exercise. Additional information can also be obtained as the trial progresses, i.e., the IVR programming can be modified. Similar types of information will be collected via the mail-back cards.

6.4.2.ii. Mail-backs for mailings (+website) only program. Participants will receive pre-paid mail-back postcards for submitting their physical activity logs.

7.0 Statistical Plan

Primary Analysis. The primary aim of the *WHISH* trial is to test the effect of a centralized PA intervention on CVD risk. Safety outcomes that will be monitored are hip fracture and death from non-CVD causes. Supplemental information will be provided on the number of falls, total fracture, coronary revascularization procedures, venous thromboembolic events (VTE) and incident peripheral artery disease (PAD). In particular, a secondary hypothesis is that PA intervention will reduce the rates of VTE and PAD. A final secondary hypothesis is that PA intervention will lead to less loss of physical function and a higher rate of maintaining mobility.

The main comparisons of intervention groups with respect to the distribution of time until the first post-randomization occurrence of a CVD event, as well as the other monitored outcomes, will use a Cox proportional hazards model. The model will be stratified for type of outcomes collection (MRC or medicare) and by age and region, as these strata are used for randomization. For the continuous outcomes of number of falls and loss of physical function (as measured by the Physical Function Score), we will use a linear regression model, weighted by the amount of observation time for each subject. Explicit pre-defined rules for censoring will be recorded in an analysis plan for approval by the DSMB. In these analyses, the "intention to treat" approach will be used in which women are grouped according to randomization assignment. That is, all women who are randomized will be part of the analysis, including those in the intervention arm that do not consent,

as is done in the randomized consent design.[19] Details about the sequential monitoring plan are given in Section 9.0 Study Monitoring.

Analysis of the Accelerometry Study data allows us to measure how well the subjects adhere to the treatment. Several analyses are of interest: we will compare the level of activity measured by the accelerometer at a particular time point between the intervention group (or the part of the intervention group that consented to the treatment) and the control group. We will also compare the difference in activity level during follow-up and at baseline between these groups. Another analysis that is of interest is to compare the results of the accelerometry study to the self-reported activity level. It will be possible to use this comparison to develop a regression calibration method, similar to [20-23]. In this model we would correct for intervention status, consent status, and relevant other descriptive data, such as BMI. This regression calibration model can then be used to generate an additional adherence corrected analyses of the trial.

Adherence corrected analyses. While this would *not* be an intent-to-treat analysis, based on which the DSMB would make decisions about the continuation of the trial, it is of interest to study what the treatment effect is among those women who adhere to the treatment. We plan to do this in two different ways.

1) The simplest way to get an adherence adjusted treatment is to only consider subjects assigned to the active intervention, who consented to the intervention. This is a very straightforward analysis. A minor modification of this analysis is to censor those subjects who communicate that they have stopped the intervention some amount of time (say 3-6 months) after the stopping. This analysis can both be carried out using all subjects in the intervention, and using subjects that consented to IVR. A disadvantage of these analyses is that the control group does not consent – thus all subjects in the control group would be used in such an analysis, that would therefore likely have systematic differences between the two groups that are compared.
2) An alternative adherence adjusted analysis is obtained by obtaining an unbiased estimate of the "true activity level", based on the calibration procedure described above, of s subject, and to use this as a covariate in the analysis of the outcomes data of the *WHISH* trial.

Power for Primary Analysis. We would consider a 10-15% effect size to be of substantial public health significance. For power calculations, the effect size is averaged for the women who consent to intervention, and those who do not consent. Thus, with, for example, a 75% consent rate, a 15% overall effect size means a 20% effect among the women who consent, and a 0% effect among the women who do not.

Table 3. Power of the WHISH trial for the primary CVD outcome, α=.05, two-sided.

	With 44 months follow up (early 2019)			With one additional year of follow-up		
	Expected number of CVD events			Expected number of CVD events		
Effect	Controls	Intervention	Power	Controls	Intervention	Power
10%	940	847	60.9%	1191	1073	71.1%
11%	940	838	69.4%	1191	1062	79.2%
12%	940	829	77.0%	1191	1050	85.8%
13%	940	820	83.4%	1191	1039	91.0%
14%	940	810	88.6%	1191	1027	94.5%
15%	940	801	92.5%	1191	1015	96.9%

In Table 3 we present the power calculations from our grant proposal. The grant proposal proposed active consent to the written materials, and thus lower consent rates, which would imply lower power than the current design. The power calculations will be updated in early 2016 with actual consent rates and updated cardiovascular event rates. From this table we see that we have about 80% power for finding a significant treatment effect if the true effect size is about 12.5% and

90% if the true effect size is about 14.5%. If the trial were extended one year, for example using a no cost extension, we have about 80% power for finding a significant treatment effect if the true effect size is about 11% and 90% if the effect size is a little under 13%. This is an effect size that has substantial public health significance, and can realistically be expected based on prior data.

8.0 Data Management

The *WHISH* PA intervention data will be managed by Stanford Biostatistician David Ahn, Ph.D. who has considerable experience with data collected by the IVR data system. He will work closely with the Stanford investigators and intervention team to ensure timely communications regarding adherence to both IVR contacts and the Intervention PA goals, particularly with respect to endurance activity, but also muscle-strengthening, balance/flexibility exercises and sedentary behavior. Relevant adherence data will be transmitted by the Stanford CCC to the DCC for incorporating in the main WHI Study Database.

The IVR data will be stored on a secure data server housed at the Stanford Arastradero Rd, Palo Alto, CA. offices. The facility has a dedicated server room with controlled access. Servers are encrypted and the web-based application to interface with the server is SSL encrypted. The server connects to a web-based interface on the project assistant's workstation; staff access the server through SSL encrypted log-in to remotely monitor and analyze data. Telephone advisors track their data in a secured Filemaker Pro database created specifically for the project. This database is also stored in an encrypted and secured server with controlled staff access. Autorecorded phone sessions will be stored as electronic files on the secured remote server. Project staff will download the data periodically from the IVR and staff Filemaker databases for the Stanford-based biostatistician (*WHISH* Data Manager) who will analyze and track intervention usage and physical activity patterns. The IVR will have customized reports accessible on the interface to medical alerts collected by the IVR (i.e., if a participant endorses inability to exercise due to physical or medical issues) or non-response/non-engagement in IVR calls. These reports will notify staff when follow-up calls or safety checks are needed.

The *WHISH* outcomes data will ne managed by the WHI CCC database unit, jointly with the regular WHI data. The WHI database will also keep track of randomization, consent, and intervention, as well as forms that are send to all *WHISH* participants.

The accelerometry data will be preprocessed at UC San Diego, and then transferred to the *WHISH* DCC (WHI CCC). Work flows for this data are being developed in close consultation with the OPACH investigators who have experience analyzing accelerometry data, and are located at the same institution as the *WHISH* DCC.

9.0 Study Monitoring

The trial will be monitored by an NHLBI appointed, independent Data Safety and Monitoring Board (DSMB). Shortly after the start of the study, the DSMB will approve the study protocol, consent forms, and monitoring plan. At the end of the first year it will approve the analysis plan, and monitor recruitment and consenting process, and during subsequent years it will monitor study outcomes. These monitoring meetings will happen annually between 2015 and 2019. The primary outcome for the trial is incident CVD (MI, stroke, and CVD death); safety outcomes include death from non-CVD causes and hip fracture. Supplemental data on revascularizations, falls, and total fracture will also be provided, as well as adherence adjusted outcomes reports and adherence summaries as described in Section 7.

Trial monitoring will use the Lan-DeMets spending function with O'Brien-Fleming type boundaries corrected for the irregular review times.[24] For the primary outcome a 5% two-sided significance level will be used; for harm a 10% two-sided significance level will be used, divided equally

between incident CVD, death from non-CVD causes, and hip fracture. The stopping boundaries corresponding to these rules, based on the expected accumulation rate of outcomes, can be computed using standard software.

Additional details of the monitoring plan are provided in a separate monitoring document to be developed jointly with the DSMB.

10.0 Study Administration and Organization

WHI Data Coordinating Center:

Principal Investigator: Andrea Z. LaCroix, PhD, is Professor of Epidemiology at the University of California at San Diego, and a senior women's health epidemiologist and clinical trialist with 30 years of experience. She is currently multiple-PI of the DCC for the Menopause Strategies: Finding Lasting Answers for Symptoms and Health (MsFLASH) Data Coordinating Center based at the Fred Hutchinson Cancer Research Center (FHCRC) alongside the WHI Clinical Coordinating Center (WHI-CCC). MsFLASH is an NIA-funded U01 with 5 clinical sites conducting multiple RCTs designed to test new interventions for managing menopausal symptoms in midlife women including a physical activity trial. She has been with the WHI since its inception in 1992 and joined the WHI-CCC as a Co-PI/Co-Project Director in 1996 performing multiple leadership functions over the past 17 years. Dr. LaCroix's WHI responsibilities currently include supporting collaborative science within the WHI Program devoted to aging and cardiovascular disease, serving as Chair of the Laboratory Working Group, and serving on the Steering Committee and Publications and Presentations Committee.

Principal Investigator: Charles L Kooperberg, PhD, is Program Head of the Biostatistics Program and Full Member at the FHCRC and the WHI-CCC. Within the WHI-CCC, Dr. Kooperberg was actively involved in the semi-annual reporting of the WHI Clinical Trials: he was responsible for several chapters of the semi-annual report to the Data Safety and Monitoring Board (DSMB), and attended all DSMB meetings. This gives him ample experience in monitoring complex clinical trials and interactions with DSMBs. Within the WHI-CCC, he is the scientific contact to the Outcomes Adjudication Committee. As such, he is intimately familiar with all details of collection of clinical outcomes, which will be of invaluable importance for the *WHISH* trial. Dr. Kooperberg's responsibilities include the annual reporting of primary and safety outcomes to the DSMB and oversight of the incorporation of medicare and NDI data with WHI outcomes data.

The WHI Clinical Coordinating Center: The Women's Health Initiative (WHI) is a large and comprehensive set of long-term studies of women's health. The WHI Clinical Trial (CT) includes three randomized, controlled trials which tested the overall benefit and risk of promising but unproven prevention strategies for some of the most common causes of morbidity and mortality in postmenopausal women: 1) hormone therapy (HT) for coronary heart disease prevention, with breast cancer and hip and other fractures as secondary outcomes; 2) dietary modification (DM) involving fat reduction, with increased vegetable, fruit, and grain consumption, for breast and colon cancer, and secondarily, heart disease, prevention; and 3) calcium and vitamin D supplementation (CaD) to reduce the risk of hip fracture, and secondarily, all fractures and colorectal cancer. The WHI Observational Study (OS) was established to explore the predictors of morbidity and mortality in older women. The combined WHI CT and OS cohorts comprise an ethnically diverse study population of 161,808 U.S. postmenopausal women in the age range of 50-79. The history, rationale, and design of WHI have been published. The website www.whi.org contains a concise, but detailed description of the program, data collection forms, tabulations, publications, and ongoing and completed ancillary studies. In 2005, close-out visits occurred for women enrolled in the various components of the WHI program. An extended follow-up phase was begun to collect outcome events through 2015 and beyond among consenting women. The WHI CCC just celebrated its 20th anniversary. We retain a core staff of clinical and technical professionals who have worked together for years, many since the inception of the program.

Stanford Clinical Coordinating Center:

Principal Investigator: Marcia Stefanick, PhD, is Professor of Medicine (Stanford Prevention Research Center, SPRC) and of Obstetrics & Gynecology, Stanford University, Director, Stanford Women and Sex Differences in Medicine (WSDM) Center, and PI of the Western Regional Center of the WHI Extension Study (2010-present). She was PI of the Stanford WHI Clinical Center for the Clinical Trial (CT) and Observational Study (OS) from the1994 onset to 2010, and the WHI Steering Committee (SC)-elected Chair of the WHI SC and Executive Committee from 1998 to 2011. As PI of two large multi-center trials involving long-term lifestyle change, the 12-year WHI Diet Modification (DM) trial and 10-year NCI-funded Women's Healthy Eating and Living (WHEL) plant-based diet intervention trial of early stage breast cancer survivors, she has considerable experience with behavioral strategies to optimize adherence to lifestyle change. She also has expertise in physical activity intervention trials with CV risk outcomes, having directed a series of NHLBI-funded 1-to-2 year group- and home-based randomized, controlled PA trials, including the Stanford Weight Control Projects[25,26] and the Diet and Exercise for Elevated (Cardiovascular) Risk (DEER) trial[27] involving collectively about 1000 men and women of various age, BMI, and CV risk groups. She is also the Stanford PI on Dr. LaCroix's WHI OPACH study, for which Stanford is conducting a calibration study to derive accelerometry cut points for light vs. moderate/vigorous activity for the WHI cohort.

Stanford University, Western Regional Center of the current WHI Extension Study (PI: Marcia Stefanick, PhD)

The Stanford Western Regional Center is responsible for outcomes and data collection of ~22,000 (~24%) of the 93,550 WHI participants who consented, through the WHI Clinical Coordinating Center (Fred Hutchinson Cancer Research Center, FHCRC, the *WHISH* DCC), to ongoing follow-up from 2010 onward, i.e., with no need for future re-consenting. The highly trained and experienced WRC outcomes staff will take on any additional activities required to obtain medical records for *WHISH* participants whose records are no longer available through medicare linkage. Co-investigator, **Mark Hlatky, M.D.,** Professor of Health Research & Policy (Health Services Research) and of Medicine (Cardiovascular Medicine), and Stanford's Physician Adjudicator during the WHI CT/OS phase, has played a key role in demonstrating the validity of the CV outcome data, particularly MI, provided by the medicare linkage. Dr. Hlatky will continue to collaborate with DCC investigators on optimal utilization of administrative data in this pragmatic trial.

Delivering and Managing the *WHISH* PA intervention program: Abby King, PhD, Behavioral Science Director of the proposed *WHISH* PA Intervention, Professor of Health Research & Policy (Epidemiology) and of Medicine (SPRC), is an internationally recognized expert in the area of community-based PA promotion in older adults. The overarching goal of much of her research has been to broaden the reach and translation of theoretically supported and evidence-based health promotion interventions to underserved and at-risk populations across the U.S. and abroad. As part of this research line, her lab has evaluated the effectiveness of a range of potentially lower cost, mediated communication channels for delivering empirically supported interventions to foster sustained health behavior change, including by mail, telephone, trained volunteers, and automated computer advising and support systems, including IVR systems and portable devices, all of which are built into the WHI PA Intervention. In collaboration with her nationally recognized colleague, **Cynthia Castro, PhD,** who will provide ongoing oversight of the IVR system.

Statistical Analysis: Stanford biostatisticians, **Phillip Lavori, PhD**, Professor and Chair of the Department of Health Research & Policy, Director of Biostatistics, Epidemiology, and Study Design Program in Spectrum (Stanford's NIH CTSA), and other major Stanford Medical School programs, and **Michael Baiocchi, PhD,** Assistant Professor of Medicine (SPRC) will interface with the DCC PI, Charles Kooperberg, PhD, and his FHCRC colleagues, all of whom have considerable experience with behavioral trial data in a setting of comparable size and scope as the proposed

WHISH trial. Drs. Lavori and Baiocchi will be blinded from outcomes data that the DCC will prepare for monitoring reports; they will, however, provide the DCC with PA intervention data for these reports and work with DCC statisticians on analyses of PA data from questionnaires and the *WHISH*-OPACH accelerometry substudy. In addition to monitoring reports, the statistical teams will work together on analyses for baseline and final manuscripts.

11.0 Study Timeline

	Yr 1 M1-3	Yr 1 M4-6	Yr 1 M7-9	Yr 1 M10-12	Yr 2	Yr 3	Yr 4	Yr 5 M1-9	Yr 5 M10+
Finalize IVR and Intervention Materials	X								
IRB approvals (FHCRC, Stanford)	X								
Screening, Randomization, Consenting (DCC)	X	X	X	X					
Initiate Intervention in Vanguard cohort		X	X						
Intervention: Adoption Phase (3 months)			X	X					
Intervention Optimization and Maintenance			X	X	X	X	X	X	
Study closeout/Analyses/publication of final manuscripts									X

12.0 Publication Plan

Analyses and publication will occur during year 5 of the *WHISH* trial. WHI publication and presentation policies will be followed. *WHISH* Principal Investigators will provide a suggested list of publications, inviting input from WHI co-investigators. As an ancillary study to the WHI, all *WHISH* data will be made available to WHI investigators per WHI ancillary study policy.

13.0 Attachments

WHI consents, forms, policies, ancillary studies and procedures referred to in the *WHISH* protocol may be accessed at www.whi.org

- WHI Extension Consent
- WHI Ancillary Study Objective Physical Activity for Cardiovascular Health (OPACH, Ancillary Study 286; LaCroix, PI)
- *Form 33 - WHI Medical History Update*
- *Form 155 - WHI Lifestyle*
- *Form 321 - WHI OPACH Physical Activity Questionnaire*
- WHI Outcomes Collection
- WHI Publications and Presentations Policy
- WHI Ancillary Study Policy

14.0 References

1. Physical Activity Guidelines Advisory Committee. Physical Activity Guidelines Advisory Committee Report, 2008. Washington, DC: U.S.: Department of Health and Human Services; 2008:683.

2. Lauer MS. How will exercise capacity gain enough respect? *Circulation.* Apr 5 2011;123(13):1364-13663. *Go4Life*®. 2013; http://go4life.nia.nih.gov/. Accessed May 30, 2013, 2013.

4. Manson JE, Greenland P, LaCroix AZ, Stefanick ML, et al. Walking compared with vigorous exercise for the prevention of cardiovascular events in women. *N Engl J Med* 2002;347(10):716-725.

5. Chomistek AK, Manson JE, Stefanick ML, Lu B, et al. The Relationship of Sedentary Behavior and Physical Activity to Incident Cardiovascular Disease: Results from the Women's Health Initiative. *J Am Coll Cardiol.* Apr 10 2013.

6. Pescatello LS, Franklin BA, Fagard R, Farquhar WB, et al. American College of Sports Medicine position stand. Exercise and hypertension. *Med Sci Sports Exerc* 2004;36(3):533-553.

7. American Geriatrics Society. Exercise prescription for older adults with osteoarthritis pain: consensus practice recommendations. A supplement to the AGS Clinical Practice Guidelines on the management of chronic pain in older adults. *J Am Geriatr Soc* 2001;49(6):808-823.

8. Colcombe S, Kramer AF. Fitness effects on the cognitive function of older adults: a meta-analytic study. *Psychol Sci* 2003;14(2):125-130.

9. Curb JD, Mctiernan A, Heckbert SR, Kooperberg C, et al. Outcomes ascertainment and adjudication methods in the women's health initiative. *Ann Epidemiol* 2003;13(9, Supplement 1):S122-S128.

10. King AC, Castro CM, Buman MP, Hekler EB, et al. Behavioral Impacts of Sequentially versus Simultaneously Delivered Dietary Plus Physical Activity Interventions: the CALM Trial. *Ann Behavioral Medicine: a publication of the Society of Behavioral Medicine.* Apr 23 2013.

11. King AC, Hekler EB, Castro CM, Buman MP, et al. Exercise Advice by Humans Versus Computers: Maintenance Effects at 18 Months. *Health Psychol: Official Journal of the Division of Health Psychology, American Psychological Association.* Feb 18 2013.

12. King AC, Friedman R, Marcus B, Castro C, et al. Ongoing physical activity advice by humans versus computers: the Community Health Advice by Telephone (CHAT) trial. *Health Psychology: Official Journal of the Division of Health Psychology, American Psychological Association* 2007;26(6):718-727.

13. 2008 Physical Activity Guidelines for Americans. www.health.gov/paguidelines. Accessed May 30, 2013, 2013.

14. Troiano RP, Berrigan D, Dodd KW, Masse LC, et al. Physical activity in the United States measured by accelerometer. *Med Sci Sports Exerc.* Jan 2008;40(1):181-188.

15. Evenson KR, Terry JW, Jr. Assessment of differing definitions of accelerometer nonwear time. *Research Quarterly for Exercise and Sport* 2009;80(2):355-362.

16. Catellier DJ, Hannan PJ, Murray DM, Addy CL, et al. Imputation of missing data when measuring physical activity by accelerometry. *Med Sci Sports Exerc* 2005;37(11 Suppl):S555-562.

17. Healy GN, Owen N. Sedentary behaviour and biomarkers of cardiometabolic health risk in adolescents: an emerging scientific and public health issue. *Revista Espanola de Cardiologia* 2010;63(3):261-264.

18. Healy GN, Dunstan DW, Salmon J, Cerin E, et al. Breaks in sedentary time: beneficial associations with metabolic risk. *Diabetes Care* 2008;31(4):661-666.

19. Zelen M. A new design for randomized clinical trials. *N Engl J Med* 1979;300(22):1242-1245.

20. Tinker LF, Sarto GE, Howard BV, Huang Y, et al. Biomarker-calibrated dietary energy and protein intake associations with diabetes risk among postmenopausal women from the Women's Health Initiative. *Am J Clin Nutr* 2011;94(6):1600-1606.

21. Prentice RL, Mossavar-Rahmani Y, Huang Y, Van Horn L, et al. Evaluation and comparison of food records, recalls, and frequencies for energy and protein assessment by using recovery biomarkers. *Am J Epidemiol* 2011;174(5):591-603.

22. Prentice RL, Huang Y, Tinker LF, Beresford SA, et al. Statistical Aspects of the Use of Biomarkers in Nutritional Epidemiology Research. *Stat Biosci* 2009;1(1):112-123.

23. Neuhouser ML, Tinker L, Shaw PA, Schoeller D, et al. Use of recovery biomarkers to calibrate nutrient consumption self-reports in the Women's Health Initiative. *Am J Epidemiol* 2008;167(10):1247-1259.

24. O'Brien PC, Fleming TR. A multiple testing procedure for clinical trials. *Biometrics* 1979;35(3):549-556.

25. Wood PD, Stefanick ML, Dreon DM, Frey-Hewitt B, et al. Changes in plasma lipids and lipoproteins in overweight men during weight loss through dieting as compared with exercise. *N Engl J Med* 1988;319(18):1173-1179.

26. Wood PD, Stefanick ML, Williams PT, Haskell WL. The effects on plasma lipoproteins of a prudent weight-reducing diet, with or without exercise, in overweight men and women. *N Engl J Med* 1991;325(7):461-466.

27. Stefanick ML, Mackey S, Sheehan M, Ellsworth N, et al. Effects of diet and exercise in men and postmenopausal women with low levels of HDL cholesterol and high levels of LDL cholesterol. *N Engl J Med* 1998;339(1):12-20.

Phase 2: 1R61HL151885-01 (9/15/20-8/31/21); R33HL151885 (subsequent years)

	**Table of Contents**	
Section	Topic	Page
1.	Basic Information	2
2.	Study Population Characteristics	2
2.1.	Conditions or Focus of Study	2
2.2.	Eligibility at Randomization	2
2.3.	Age Limits	3
2.4.	Inclusion of Women, Minorities, and Children	3
2.5.	Recruitment and Retention	4
2.6.	Recruitment Status (Active, not recruiting)	6
2.7.	Study Timeline	6
2.8.	Enrollment of First Participant	6
2.9.	Inclusion Enrollment Reports	6
3.	Protection and Monitoring Plans	8
3.1.	Protection of Human Subjects	8
3.1.1.	Risks to Human Subjects	8
3.1.1.a.	Human Subjects Involvement, Characteristics, Design, and Specific Aims	8
3.1.1.b.	Study Procedures, Materials, and Potential Risks	10
3.1.2.	Adequacy of Protection Against Risks	15
3.1.2.a.	Informed Consent and Assent	15
3.1.2.b.	Protection Against Risks	15
3.1.2.c.	Vulnerable Subjects	16
3.1.3.	Potential Benefits of the Proposed Research Participants and Others	16
3.1.4.	Importance of Knowledge to be Gained	16
3.2.	Multisite Study with same protocol at >1 domestic site? No	16
3.3.	Data and Safety Monitoring Plan	17
3.3.1.	Protocol Defined Outcomes	17
3.3.2.	Monitoring Guidelines	17
3.3.3.	Interim Analysis for the WHISH Trial to be taking place in 2022.	19
3.3.4.	Reporting of Primary, Safety and Secondary Outcomes	20
3.3.5.	Reporting of Physical Activity Levels	21
3.3.6.	Reporting of Intervention Activities	21
3.3.7.	Reporting of Additional Outcomes	22
3.3.8.	Timeline for Outcomes Data Collection and Availability	22
3.3.9.	Outcome Definitions	23
3.4.	Data and Safety Monitoring Board	24
3.5.	Overall Structure of the Study Team	24
4.	Protocol Synopsis	26
4.1.	Study Design	26
4.2.	Outcome Measures (See sections 3.3.1. and 4.3)	26
4.3.	Statistical Design and Power	26
5.	Other Clinical Trial-related Information	29
5.1.	Project Management Plan	29
5.2.	Single Site Justification Plan	29
5.3.	Dissemination Plan	30
6.	Clinical Trial Milestone Plan	31
7.	References (Phase 2)	32

SECTION 1 - BASIC INFORMATION

1.1. Study Title: Women's Health Initiative Strong and Healthy Study
1.2. Is this study exempt from Federal Regulations: No
1.3. Exemption Number: NA
1.4. Clinical Trial Questionnaire
   1.4.1. Does this study involve human participants? Yes
   1.4.2. Are the participants prospectively assigned to an intervention? Yes
   1.4.3. Is the study designed to evaluate the effect of the intervention on the participants? Yes
   1.4.4. Is the effect that will be evaluated a health-related biomedical or behavioral outcome? Yes
1.5. Provide the ClinicalTrials.gov identifier: NCT02425345

SECTION 2 - STUDY POPULATION CHARACTERISTICS

2.1. Conditions or Focus of Study

- Myocardial Infarction
- Stroke
- Cardiovascular Mortality
- Hip Fractures
- Falls
- Mortality
- Venous Thromboembolic Event
- Peripheral Artery Disease
- Physical Function
- Coronary Revascularization
- Mobility and Independence
- Clinical (Non-hip) Fracture

Phase 2 adds to the above:

- Short Physical Performance Battery (SPPB) in subset
- Sleep Disturbance
- Depressive Symptoms

2.2. Eligibility Criteria (at randomization)

Inclusion Criteria

- Currently enrolled in the Women's Health Initiative (WHI) Extension Study
- Known to be alive
- Cardiovascular outcomes will be available (enrolled in the WHI Medical Records Cohort or linked to Medicare Data)

Exclusion Criteria

- Inability to walk
- Dementia
- Residing in a nursing home

2.3. Age Limits (at randomization): Min Age: 66 Years Max Age: 99 years

2.4. Inclusion of Women, Minorities, and Children

Women and Minorities

All participants in the study are women because the *WHISH* trial is embedded in the large, ongoing Women's Health Initiative (WHI) Extension Study. WHI is a study of postmenopausal women designed by the NIH in 1991 to address the critical lack of evidence on postmenopausal women's health when the WHI Program was started in 1993. No men were included in the study, because the specific focus was on addressing interventions (Hormone Therapy, Dietary Modification, Calcium-Vitamin D supplementation) and health outcomes (coronary heart disease (CHD), breast cancer, and hip fractures), of special concern to women. The *WHISH* trial began in 2015 and this application proposes a continuation to extend follow-up to 9 years so that the *WHISH* trial can reach a definitive conclusion. Moreover, older women in the age range of *WHISH* have higher rates of new and recurrent cardiovascular events than men and no previous trials have investigated preventive interventions for cardiovascular disease in women.

The original recruitment to WHI included several strategies for recruiting minorities, e.g., 10 "Minority Clinical Centers" selected by the NIH with the specific goals of recruiting minority women, to ensure that minorities were adequately represented in all WHI component studies. The sampling frame for this study includes substantial minority representation as shown in the Inclusion Enrollment Table. No minority groups are excluded. The *WHISH* trial is 100% women, including 15.7% minorities (9.2% Black or African American, 3.3% Hispanic or Latina, 1.9% Asian or Pacific Islander and 0.3% American Indian or Alaska Native).

NIH-Defined Phase III Clinical Trial, Ensuring Valid Analysis

The *WHISH* randomized trial assigned intervention/comparison arm by random allocation of 50% intervention and 50% comparison stratified by race/ethnicity, age at randomization, data source (WHI Medical Records Cohort with outcomes adjudication or Medicare), and region (West, Midwest, Northeast, and Southeast). This ensures a valid, un-biased analysis.

Reporting on and Analyzing Differences in Race and Ethnicity

The WHI has been maintaining, collecting and presenting data on race and ethnicity as required by the Office of Management and Budget since it was initiated in 1993, including Hispanic, American Indian or Alaska Native, Asian, Black or African American, Native Hawaiian or other Pacific Islander, and White.

Prior evidence does not support race/ethnicity differences in the effects of physical activity on cardiovascular disease or associations between physical activity and cardiovascular risk factors. Nonetheless, although we do not have sufficient power to test for treatment effects within the individual groups, we will conduct stratified analyses and test for interaction to explore whether there are differences.

Inclusion of Children

The *WHISH* trial is embedded in the WHI Extension Study which is continuing to follow women who were enrolled in the NIH Women's Health Initiative at ages 50-79 at baseline (1993-1998), and therefore had only adult postmenopausal women participants. The NIH recognized that by focusing on postmenopausal women children would be excluded from the WHI. *WHISH* participants were aged 66-99 at randomization in 2015 (mean age ~80 years).

2.5. Recruitment and Retention Plan

Recruitment

WHISH is a pragmatic trial with a randomized consent design, embedded in the Women's Health Initiative (WHI) Extension Study (2010-2020), for which 49,331 women (9% African American), aged 66-99 years were randomly assigned to a physical activity (PA) intervention or usual activity comparison.

Schema:

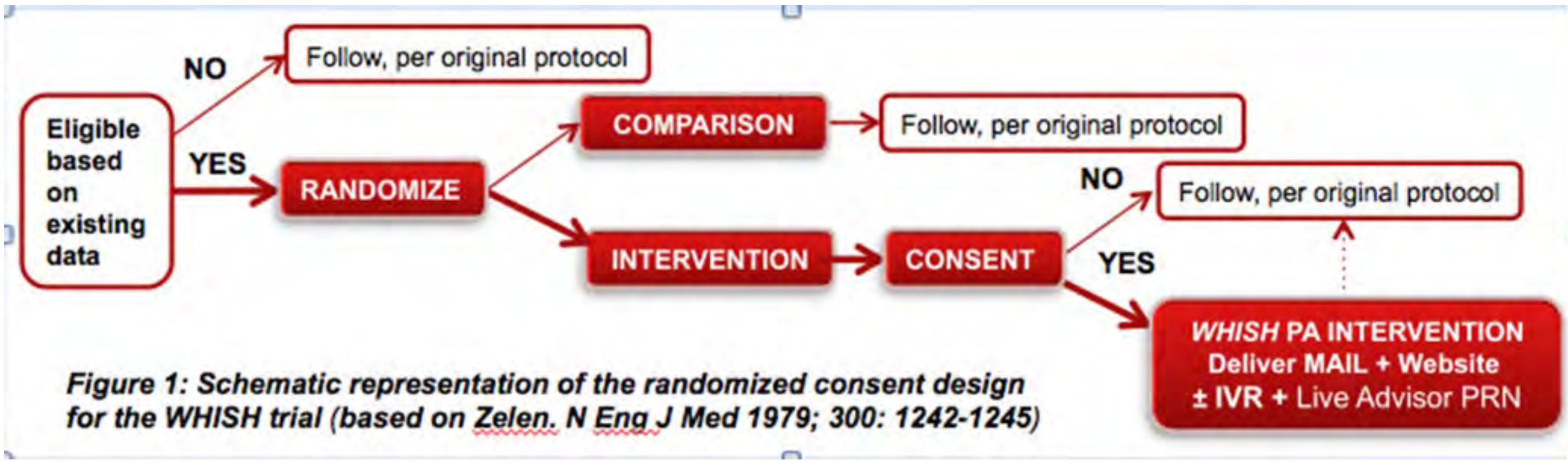

Figure 1: Schematic representation of the randomized consent design for the WHISH trial (based on Zelen. N Eng J Med 1979; 300: 1242-1245)

WHISH utilized a randomized consent design as originally proposed by Zelen (1979). Through this design, women were pre-screened for eligibility based on previously collected WHI data (see section 2.2. Eligibility Criteria). WHI newsletters were sent to all eligible WHI participants in advance of implementing *WHISH* to inform them that a new physical activity study was being started and that they may be invited to participate. After pre-screening, but prior to the first *WHISH* contact, 49,331 WHI participants were randomized.

<u>Women randomized to intervention</u> were sent a welcome packet and asked to call the WHI Clinical Coordinating Center if they did not want further contact from the *WHISH* intervention Center at Stanford. After removing women who "opted out" of this **<u>passive consent process</u>**, did not have a deliverable address or who had died after randomization but before intervention mailings, the intervention began to be delivered to 23,653 women. See Schema above and *WHISH* CONSORT Diagram below.

WHISH CONSORT Diagram Data
as of July 20, 2020

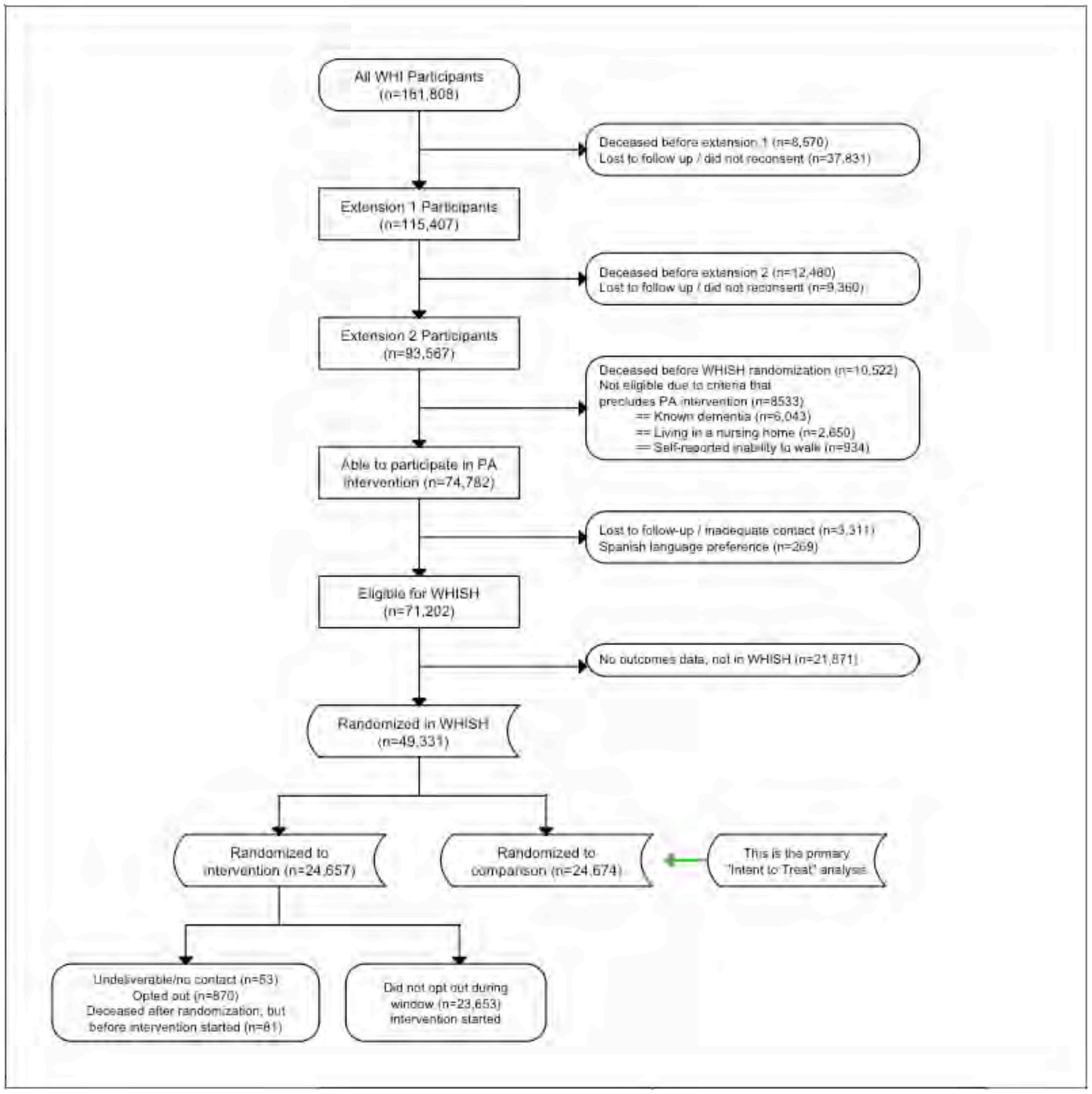

During Phase 1 of the *WHISH* trial, the Accelerometry Substudy consented 2,350 participants (1,182 comparison; 1,168 intervention) who had participated in the WHI Objective Physical Activity for Cardiovascular Health (OPACH) study during which they wore a waist accelerometer for 7 days and nights and completed a sleep diary for each of the 7 nights. During phase 1 of *WHISH*, they consented to wear a waist accelerometer 3 times for 7 nights each over the course of 4 years and complete a sleep diary for each of the 7 nights. Participants who completed round 3 (of 3 rounds) of the *WHISH* Accelerometry Substudy (n~1400) will be invited to re-consent to an Accelerometry Substudy in the continuation of the trial, phase 2. To fill to n=2,000 participants, additional *WHISH* participants will be invited to consent for the first time. To reach n=2,000, we will recruit 500 women each quarter during year 7. Assuming a 40% consent and re-consent rate, we plan on mailing to n~5000 participants. There will be an ample number of eligible *WHISH* participants from which to draw for diverse age and race/ethnicity enrollment when recruiting for the next Accelerometry Substudy. The invitational consent packet will include a cover letter from the *WHISH* PIs and an informed consent form. The cover letter will provide a toll-free telephone number that participants may call and leave a message if they have questions about the study or the consent. The message line is monitored daily with calls logged. Staff will return calls to participants within 1-2 days. We have used this system successfully with the initial Accelerometry Substudy consent and several other WHI ancillary studies involving participant informed

consent that is not conducted in-person. The same staff and investigators who were responsible for recruiting and consenting participants for the initial Accelerometry Substudy again will be responsible for recruitment and consenting for the proposed Accelerometry Substudy.

Regarding competition with other clinical trials, over the years many WHI participants have participated in more than one WHI ancillary study. Considering the impact of *WHISH* participation on the main WHI, we use the annual collection rate of the WHI medical history update (outcomes) form as a metric. For WHI overall, the outcomes form response rate between 6/1/2018 and 11/30/2018 of currently active WHI participants not in any ancillary studies was 67.7%. For those in WHI with *WHISH* as the only ancillary study, response rate was 66.2% among *WHISH*-intervention and 70.8% in *WHISH*-comparison. These are in-progress numbers and do not represent final response rates. The WHI participants have been highly committed to the WHI since recruitment began in 1993 with 161,808 postmenopausal women between the ages of 50-79. Up to 26 years later, 66,548 WHI participants remain actively participating (returning outcomes forms) and span an age range of 66-102.

Retention

Retention starts with the WHI. Retention activities in the WHI Extension Study consist of collecting the annual data forms and tracking hard to find and lost participants. Strategies and procedures to ensure a participant's retention and identification with WHI must be used throughout the course of the study, from the consent to the last follow-up contact. Participants receive WHI newsletter annually that includes WHI updates, participant stories, and healthy living tips. In February 2015, all active WHI participants (n=84,658 of 93,500 who had consented to the WHI Extension 2010 forward) received a WHI pendant commemorating WHI's 20th anniversary.

Retention for the *WHISH* intervention is monitored by the Stanford staff to ensure that women who have requested no further intervention are removed from contact lists and receive no further mailings. As of July 20, 2020, 85.3% of alive participants, i.e., not known to be deceased, were receiving the intervention; 12.6% had requested that mailings be stopped; 2.1% had undeliverable addresses. Participant engagement, as assessed by return of annual surveys sent only to Intervention participants, has steadily increased from a low of 48% response rate to the first survey to 72% response rate for the fourth (most recent) survey. Intervention materials are developed with evidence-based behavioral strategies, such as adhering to the social cognitive model of health behavior acquisition and maintenance. The materials have been steadily adapted to reflect participants' interests, based on their input on surveys and feedback from postcards, phone calls, emails, and mail. The materials and now include "targeted" inserts for three groups of women, based on self-reported physical function and physical activity levels reported on WHI forms. *WHISH* intervention staff at Stanford are available by phone, post mail or email to respond to inquiries from individual participants. Refer to the Research Plan for additional details.

Retention for the Accelerometry Substudy is supported by staff at the implementation site at the University of California, San Diego, Exercise and Physical Activity Resource Center (EPARC). This staff call each participant to review the instructions with them before they receive the accelerometer and study materials, as well as during the study. Time is offered to discuss questions. Each participant receives the toll-free telephone number to call if she has questions during the study.

2.6. Recruitment Status: Active, not recruiting

2.7 Study Timeline

Activity	Yr 6	Yr 7	Yr 8	Yr 9
Targeted intervention	X	X	X	X

Behavior change assessments: Form 521	X	X	X	X
Outcomes ascertainment	X	X	X	X
WHISH Accelerometry Substudy		X	X	
WHISH/WHI Long Life Study home exams		X	X	
Study close-out, publication of primary trial reports				X
Plan and conduct biomarker pilot studies			X	X

2.8. Enrollment of First Participant (See Section 6.3.)

2.9. Inclusion Enrollment Reports

IER ID#	Enrollment Location Type	Enrollment Location
IER 289037	Domestic	

Inclusion Enrollment Report

1. Inclusion Enrollment Report Title: Physical Activity Intervention to Improve Cardiovascular Health in Older Women

2. Using an Existing Database or Resource? No

3. Enrollment Location Type: Domestic

4. Enrollment Country(ies): USA: United States

5. Enrollment Locations: WHI

6. Comments: Cumulative data are for WHISH participants randomized April-July 2015. The planned enrollment table shows the number of WHISH participants who were alive as of the 2019 DSMB report. No further enrollment will occur in the larger cohort of WHISH, although there is planned enrollment for the accelerometry substudy. At the time when race/ethnicity data were obtained for the WHI, 1993-1998, Asian or Pacific Islander was asked under a single question; Hispanic ethnicity was asked as a single category without racial differentiation; unknown was asked whereas more than one race was not asked. Hispanic racial category is reported collectively as unknown.

Planned

Racial Categories	Ethnic Categories				Total
	Not Hispanic or Latino		Hispanic or Latino		
	Female	Male	Female	Male	
American Indian/ Alaska Native	133	0	0	0	133
Asian	845	0	0	0	845
Native Hawaiian or Other Pacific Islander	0	0	0	0	0
Black or African American	3942	0	0	0	3942
White	35471	0	1472	0	36943
More than One Race	0	0	0	0	0
**Total**	40391	0	1472	0	41863

Cumulative (Actual)

Racial Categories	Ethnic Categories									Total
	Not Hispanic or Latino			Hispanic or Latino			Unknown/Not Reported Ethnicity			
	Female	Male	Unknown/ Not Reported	Female	Male	Unknown/ Not Reported	Female	Male	Unknown/ Not Reported	
American Indian/ Alaska Native	0	0	0	0	0	0	0	0	0	0
Asian	0	0	0	0	0	0	0	0	0	0
Native Hawaiian or Other Pacific Islander	0	0	0	0	0	0	0	0	0	0
Black or African American	0	0	0	0	0	0	0	0	0	0
White	0	0	0	0	0	0	0	0	0	0
More than One Race	0	0	0	0	0	0	0	0	0	0
Unknown or Not Reported	0	0	0	0	0	0	0	0	0	0
**Total**	0	0	0	0	0	0	0	0	0	0

SECTION 3 - PROTECTION AND MONITORING PLANS

3.1. Protection of Human Subjects

3.1.1. Risks to Human Subjects

3.1.1.a. Human Subjects Involvement, Characteristics, Design, and Specific Aims

The *WHISH* Trial is a randomized trial designed to answer a specific question regarding the efficacy of a physical activity (PA) intervention designed to promote levels of PA recommended by the U.S. Department of Human & Human Services for older Americans (https://health.gov/paguidelines/) compared to a usual activity comparison group. Specifically, the trial addresses whether a centralized public health intervention designed to increase and/or maintain aerobic (endurance) activity, primarily walking, combined with muscle strengthening exercises, and exercises to promote balance and flexibility, as well as encouragement to reduce sedentary behavior, primarily sitting, reduces major cardiovascular (CV) events. CV events are defined as myocardial infarction (MI), stroke (in particular, ischemic stroke), and CV death over an approximate 8-year follow-up period.

The specific aims of phase II of the *WHISH* trial are:

Primary Aim: Continue the centralized, adaptive, targeted PA intervention and ascertainment of adjudicated CVD, fracture and non-CVD death endpoints in WHISH women retaining their randomized treatment assignment to provide a minimum of 8 years of follow-up for surviving participants.

Primary hypothesis: The WHISH targeted PA intervention designed to increase aerobic PA combined with muscle strengthening, balance and flexibility exercises, and to reduce sedentary behavior, will reduce major CVD clinical events (composite of MI, stroke, and CV death), compared to the “usual follow-up group.

Secondary Aim: Determine WHISH intervention effects on measures of healthy CV aging including PF measured using the Short Physical Performance Battery (SPPB), sleep disturbances, and depressive symptoms. This Aim will use data collected in the LLS2 funded by the WHI Extension Study 2020-2027.

Hypothesis: The WHISH intervention will result in higher SPPB scores, improved sleep parameters, and lower levels of depressive symptoms compared to the usual activity group.

Exploratory Aim: Create an experimental framework within WHISH and conduct pilot studies to determine whether the WHISH PA intervention influenced levels of novel biomarkers indicative of healthy CV aging and/or occult myocardial injury, such as N-terminal pro-brain natriuretic peptide (NT-proBNP) and high sensitivity cardiac Troponin T (hsTnT). A WHISH Biomarker Advisory Group involving US experts will vet the most promising biomarkers for pilot testing. This aim will facilitate future WHISH/WHI Ancillary Studies and will utilize biospecimens collected in the LLS2 of the next WHI Extension Study, 2020-2027.

Hypothesis: WHISH Intervention women will have more favorable levels of CVD biomarkers.

Safety outcomes include non-cardiovascular death, hip and clinical (non-hip) fractures, falls, and revascularization.

WHISH is a multi-ethnic cohort of 49,331 women aged 66-99 (mean age ~80 years) at *WHISH* randomization April-July 2015 who were enrolled and actively participating in the NHLBI-funded Women’s Health Initiative (WHI) Extension Study, excluding those who by self-report were unable to walk, had a recent diagnosis (within past 6 months) of CV disease, dementia, and known to

reside in a nursing home. Among *WHISH* participants, 84% were white, 9% Black or African American, 3% Hispanic, 2% Asian or Pacific Islander, 0.3% Native American or Alaskan Native and 1% undeclared.

A total of 49, 331 participants were randomized to *WHISH* of which 24,674 (50%) were randomized to comparison and 24,657 (50%) to intervention. Of those randomized to intervention, 860 (3.5%) opted out of receiving intervention materials, 53 (0.2%) had undeliverable addresses, and 81 (0.3%) were deceased after randomization but before the intervention started. Thus, 23,653 (96%) intervention participants began receiving intervention materials.

Characteristics of randomized *WHISH* participants are shown in the table below.

Descriptive Statistics of *WHISH* Participants at Randomization – Overall and by Age Tertiles (072020)

	All Participants (n=49331)		<77 (n=16878)		77 - 82 (n=16220)		≥83 (n=16233)	
Category	n	%	n	%	n	%	n	%
Outcome Coverage								
WHI Adjudicated Only	10554	21.4	3849	22.8	3493	21.5	3212	19.8
CMS Only	30346	61.5	10094	59.8	9980	61.5	10272	63.3
WHI + CMS	8431	17.1	2935	17.4	2747	16.9	2749	16.9
Age, mean (SD)	79.7	(6.2)	73.1	(2.2)	79.4	(1.7)	87.0	(3.2)
Ethnicity								
Non-Hispanic White	41606	84.3	13544	80.2	13735	84.7	14327	88.3
Non-Hispanic Black / African American	4514	9.2	1946	11.5	1493	9.2	1075	6.6
Hispanic / Latina	1628	3.3	771	4.6	502	3.1	355	2.2
American Indian / Alaskan Native	151	0.3	73	0.4	45	0.3	33	0.2
Asian / Pacific Islander	923	1.9	375	2.2	277	1.7	271	1.7
Unknown	509	1.0	169	1.0	168	1.0	172	1.1
Current Smoker								
Yes	1216	2.5	603	3.6	385	2.4	228	1.4
No	48103	97.5	16268	96.4	15831	97.6	16004	98.6
Use of BP Meds								
Yes	30136	61.1	8770	52.0	9989	61.6	11377	70.1
No	19195	38.9	8108	48.0	6231	38.4	4856	29.9
Use of Lipids Meds								
Yes	21801	44.2	6841	40.5	7464	46.0	7496	46.2
No	27530	55.8	10037	59.5	8756	54.0	8737	53.8
BMI, mean (SD)	28.0	(5.8)	28.4	(6.3)	28.2	(5.8)	27.4	(5.2)
>30	14921	30.2	11305	67.0	11135	68.6	11943	73.6
≤30	34383	69.7	5561	32.9	5077	31.3	4283	26.4
Physical Functioning Score, mean (SD)	71.6	(25.2)	79.3	(21.8)	72.3	(24.1)	62.7	(26.6)
<65	15152	30.7	3241	19.2	4720	29.1	7191	44.3
65 – 75	8283	16.8	2382	14.1	2894	17.8	3007	18.5
76 – 89	8312	16.8	2921	17.3	2929	18.1	2462	15.2
≥90	17525	35.5	8328	49.3	5657	34.9	3540	21.8
Limited ability to go up one flight of stairs								
No, not limited	35051	71.1	13609	80.6	11762	72.5	9680	59.6
Yes, limited a little	10777	21.8	2650	15.7	3507	21.6	4620	28.5
Yes, limited a lot	3497	7.1	619	3.7	948	5.8	1930	11.9
Limited ability to walk one block								
No, not limited	38899	78.9	14854	88.0	12991	80.1	11054	68.1
Yes, limited a little	7294	14.8	1482	8.8	2330	14.4	3482	21.5
Yes, limited a lot	3130	6.3	542	3.2	897	5.5	1691	10.4
Physical Activity, MET-hr/wk, mean (SD)	11.7	(12.5)	13.5	(13.5)	11.8	(12.4)	9.6	(11.1)
Self-report Treated Diabetes Ever	8446	17.1	2922	17.3	2906	17.9	2618	16.1
WHI Outcomes								
CVD	5041	10.2	1005	6.0	1638	10.1	2398	14.8
MI	1799	3.6	341	2.0	578	3.6	880	5.4
CABG/PTCA	3030	6.1	620	3.7	1001	6.2	1409	8.7
Stroke	1632	3.3	301	1.8	518	3.2	813	5.0

WHI HT Trial Arm								
Active	5736	11.6	1841	10.9	1881	11.6	2014	12.4
Placebo	5645	11.4	1743	10.3	1863	11.5	2039	12.6
Not Randomized	37950	76.9	13294	78.8	12476	76.9	12180	75.0
WHI DM Trial Arm								
Intervention	6402	13.0	2221	13.2	2252	13.9	1929	11.9
Comparison	9932	20.1	3507	20.8	3508	21.6	2917	18.0
Not Randomized	32997	66.9	11150	66.1	10460	64.5	11387	70.1
HI CaD Trial Arm								
Active	7369	14.9	2572	15.2	2514	15.5	2283	14.1
Placebo	7079	14.4	2439	14.5	2436	15.0	2204	13.6
Not Randomized	34883	70.7	11867	70.3	11270	69.5	11746	72.4
WHI Study Component								
Clinical Trial	24186	49.0	8096	48.0	8297	51.2	7793	48.0
Observational Study	25145	51.0	8782	52.0	7923	48.8	8440	52.0

At Fred Hutch, Dr. Charles Kooperberg is the contact PI and will provide leadership guidance and statistical guidance. He will oversee presentation of materials for the DSMB and progress reports. As contact PI, Dr. Kooperberg will provide fiscal oversight, and be responsible for reporting to the NHLBI. He is the contact PI of the *WHISH* Data Coordinating Center (WHI-CCC) during this current phase of the *WHISH* trial; his activities for that project were identical to the current project. At Stanford University, Dr. Marcia Stefanick has been and will continue to be responsible for the development and delivery of the physical activity intervention, including all mailed materials, email and automated telephone systems that deliver monthly motivational messages to participants, the www.whish.org website and its physical activity tracking tool, and unscheduled communications between Intervention participants and staff. Dr. Stefanick is the contact PI of the *WHISH* Clinical Coordinating Center (referred as the *WHISH* Intervention site) during this current phase of the *WHISH* trial. She collaborates with Dr. Abby King to integrate behavioral principles and implement the PA intervention in an adaptive manner. At the University of California, San Diego, Dr. Andrea LaCroix will continue to provide leadership in all aspects of the *WHISH* trial including oversight of the accelerometry and physical activity monitoring activities at the UCSD Exercise and Physical Activity Resource Center (EPARC). At the University of Buffalo, Dr. Michael LaMonte will provide expertise on exercise physiology and accelerometer-measured physical activity and sedentary time, advice on intervention messages, and work with the multi-PI team on data analysis and interpretation, development of additional ancillary studies using these (and other) results as preliminary supporting evidence, and, help evaluate the proposed biomarkers in response to trial randomization. Except for Dr. Kooperberg, all other PIs will be blinded to the data and data analytic activities. Decisions regarding the project management to meet the scientific objectives will be made jointly by the PIs with the exception of Dr. Kooperberg, who will not be a part of making decisions regarding the intervention component of the study. Drs. Stefanick, LaCroix, and LaMonte will all take active roles in unblinded primary data analyses under the project specific aims during the entirety of the blinded intervention phase. All PIs will take an active role in ongoing evaluation of data quality and manuscript writing; and will provide support in all aspects of study administration and scientific dissemination.

3.1.1.b. Study Procedures, Materials, and Potential Risks

Institutional Review Board (IRB) Review. All Phase 2 procedures, data gathering tools, consent form (e.g., accelerometry) and other study materials will be provided to the Fred Hutch IRB for review and approval once developed and before implementation.

Study Procedures. Under the Zelen design, *WHISH* comparison participants participate solely through their usual WHI mailed annual medical history update. In the first year, the *WHISH* participants randomized to intervention received an invitational welcome packet describing the PA program and materials, and the opportunity to ask questions or request that they not receive materials by calling a toll-free telephone number. Only 3.5% “opted out” of receiving materials, though by the Zelen

experimental design, they continue to participate through their usual WHI mailed annual medical history update. They will be analyzed as Intervention participants in intention-to-treat analyses. The invitational packet included an initial *WHISH*ful Actions newsletter (designed by the Stanford Intervention team) which included key information regarding the Department of Health and Human Services (DHHS) PA guidelines for older adults and the Workout to Go pamphlet from the National Institute on Aging (NIA) *Go4Life*® program, featuring strength, balance, and flexibility exercises. Intervention participants who did not opt out received three additional mailings spaced a month apart that included the full *Go4Life*® *Exercise & Physical Activity: Your Everyday Guide*, a pedometer, a calendar for tracking their steps and activity, if they so desired, an introduction to and sample from the interactive voice response system (IVR) "health coach" phone call system, "Adriana", and tips for how to use the materials. After the three initial mailings, intervention participants have been receiving quarterly (seasonal) *WHISH*ful Actions newsletters which feature tips on physical activity, balance, flexibility, fall prevention, and muscle strengthening. Participants were provided with resistance bands, accompanied by examples of exercises, information on the www.whish.org website which features exercise videos, participant stories, advice from a geriatric physical therapist, and links to selected websites, such as *Go4Life*®, https://go4life.nia.nih.gov, and Health.gov 'Move Your Way' for adults https://health.gov/moveyourway/#adults. Comparison women have access to the key intervention messages, including the NIA *Go4Life*® materials and Health.gov sites (see details below, Materials).

To monitor changes in PA in the Intervention and comparison groups "objectively", a substudy in 2350 *WHISH* participants (1,182 comparison; 1,168 intervention) was conducted during phase 1 during which consenting participants wore a waist accelerometer for 7 days and nights at 6, 18 and 42 months after randomization and completed a sleep log for each of the 7 nights. During phase 2, an accelerometry substudy will again be conducted to monitor changes "objectively." Please see section 2.5. Recruitment and Retention for the recruitment plan.

The *WHISH* trial will leverage the planned Long Life Study 2 (LLS2) in the Women's Health Initiative Extension Study (2020-2027) by utilizing data and biospecimens from an LLS2. Specifically, through *WHISH* specific aim 2, we will determine the intervention effects on measures of healthy CV aging including physical function (PF) measured using the Short Physical Performance Battery (SPPB), sleep disturbances and insomnia symptoms, and depressive symptoms. Under the *WHISH* Exploratory Aim, we will create an experimental framework within *WHISH* and conduct pilot studies to determine whether the *WHISH* PA intervention influenced levels of novel biomarkers indicative of healthy CV aging and/or occult myocardial injury, such as N-terminal pro-brain natriuretic peptide (NT-proBNP) and high sensitivity Troponin T (hsTnT). A *WHISH* Biomarker Advisory Group involving US experts will vet the most promising biomarkers for pilot- testing. This aim will facilitate future *WHISH*/WHI Ancillary Studies. The specific aim 2 and exploratory aim are contingent on WHI Extension Study contract funding of the LLS2.

Materials. Women randomized to the PA intervention group were contacted by mail by the *WHISH* DCC (WHI- CCC) to obtain (passive) informed consent. This initial mailing included three items: (1) a cover letter from the WHI-CCC Principal Investigator with information about the *WHISH* trial and the 800-number to "opt out" of receiving materials for the *WHISH* PA Intervention; (2) *WHISHful Actions: National Recommendations for Physical Activity in Older Adults* (i.e., basic information on the national DHHS PA recommendations with health and safety tips)*;* (3) the *Go4Life® pamphlet "Workout to Go: A Sample Exercise Routine from the National Institute of Aging at NIH".* Women who did not "opt out" received an initial set of materials mailed from the Stanford University *WHISH* PA intervention site a month apart, including the *Go4Life*® book, *"Exercise & Physical Activity: Your Everyday Guide from the National Institute on Aging,"* a pedometer and calendar for tracking activity, and information on a website physical activity tracking tool. After this initial set of three mailings, mailed materials have included quarterly (seasonal) newsletters (*WHISH*ful Actions), a resistance band complemented by exercises, information about an automated interactive voice response system ('Adriana') which also provided a tracking tool, annual physical activity logging calendars, another pedometer, and a *WHISH* visor for sun protection. The

quarterly *WHISH*ful Actions newsletters included additional written information using American College of Sports Medicine "Exercise as Medicine" messages, the new 2018 DHHS Physical Activity recommendations for older adults, findings from the WHI OPACH study regarding benefits of light intensity physical activity and harms of sitting, and safety tips and advice from a geriatric physical therapist regarding gait and balance. Materials are developed to promote activity with friends, neighbors and family, (e.g., grandchildren), and facilitate social support. A survey was sent every year to obtain input from participants which is used to adapt materials to reflect their personal activity preferences and inserts are developed for three "target" groups based on self-reported physical function and physical activity levels from WHI forms. The website (with a secure login for participants who had been randomized to the intervention) was overhauled to be more user friendly for older women. Materials are described in more detail in the Research Plan and in the subsequent paragraphs until the potential risks section.

The *WHISH* pragmatically designed, PA intervention is an innovative "light touch", remotely delivered behavioral intervention, consistent with the multicomponent DHHS PA recommendations for older adults.[28, 29] *WHISH* applies state-of-the-science behavioral theory,[30-32] embedded in an iterative framework based on participant input from annual surveys and other sources, to motivate women to increase or maintain PA levels and reduce sedentary behavior. "Light-touch" refers to interventions delivered primarily by non-health professional staff and resources.[33]

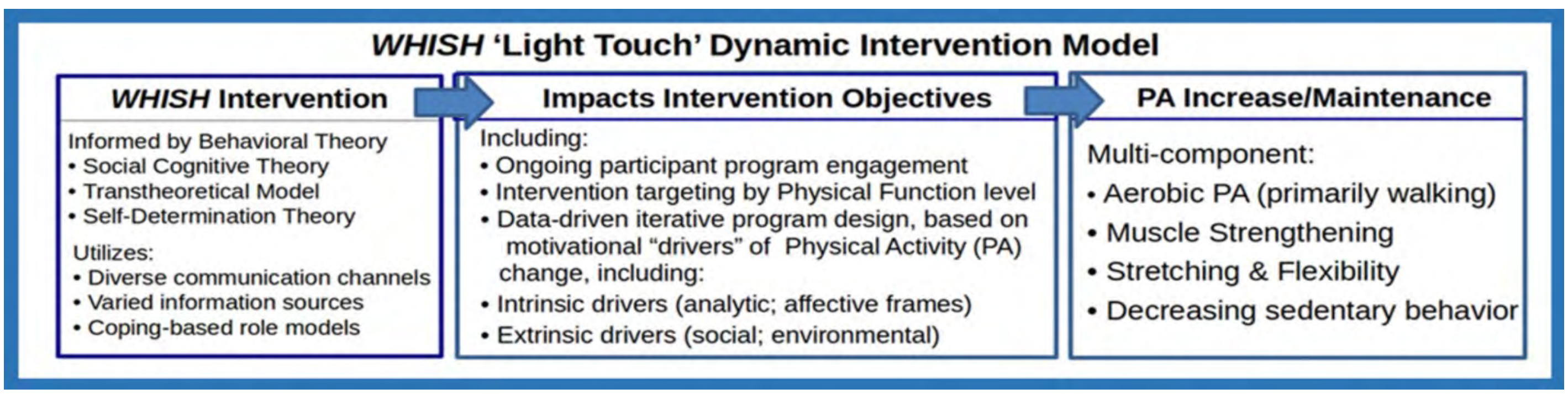

Initial mailings to Intervention participants included the NIA *Go4Life® Exercise & Physical Activity:* Your *Everyday Guide* book, easy-to-use PA tracking calendars, pedometers, resistance bands, and a toolkit of specific exercises. Seasonal (quarterly) "*WHISH*ful Actions" newsletters (e.g. Figure 4) emphasize walking and aerobic activities, muscle strengthening, balance and flexibility exercises, and reducing sedentary behavior, which women are encouraged to track. The multi-modal/multi-channel, remotely-delivered intervention also includes an algorithm-driven interactive voice response (IVR) system of tailored advice, support, and personal goal setting for the 98% of participants who are receiving the IVR outbound motivational messages, and email-based messages for the 33% of participants who provided email addresses, and a website (www.whish.org) with a self-monitoring (tracking) tool aimed at the 76% of *WHISH* participants who reported using the Internet and provides links to *Go4Life*®,[34] Health.gov, and other sites. Participants can also reach staff with questions and feedback through inbound email, phone, and mail channels throughout the intervention.

Intervention materials are continuously adapted and improved in response to participant engagement that occurs through mailed surveys, postcards, e-mail, and telephone contacts. Participants report their activity preferences, information on what motivates them to move more and be less sedentary, and their perceived barriers to PA. PA resources and intervention materials are adapted to reflect this input. An array of motivational drivers of PA change are addressed[35] including informational drivers, e.g., news and research stories featured in *WHISH* newsletters and on the *WHISH* website, affective drivers (thoughts and feelings), social drivers (friends and family, caregiver activities) and environmental cues and contexts (e.g., coping with weather, strategically posting PA reminders, etc.). Participants

themselves serve as "coping" role models through sharing their personal stories (and photos) about overcoming barriers to PA, e.g., recovery from injuries, illnesses, and personal losses, in regular "Life Happens" newsletter features, which are particularly well-liked by participants. The *WHISH* website offers even more participant stories, as well as videos of participants demonstrating exercises from the newsletters and links to a range of PA websites, such as Health.gov 'Move Your Way' for adults https://health.gov/moveyourway/#adults/), and materials reviewed and approved by a geriatric physical therapist who recommends specific exercises, advises on the instructions and provides safety tips.

Targeted Intervention Materials. To increase the salience and accessibility of messages to older women with diverse PF levels, targeted materials have been developed, based on participant feedback that materials were too challenging for some and not challenging enough for others. Starting with the Fall 2018 newsletter, which emphasized the health benefits laid out in the 2018 Physical Activity Guidelines (PAG) for Americans (Older Adults)[36], we created three sets of instructional materials to promote high intensity interval training activities (HITT) appropriate for women at different physical functioning levels, defined by a combination of physical function and PA scores based on each woman's most recent WHI- reported measures (PF=RAND-36[37] [higher score reflects greater PF]; PA=MET-hrs/week) (percentages in low, generic, and high function/PA categories= 36%, 32%, and 32%, respectively; see Table 5). These categories have been used to more specifically adapt and target *WHISH* materials to the needs and preferences of each group, starting with a "micro-experiment" (see below).

Table 5. Characteristics by Targeted Intervention Group.

Group	Low	Generic	High
N	7064	6122	6004
Age (ave (sd))	81.0 (6.0)	79.0 (5.8)	76.9 (5.2)
Current Age			
<77	24%	32%	44%
77-82	38%	32%	30%
>82	17%	31%	52%
PF (ave (sd))	40.7 (20.5)	70.4 (20.0)	91.7 (6.4)
Race/ethnicity			
White	37%	32%	31%
African-Amer	43%	30%	27%
Hispanics	31%	34%	35%
Asian/PI	29%	35%	36%

Launching of "micro-experiments" to optimize intervention adaptations for key subgroups. To inform more intensive targeting of the multi-component intervention to key subgroups based on PF and PA levels— the major factors predicting future PA—a micro-experiment was launched to assess the low, "generic", and high "targeted" inserts. Women in a lower function category were randomly assigned to receive either mailed instructional materials targeted to low function or more generic materials, i.e., a mix of different function and PA levels, while women in a higher function category were randomly assigned to receive either the generic materials or materials targeted to high function. Current age, body mass index, and walking aids differed between these two randomized groups (see Table 6), whereas both groups are composed of ~82% white, 10-11% black, 3- 4% Hispanic, 2-2.5% Asian/PI, 0.2-0.4% Native American or Alaskan women. Participant engagement (% of surveys and postcards returned) and responses to specific questions about the usefulness of intervention materials are currently being analyzed and will inform further targeting activities.

Table 6. Micro-Experiment: Targeted Intervention Groups.

	Random Low		Random Low Generic		Random High Generic		Random High	
N	2561		2518		2491		2522	
Current age	N	%	N	%	N	%	N	%
<77	582	22.7	637	25.3	948	38.1	999	39.6
77-82	938	36.6	850	33.8	937	37.6	926	36.7
>=82	1041	40.7	1031	40.9	606	24.3	597	23.7
BMI								
<25.0	708	27.7	687	27.3	946	38.0	955	37.9
25.0-29.9	878	34.3	808	32.1	928	27.3	913	36.2
>=30.0	973	38.0	1023	40.6	616	24.7	652	25.9
Aids								
No aid	1709	71.5	1676	71.2	2218	96.0	2247	95.1
Cane	438	18.3	449	19.1	64	2.8	91	3.8
Walker	238	10.0	224	9.5	26	1.1	25	1.1

Continuing Participant Engagement. The above suite of intervention and communication activities has resulted in a strong and sustained level of participant engagement *in this pragmatic trial, which women may not have sought out and were not actively recruited.* As of April 20, 2019, 86% of the initial 23,940 women (and >93% of racial/ethnic minority women) who were randomized to the Intervention and did not opt out and are not known to be deceased, *remain in the intervention.* Response rates to annual surveys have steadily increased each year; Survey #1, 49%; Survey #2, 53%; Survey #3, 63%; Survey #4, 72%.

Survey #4 response rates ranged from 63-65% in the oldest women and in women reporting low PF scores, to 77% in younger women and those with high PF, and >63% in every race/ethnic group, with

the greatest increase from early survey response rates demonstrated among African American *WHISH* participants. As only 45% of black women had returned Survey #2, we conducted a formative evaluation of barriers and enablers in a small set of African American “non-responders” to learn why these individuals hadn’t returned the surveys and to inquire about their physical activity. A couple of these personal stories were featured in the “Life Happens” section of the *WHISH* newsletter which may have resonated with other participants. Similar efforts are made to create materials, present stories, and include photos that reflect the heterogeneity of the *WHISH* study population and to develop materials that participants who respond to surveys report liking and wanting. Our mini-experiment of targeted materials will provide invaluable information on the success of this approach.

Planned Intervention Activities Moving Forward. Building on the successes from the initial intervention period, which include solid ongoing participant engagement and WHI data-derived changes observed in all PA behavior domains relative to the Comparison group our planned intervention aims are to:

- continue to focus on aerobic activities, particularly walking, occurring throughout the day, while emphasizing the importance of the continuum of PA intensities (light, moderate, vigorous) for older women, and promoting the concept of relative intensity, as experienced by the “Talk Test” (Light: can sing and talk while moving; Moderate: cannot sing, but can still talk; Vigorous: can only speak a couple of words at a time). In addition, a particular emphasis for the entire intervention will be on helping women optimize their ability and motivation to ***maintain their PA levels*** in the face of the typically observed ongoing decline associated with aging;
- increase the emphasis on the additional ***multi-component PA domains and a well-rounded routine*** that often receive less attention in PA interventions, but have been shown to be particularly important for older adults, i.e., muscle strengthening, stretching and flexibility, balance, and gait (presented as *“wiser walking*”);
- expand information related to reducing sedentary behaviors throughout the day, including breaking up inactive sitting or lying down, and replacing with light and moderate forms of PA;
- strive to increase survey response rates and participant engagement, including accessing the *WHISH* PA website which has been developed specifically for the *WHISH* study population of older American women.
- systematically explore the addition of “light-touch” mobile app interventions for the more than half of the *WHISH* Intervention women who reported having smartphones;
- conduct further micro-experiments to continue to advance intervention targeting and adaptations to specific subgroups who are particularly vulnerable to PA decline or diminished program engagement (e.g., low physical function participants).

Methods that we will employ to promote the intervention objectives include:

- continued targeted data collection to drive adaptive program design, including delivery of intervention components that particularly focus on four major domains of motivational drivers for PA, i.e., analytic (targeted and personalized facts and information concerning PA and its benefits), affective (thoughts and feelings), social (*WHISH* participant role models and success stories), and environmental drivers (increasing cues, prompts, and contexts that motivate PA);
- instructing participants in easy and safe field-based self-testing (e.g., portions of Rikli and Jone’s field-based physical function tests for older adults,[38] along with *Go4Life*®’s[34] self-assessment) that can provide individualized feedback and promote PA and physical function increases;
- increasing real-time data collection via the IVR-based outdialer system (which reaches 98% of participants), whereby participants can answer simple questions by phone, to continue to shape and enhance intervention relevance and potency;
- partnering with Stanford’s interdisciplinary bio-engineering Mobilize Center (http://mobilize.stanford.edu/) to test out the delivery of interactive smartphone apps aimed at increasing PA and decreasing sitting throughout the day.[39, 40]

Potential Risks. The *WHISH* PA Intervention is based on the DHHS Physical Activity Guidelines for older adults (originally 2008, now 2018) https://health.gov/paguidelines and has featured materials developed for the *Go4Life*® campaign of the National Institute on Aging (NIA) at the NIH (http://go4life.nia.nih.gov),

which was designed to help older adults fit exercise and PA into their daily lives. Materials include an easy-to-read guide *Exercise & Physical Activity: Your Everyday Guide from the National Institute on Aging*ˆ(available in Spanish) and the *Go4Life®* website which includes a **"Stay Safe"** section that states, "almost anyone, at any age, can safely do some kind of exercise" and "most people do not need to check with their health care provider first before doing physical activity". A geriatric physical therapist advises on and reviews print materials and videos posted on the www.whish.org website. Risks associated with the intervention are those typically associated with common physical activities like walking, including muscle soreness, fatigue, possible falls and other PA-related injuries. However, it is also true that sedentary behavior is associated with poor health outcomes including falls and fractures. No psychological, social, cultural, financial, and legal risks; risks to privacy and/or confidentiality; or other risks are anticipated.

Alternate treatments. All *WHISH* participants may be physically active on their own, including group and health clubs. The *Go4Life®* materials are available at no charge from the NIA *Go4Life®* website (go4life.nia.nih.gov) and all participants can access Health.gov 'Move Your Way' for adults https://health.gov/moveyourway/#adults/ and other public websites linked to the www.whish.org site.

3.1.2. Adequacy of Protection Against Risks

3.1.2.a. Informed Consent and Assent

Participants were randomized into intervention and comparison at equal rates (50-50) under the Zelen design. Under the Zelen design, a separately conducted consent process was not conducted. *WHISH* comparison participants participate solely through their usual WHI mailed annual medical history update. The *WHISH* participants randomized to intervention received an invitational welcome packet describing the physical activity (PA) Program, introductory PA materials, and the opportunity to ask questions or opt-out of receiving materials by calling a toll-free telephone number. The invitational mailing was approved by the Fred Hutch Institutional Review Board (IRB) to serve as a passive consent. *WHISH* intervention participants who did not opt-out began receiving additional intervention materials monthly for the first three months followed by quarterly (seasonal) mailings, monthly motivational messages delivered by telephone, and for women who provided email addresses, by email. Women may request no further mailings, calls, etc., which 12.8% of participants assigned to the Intervention have done to date. See 1.b. Study Procedures, Materials, and Potential Risks for details about the intervention materials. Participants who were selected for the Accelerometry Substudy received an invitational mailing that included a formal consent process by mail and phone. The invitational mailing included a cover letter from the *WHISH* DCC PIs, study description, toll-free number to call for questions about the study, and a copy of the consent form. Participants who returned a signed consent form were enrolled into the substudy.

3.1.2.b. Protection Against Risks

Women who receive the PA program intervention materials were encouraged to talk with their health care provider (HCP) before engaging in the PA intervention if they weren't already engaging in activity. At any time during the trial, these participants are encouraged to consult with their HCP about any new symptoms they are concerned about that they haven't previously discussed with their HCP. A licensed geriatric physical therapist reviews all materials and advises on exercises. The *WHISH*ful Actions newsletters provide tips regarding balance and fall prevention, including materials tailored for women who use walkers or canes, those who have trouble rising from sitting, and those who may not be able to stand (such as "sit and be fit" activities). These tips also are presented on the *WHISH* website (including video tutorials) and with monthly automated tips calls implemented through the outbound "Adriana" telephone system.

The privacy of participants and confidentiality of research data are protected through several mechanisms. The WHI Clinical Coordinating Center (*WHISH*-DCC) was required to submit to the NHLBI an extensive and detailed System Security Plan addressing how we ensure data integrity, security, and confidentiality. The WHI-CCC runs a standalone Windows-based network with its own Windows domain controllers, firewall, VPN, routers, and switches. All WHI database and analysis files are located on a file server located in the WHI-CCC Data Center, which sits behind an enterprise class Sonicwall firewall with Intrusion Protection enabled. The server runs Windows 2008 R2 Advance Server and Oracle, both configured with the latest security patches. Only WHI-CCC IT personnel required to maintain the server and data personnel are authorized to access the data. CCC employees who access confidential participant data have password-protected computers that are in locked offices. All WHI-CCC staff are required to sign a confidentiality agreement as well as attend HIPAA and IT security training. This helps ensure that staff are educated regarding their responsibility for the security and confidentiality of the WHI and CMS data.

3.1.2.c. Vulnerable Subjects

Not relevant to *WHISH* as no fetuses, neonates, pregnant women, children, prisoners, institutionalized individuals or other vulnerable populations are included.

3.1.3. Potential Benefits of the Proposed Research to Research Participants and Others

The direct benefits of participating in a regular exercise program may be increased aerobic fitness, muscle strength, flexibility, and balance, possible reduction in falls and/or fractures, in addition to a possible reduction in the risk of developing cardiovascular disease. Evidence suggests that women who increase their PA levels develop fewer disabilities and are more likely to remain mobile and have better physical and cognitive function. Women will also learn more about monitoring and improving their fitness level in general. Although these potential benefits are stated in the DHHS Physical Activity Guidelines for older adults, many have never been proven in large, randomized clinical trials, which is the main purpose of the *WHISH* trial.

For the Accelerometry Substudy, wearing an accelerometer for 7 days entails only minor risks, such as irritation caused by the elastic belt around the waist, or inadvertent pinching by the belt clasp. In addition, wearing the belt and device during the day and night may cause minor discomfort or annoyance. If women experience discomfort or difficulty wearing the accelerometer, they are advised to call study staff and report these problems. A better fitting belt may be supplied, or the woman may be advised to discontinue the monitoring.

3.1.4. Importance of the Knowledge to be Gained

The information obtained in this study could influence national guidelines on physical activity for older women, and influence how health care providers advise older adults about PA. The study findings could contribute to efforts to promote PA in older adults, leading to improvements in health status of older adults. The study is especially well positioned to determine how much and what kind of physical activity is needed to reduce the risk of cardiovascular outcomes in older women, including how much moderate/vigorous activity is needed, if any, as well as potential benefits of light intensity activity and reduced sedentary behavior to cardiovascular outcomes. Information, additionally and importantly, will be obtained about the safety of PA in this population and about possible improvements, or at least lesser age-related decline, of physical function and mobility.

Overall, we consider the risks associated with slowly increasing PA levels to achieve national recommendations regarding PA for older adults to be reasonably low for the PA intervention participants, as is the burden associated with providing information on changes in behavior. The potential benefits to

participants are increased fitness and the positive consequences therein. The findings may influence national guidelines on physical activity for older adults, which is of tremendous value. Thus, on balance, the overall risks are minimal relative to the expected benefits.

3.2. Is this a multi-site study that will use the same protocol to conduct exempt human subjects research at more than one domestic site? No

3.3. Data and Safety Monitoring Plan

The *WHISH* trial has a Data Safety Monitoring Board (DSMB), appointed by the National Heart, Lung, and Blood Institute (Eugene R. Passamani, MD, chair) that is meeting annually. The DSMB approved a monitoring plan. This plan was adapted to the original proposal and assumed a trial end in 2020.

The monitoring plan below has two changes compared to the approved monitoring plan. (i) There is a change in meeting times and numbers, as they would be adjusted to accommodate an 8-year follow-up. (ii) There will be an interim monitoring taking place in the spring of 2022. These changes naturally would have to be approved by the DSMB. The additional outcomes that we are proposing in this extension (SPPB, Sleep Disturbance, and Depressive Symptoms) are not used for monitoring under the currently approved plan. The DSMB would see data on these outcomes, but they are not discussed in this monitoring plan.

3.3.1. Protocol Defined Outcomes

The primary outcome of the *WHISH* trial is

- CVD: defined as (first event since enrollment in *WHISH* of) MI, stroke, cardiovascular (CV) death

Safety outcomes are:

- Non-CV death
- Hip fracture
- Revascularization
- Falls
- Clinical (non-hip) fracture

Secondary outcomes are:

- Venous thrombosis (VTE)
- Peripheral artery disease (PAD)
- Physical function, as determined by the Rand-36 score

Phase 2 adds to the above:

- Short Physical Performance Battery (SPPB) in a subset
- Sleep disturbance
- Depression

3.3.2. Monitoring Guidelines

Trial monitoring for WHISH has two main purposes, assuring the safety of participants and that this effort will result in an adequate test of the hypotheses.

During the first phase of WHISH, the WHISH trial had no formal monitoring boundaries for stopping early for CVD efficacy: the intervention is already publicly available, thus the benefit of getting a definitive answer was deemed to outweigh the benefit of making a less clear result public. This position was first

offered by the DSMB and agreed upon by the investigators in the pre-trial discussion and justified by the following: 1) the need for a broader assessment of the benefits and risks of increasing physical activity in the elderly, 2) the benefits for CVD being strongly anticipated from prior studies mostly in younger populations, and 3) the availability of the intervention materials means that the study is not denying anyone access. Similarly, the trial was not be monitored for futility with respect to the cardiovascular outcomes, recognizing the importance of establishing changes in physical activity by itself.

In discussions with the NHLBI it was agreed upon that during the second phase of WHISH there will be one formal monitoring (interim analysis) of the primary CVD/stroke/Cardiovascular Death endpoint, both for benefit and futility. This interim analysis will take place in the spring of 2022. Details are provided in the next section, 3.3.3. Interim Analysis for the WHISH Trial to be taking place in 2022.

Safety is our highest concern. This physical activity intervention, which is built upon materials provided by the National Institute on Aging, is thought to be safe for older Americans. It has not been tested, however, in a population of this size and so there is real value in documenting the impact of this intervention on multiple health conditions.

Among the safety outcomes, non-CV death and hip fracture are much more severe than the others (revascularization falls and clinical (non-hip) fracture) so safety monitoring guidelines will be defined only for the more severe outcomes, although data on all outcomes will be provided regularly. The primary outcome (CVD) should also be monitored as a (severe) safety outcome. CVD, non-CV death, and hip fracture will be monitored for safety at the (annual) DSMB meetings after Medicare data is available. The DSMB decided for the first phase of WHISH that each of the three outcomes would be monitored for safety at each of four monitoring meetings using a Z-value of 2.51. Based on simulations, the cumulative probability that the Z-statistic for one of these outcomes will exceed 2.51 at the first four meetings, if in truth there is no effect, is approximately 5%, assuming that the three monitored safety outcomes are independent. After WHISH is extended there will be three additional monitoring meetings, bringing the total to seven. Based on simulations, the cumulative probability that the Z-statistic for one of these outcomes will exceed 2.51 at the any of the eight meetings, if in truth there is no effect, is approximately 6.3%, assuming that the three monitored safety outcomes are independent.

Increased levels of activity in the intervention group may uncover symptoms of angina or claudication, and lead to revascularization procedures (coronary and peripheral artery, respectively). These procedures could actually reduce the rate of subsequent, more serious complications, such as myocardial infarction, and hence would not carry the same adverse safety signal as the other events to be monitored in WHISH. There are other plausible explanations for possible differences in revascularization rate. Similarly, there are multiple explanations of why rates of falls may differ between both arms of the trial. Therefore, while CVD and hip fracture are monitored outcomes, rates of revascularization and falls are reported but not formally monitored since the clinical implications for differences in these rates may or may not be apparent at the time they are observed.

Medicare data obtained from CMS is available in annual “batches”. Formal monitoring of the WHISH trial will take place in the late spring or early fall, at the time of the first DSMB meetings after receipt of Medicare/CMS data. These analyses would include adjudicated data for women in the WHI Medical Records Cohort (MRC) through the approximate date at which the Medicare data becomes available (see Section 3.3.8). The amount of follow-up available to women that are part of the MRC will be approximately the same as that for those women whose data is available through Medicare/CMS.

A final analysis including all available Medicare/CMS data, as well as adjudication of all self-reports not covered by Medicare/CMS through the end of the study will be presented within six months after that.

At DSMB meetings where there is no formal monitoring, updated outcomes data will be presented for informational purposes.

Additional analyses of safety outcomes will be provided to the DSMB at each meeting, as described in Section 3.3.3.

Interim analyses will employ use of a constant boundary for the three safety endpoints, as described above.

3.3.3. Interim Analysis for the WHISH Trial to be taking place in 2022

Background: An interim analysis for the WHISH trial will be carried out in the spring of 2022 (before July 1, 2022). The analysis will focus on the primary WHISH outcome of CVD/stroke/cardiovascular death. The analysis will allow for possible early stoppage for either benefit or futility. We propose conservative bounds/approaches, as we believe that there is much to be learned from the continuation of the WHISH trial for outcomes other than the primary outcome (e.g. for the secondary outcome of physical function) and subgroups of particular interest.

It is expected that at the spring of 2022 approximately 65% of the primary outcomes for the WHISH trial will have been observed.

Stopping for benefit: the null hypothesis for the WHISH trial is that there is no cardiovascular benefit, with an alternative hypothesis of cardiovascular benefit. This hypothesis is to be tested at a two-sided alpha level of 0.05; thus the test for benefit is one-sided at 0.025. The test-statistic for the primary outcome is already computed for each DSMB meeting using a stratified Cox proportional hazard model. We propose to apply an O'Brien-Fleming (OBF) boundary to this test-statistic. With one interim analysis at information of about 0.65 of the projected total, the OBF boundary is $Z=2.547$; the critical value at the end of the trial would correspondingly be adjusted from $Z=1.965$ to $Z=1.990$, to correct for the earlier look. The interim analysis uses alpha=0.00543 of the total alpha of 0.025 assigned to benefit.

Stopping for futility: we propose to calculate conditional power for the primary WHISH outcome using the same procedure as we used in the calculations provided to NHLBI leadership in January of 2020. These calculations, in turn, were modifications of the unconditional power calculations that were presented in our application for R61-HL151855 (the application for the continuation of WHISH). Details are given below. We propose that consideration be given for stopping for futility at that point if the conditional power for the primary outcome is below 20%. However, we note that, depending on the results for the secondary outcome, there may be reason to continue the trial even if the conditional power of the primary outcome is below this value.

These proposed statistical boundaries represent a change from the current plan adopted by the WHISH DSMB and thus will require their ratification. Further, these statistical boundaries do not address the complexity associated with these decisions when multiple clinical outcomes may be affected on different timescales. Thus, the DSMB will need to weigh the totality of the available evidence as well as what could yet be gained relative to any ethical demands when considering early termination.

Details of the proposed procedure to compute the conditional power

1. We estimate the currently observed event rates for the primary outcome of CVD and the censoring outcome (non-CVD death) pooled over both arms, smoothed over 5-year windows (e.g. the rate at age 86 is the average of the rates between ages 84 and 88). The rates (which will be updated in 2022) that were used in January 2020 are shown in Figure 1 of the appendix to the Proposed Interim Analysis Plan for the WHISH trial document, reproduced here. We note that the rates are almost identical to those shown in the proposal, as computed in 2019.

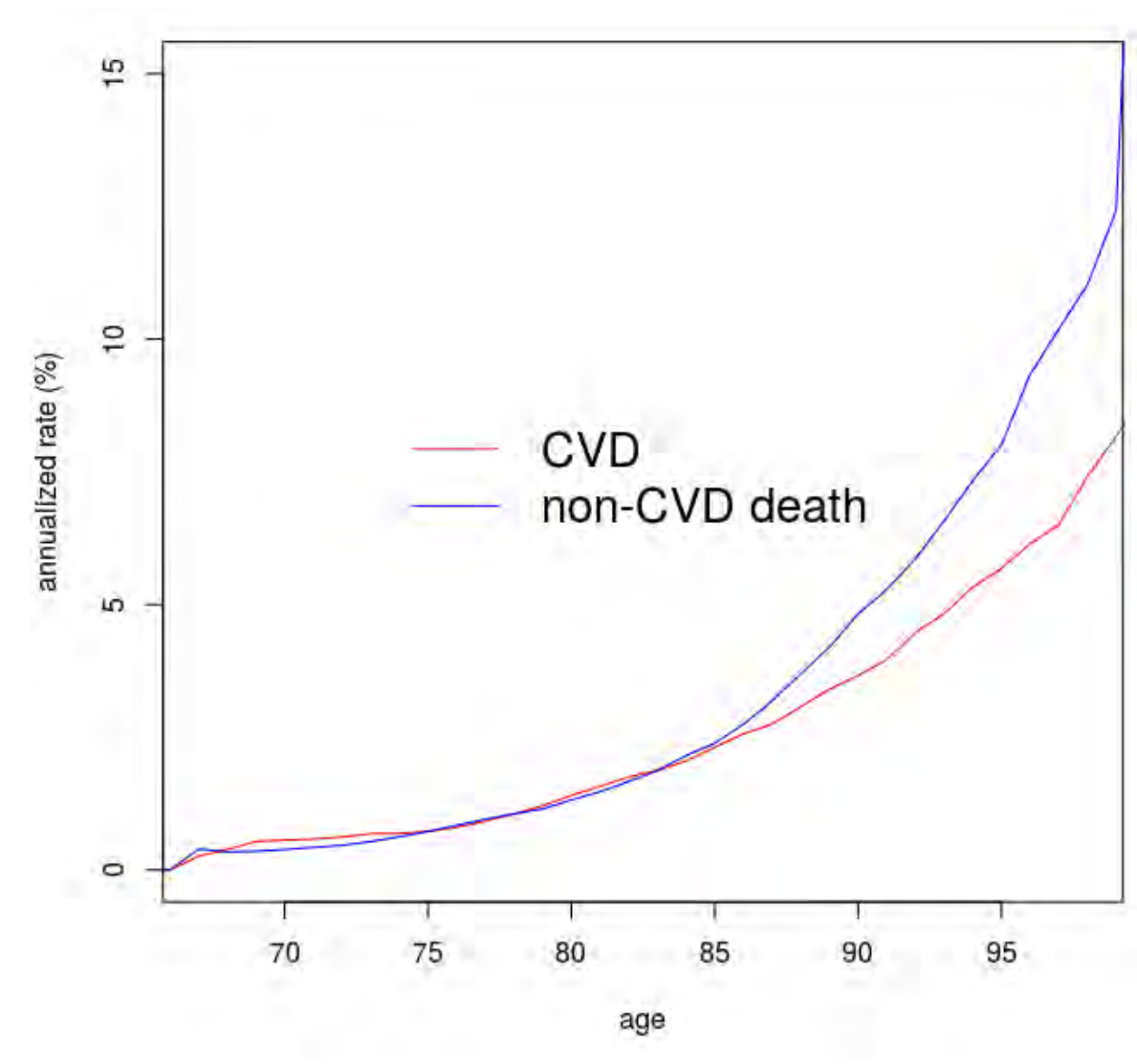

Figure 1. Observed incidence rates for CVD and non-CVD death in WHISH.

2. For participants who have not yet observed the primary WHISH event, and who have not been censored, we assume an end of follow-up of December 31, 2024.
3. For the assumed intervention effect, we will assume an effect of 8.5%, which is what we assumed in our proposal.
4. The power is then computed by simulation.
   a. If a participant had a CVD event (*T*) or a censoring event (*C*, non-CVD death) occur by the time of the database closure of the interim analysis, these are the event (or censoring) time for that participant.
   b. For participants alive and without events at the time of the database closure of the interim analysis, we generate possible event times *T* and censoring times *C* according to the rates computed at (1) starting at their last follow-up time. We create a difference in the event time rates assuming a difference of 8.5% in event rates of CVD for the remaining study period. If the participant had no event and was not censored before December 31, 2024, the participant is censored on December 31, 2014.
   c. We set, as in a standard survival analysis, $X=\min(T,C)$ and $\delta=(\text{Ind}(T<C))$. We fit a proportional hazard model, correcting for age stratum (as is done in the primary analysis), and compute the P value for intervention effect.
   d. For each of the 100,000 simulations we count how often P<0.025, corresponding to the alpha=0.025 assigned to benefit.

3.3.4 Reporting of Primary, Safety and Secondary Outcomes

At each interim analysis we will show for each of the primary, safety, and secondary outcomes intervention effect sizes (unadjusted 90%, unadjusted for multiple comparisons), confidence intervals, and Z-statistics. For all of these outcomes, except for falls and physical function, these estimates will be based on Cox-proportional hazards models. The rate of falls will be compared using Poisson regression. For physical function the mean level of physical function between the groups will be compared using linear regression with a possible log transformation to improve normality.

In addition to the individual outcomes, we will also provide analyses for the first event of any of CVD, hip fracture, or non-CV death ("global index"), noting that this global index is not a formal part of the monitoring plan, but can be used by the DSMB to balance effects if different outcomes have effects in different directions. The randomization of the *WHISH* trial is stratified by outcomes data source (MRC or

Medicare), age on April 1, 2015 (in tertiles), race/ethnicity, and region (West, Midwest, Northeast, and Southeast). The analyses will also all be stratified by the same categories.

The formal analyses will be based on confirmed outcomes (adjudicated or using Medicare/CMS data); however, we will also provide analyses where adjudicated outcomes are complemented with self-reported, but not-yet-confirmed, outcomes. We note here that in WHI, MI and stroke events are confirmed on review for about 60% of self-reports. For hip fracture about 80% of self-reports are confirmed.

All analyses will be carried out using an intent-to-treat analysis, including all women randomized: participants who “opt out” will be included in all analyses in their assigned arm for monitoring and primary reporting purposes. In particular, deaths that occurred between randomization and the start of intervention are included in the analyses. Similarly, participants who stop the intervention, or participants who become ineligible will remain in these analyses. For safety considerations, intent-to-treat analyses may be supplemented with analyses restricted to the women in the intervention who consented to receive the intervention, or to women who made noteworthy changes in exercise levels.

Additional analyses will be provided for these pre-specified subgroups:

- Participants with adjudicated outcomes versus those with only Medicare data.
- Stratification by age in tertiles.
- Analyses excluding participants who “opt out”.

For approximately 10,000 participants we will obtain both Medicare and adjudicated outcomes. For these participants the adjudicated outcomes will be used for monitoring. Analysis comparing the Medicare and adjudicated outcomes for these participants will be presented to provide insight in how comparable both sources of data are.

3.3.5. Reporting of Physical Activity Levels

Self-reported physical activity levels are ascertained on an annual basis from all participants in both arms of the trial through Form 521. Reports will include (self-reported) time spent walking, METS spent while walking, frequency of activities and total time spent on activities as assessed by the Community Healthy Activities Model Program for Seniors (CHAMPS) instrument (1).

An Accelerometry Substudy is carried out in 2000 participants in both arms of the study (1,000 in each arm) at approximately 6, 18, and 36 months after randomization, an additional round of accelerometry will be carried out in 2021 or 2022. This study will provide a more unbiased estimate of the activity levels than the self-reported activities. We will provide results from this study to the DSMB, including computed average activity level, and time spent sedentary, and in light and moderate/vigorous activity.

3.3.6. Reporting of Intervention Activities

We will report on the following intervention activities:

- Percentage of women in the intervention arm opting out.
- Percentage of women completing the Accelerometry Substudy, by arm.
- Activity levels of the intervention women.
- Engagement with the intervention: e.g., percentage of women returning surveys.

3.3.7. Reporting of Additional Outcomes

We will report on the following outcomes in manners similar to the primary, safety, and secondary outcomes:

- Cancer (including specific sites)
- Context of fall (inside/outside, during exercise, etc.).

3.3.8. Timeline for Outcomes Data Collection and Availability

For logistical reasons, *WHISH* participants were initially randomized in batches: the first 9,865 subjects (half intervention, half comparison) were randomized in April 2015, the next 30,801 were randomized in May 2015, and the remaining subjects were randomized in July 2015. These numbers included a small number of WHI participants who were deceased at the time of randomization, but for whom that was not known. The Consort Diagram (in Section 2.5) presents that information. The enrollment time for any *WHISH* participant was the first day invitation letters are mailed for subjects in the batch the subject was in, irrespective of whether the subject was randomized to intervention (and received such a letter) or was a comparison.

The average amount of time participants were enrolled in *WHISH* was about 7 months by December 31, 2015.

Self-reports are collected annually on WHI Form 33. Because of the annual cycle, there is an average 6-month delay in the outcomes collection of self-reports, plus an additional several weeks because of delays of returning the forms. Thus, self-reported outcomes will have an average 7-month delay.

The outcomes documentation and adjudication process takes on the average 2-3 months. Thus, outcomes for participants with full adjudication will have an average 9-10 month delay.

Medicare data is obtained from CMS on an annual basis. We expect to receive partial CMS data for the previous year in February of the next year, and (virtually) complete data by the following July. It will take at least two months to incorporate CMS data in the reports for the DSMB. Therefore, if the DSMB meetings are in March of any given year, the data for the subjects for whom we have CMS data will be approximately 15 months out of date. At the end of the study we will adjudicate all self-reports that were not yet covered by CMS data, thus reducing the delay for these participants to the same 9 months as for the participants with adjudicated outcomes.

Death reports are obtained from proxies as they occur, or when Form 33 data gets collected. It is important to note that reports of death may be obtained differentially in the two arms of the trial because of more frequent contact with intervention participants. To reduce the bias that this may introduce, proxy reports of death will only be included in the analysis at the time a participant was due for her Form 33. Periodic NDI searches occur in late spring – resulting in additional death reports typically after the proxy reports, but well ahead of the Medicare data. Rules regarding the classification of death, in particular when deaths are not yet adjudicated, are discussed in Section 3.3.8.

The proposed extension of *WHISH* will be funded as a 4-year project through 2024. It is envisioned that the trial will have a no-cost extension, allowing for a final analysis based on the database on January 1, 2025.

3.3.9. Outcome Definitions

Self-reports

- All clinical outcomes and falls are self-reported annually on WHI Form 33.
- Deaths are reported by proxies. Proxy reported deaths are only “counted” after the due-date of Form 33, to prevent differential ascertainment between both trial arms.

Validated outcomes for participants in the Medical Records Cohort (roughly 40% of the WHISH cohort, defined as former WHI Hormone Trial participants plus all African American and Hispanic participants):

- All adjudicators involved in WHI are blinded to *WHISH* treatment assignment.
- Stroke is adjudicated by neurologists using standard WHI procedures, including the Toast classification.
- Cancer is currently adjudicated by SEER coders using standard WHI procedures. Cancer is also adjudicated for participants not in the Medical Records Cohort. At the time of writing of this monitoring plan, it has not been determined whether cancer outcomes will continue to be adjudicated for WHI. If cancer will not be adjudicated, we will develop CMS algorithms, and use self-reports, if needed.
- All other clinical outcomes, except for clinical (non-hip) fractures, are adjudicated by physician adjudicators using standard WHI procedures.
- Clinical (non-hip) fractures are not adjudicated. In WHI it was found that fact of fracture (though not the site of the fracture) was self-reported at a high accuracy of about 80-85%. Finger and toe fractures are not counted for *WHISH*.
- Cause of death is adjudicated by physician adjudicators using standard WHI procedures. Not-yet- adjudicated death is classified with non-CV death until adjudication is complete. (The coding of death provided by NDI has been mapped to the classification used for WHI by physician adjudicators.)

Medicare/CMS outcomes:

We use the following algorithms to identify clinical outcomes using Medicare/CMS data:

- MI: Diagnosis codes of 410.x0 or 410.x1 in any position. (2)
- Stroke: Diagnosis codes 430, 431, 433.x1, 434.x1, and 436 in any position. Codes 430 and 431 correspond to hemorrhagic stroke, codes 433.x1, 434.x1, and 436 correspond to ischemic stroke. (3)
- Hip fracture: Diagnosis codes 820.00, 820.01, 820.02, 820.03, 820.09, 820.10, 820.11, 820.12, 820.13,

  820.19, 820.20, 820.21, 820.30, 820.31, 820.8, 820.9 in any position.
- Coronary artery bypass graft surgery: procedure codes 36.1x, 36.2.
- Percutaneous coronary intervention: procedure codes 00.66, 36.0, 36.00, 36.01, 36.02, 36.05, 36.06, and 36.07.
- Venous thrombosis (VTE): Pulmonary embolism (PE): diagnosis codes 415.11, 415.19 in any position, and Deep venous thrombosis (DVT): diagnosis codes 453.40, 453.41, 453.42, 453.8 in any position.
- Peripheral artery disease (PAD): Diagnosis codes (440.20 – 440.24, 440.9, 443.9, 444.22, 444.81, 447.1, 443.81, or 250.70 any position) AND either (1) a procedure code for surgery (39.50, 39.90, 00.55, 17.56, 99.10, 38.08, 38.14, 38.16, 38.18, 39.25, 39.29, 84.11, 84.12, 84.15, or 84.17 any position) or (2) a procedure code for imaging (88.01, 88.42, 88.47, 88.48, 88.76, 88.77 any position) without a procedure code for surgery. The diagnosis and procedure codes must occur during the same hospitalization.

Death:

- Fact of death can be proxy-reported, determined from the CMS/Medicare data, or identified from the NDI data.
- WHI Form 124. This form codes the underlying cause of deaths. Codes 11-19 are considered CVD death; all other codes are coded as non-CVD death.
- Cause of death for participants in the Self-Report Cohort (SRC) (as well as those in the MRC for whom insufficient documents can be collected) are determined using NDI data according to rules determined by the WHI Outcomes Adjudication Committee.
- For deaths not yet adjudicated, the fact of death and the date on the proxy report, are used in the monitoring tables. This differs from clinical outcomes, which are only used after the adjudication is complete, as death adjudication often takes substantially longer than adjudication for clinical outcomes, and NDI data will typically be available later than CMS/Medicare data. Date of death on proxy-reports is, however, not always accurate.
- The underlying cause of death for not-yet-adjudicated deaths are taken to be unknown, and are combined with the non-CVD death. The one exception is that not-yet-adjudicated death for participants who have an MI or stroke within the last six months before their death that is confirmed either by an adjudicator or by Medicare/CMS data are taken to be CVD death. When adjudication is complete, these deaths are reclassified, if needed.

For additional information see:

Stewart AL, Mills KM, King AC, Haskell WL, Gillis D, Ritter PL. CHAMPS physical activity questionnaire for older adults: outcomes for intervention. Med Sci Sports Exerc 2001;33(7):1126-41.

Hlatky M, Ray RM, Burwen DR, Margolis K, Johnson KC, Kucharska-Newton A, Manson JE, Robinson JG, Safford MM, Allison M, Assimes T, Bavry A, Berger J, Cooper-DeHoff RM, Heckbert S, Li W, Liu S, Martin L, Perez M, Tindle H, Winkelmayer WC, Stefanick M. Use of Medicare data to Identify coronary heart disease outcomes in the Women's Health Initiative. Circ Cardiovasc Qual Outcomes 2014;7(1):157-62.

Lakshminarayan K, Larson JC, Virnig B, Fuller C, Allen NB, Limacher M, Winkelmayer WC, Safford MM, Burwen DR. Comparison of Medicare claims versus physician adjudication for identifying stroke outcomes in the Women's Health Initiative. Stroke 2014;45(3);815-21.

3.4. Data and Safety Monitoring Board

WHISH is monitored under the Women's Health Initiative Data and Safety Monitoring Board

3.5. Overall Structure of the Study Team

The Multiple Principal Investigators (Drs. Kooperberg, Stefanick, and LaCroix) comprise the Executive Committee which oversees the activities at Fred Hutchinson Cancer Research Center (including study monitoring and data management) and all participating subcontract sites: Stanford University (the *WHISH* physical activity intervention site), the University of California, San Diego (the Accelerometry Substudy), and the University at Buffalo (Aim 3 activities). The Executive Committee will meet bi-monthly via Zoom videoconference and in-person 3 times annually (in May at the WHI Investigator meeting; in July at Stanford; and each fall for the DSMB meeting) to review all aspects of the Research Plan. This includes the following activities:

1. Review of scientific progress including timeliness of outcomes ascertainment, completenessof data collection, and monitoring of data quality;
2. Evaluation intervention materials and intervention effects on self-reported and accelerometer measures of behavior change;

3. Preparation of annual DSMB reports, and presentations for the annual meetings;
4. Reporting to the WHI Executive Committee, Ancillary Study Committee, and at the WHI Annual Investigator meetings on study progress;
5. Implementing the data sharing plan for *WHISH* intervention and data elements at the end of the trial;
6. Preparing manuscripts for publication, and abstracts and symposia for scientific meetings.

The organizational structure of the study team is depicted in the figure below.

Study Team

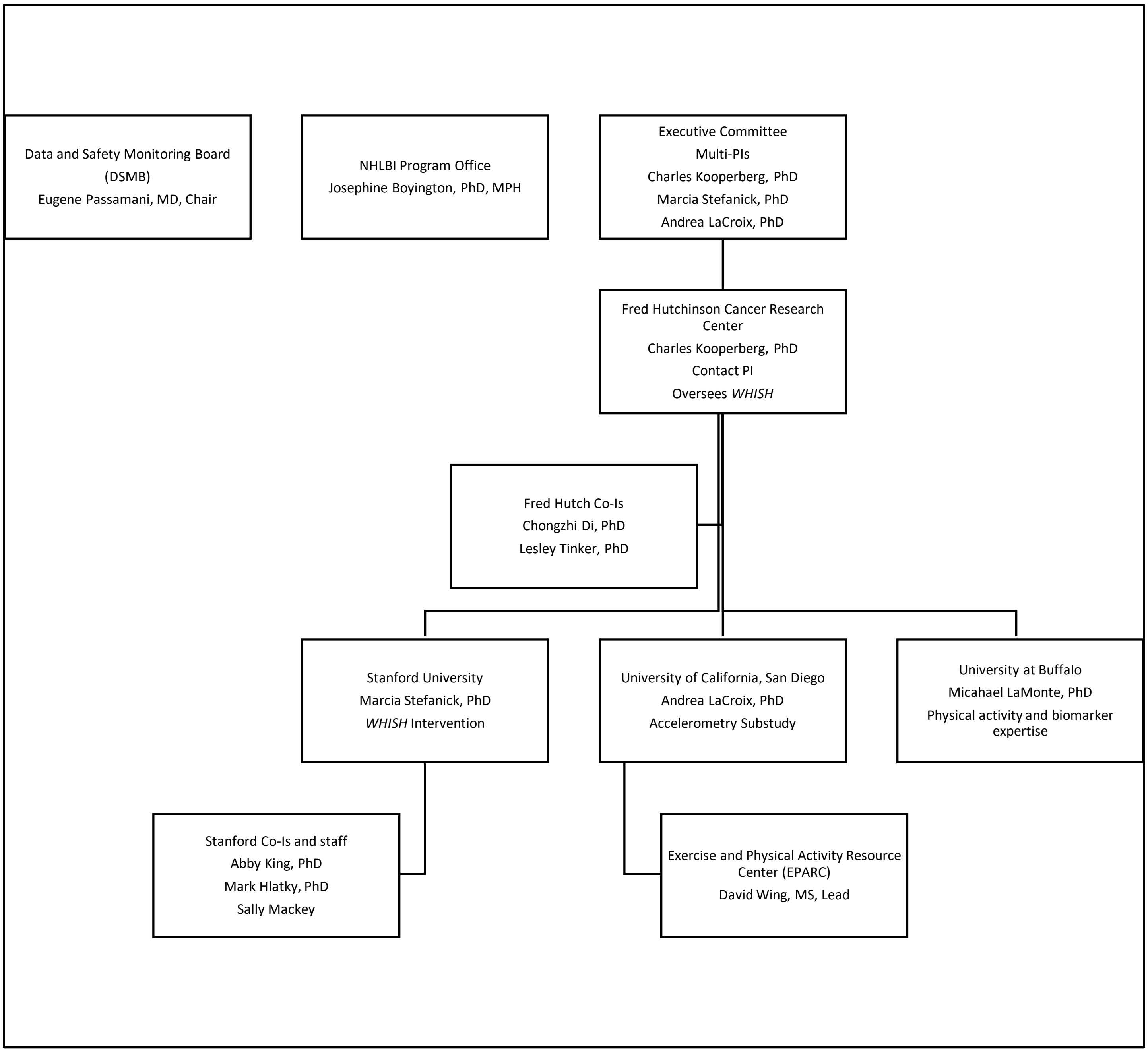

SECTION 4 – PROTOCOL SYNOPSIS

4.1. Study Design

4.1.a. Detailed Description: See Protocol Part 1, Section 4.

4.1.b. Primary Purpose: Prevention

4.1.c. Interventions (from ClinicalTrials.gov as of June 2019):

Type	Name	Description
Behavioral (e.g., Psychotherapy, Lifestyle Counseling)	Physical Activity	The PA intervention will consist of a multimodal activity program of aerobics, balance, strength, flexibility. The intervention will involve encouraging participants to increase all forms of PA throughout the day and to decrease sedentary time, such as sitting. This may include activities such as leisure sports, gardening, use of stairs instead of escalators, leisurely walks with friends, and less use of remote control devices. The intervention is conducted primarily by mail with website support and resources available.

4.1.d. Study Phase:

Is this an NIH-defined Phase III Clinical Trial? Yes

4.1.e. Intervention Model: Parallel

4.1.f. Masking: Yes (Outcomes Assessor)

4.1.g. Allocation: Randomized

4.2. Outcome Measures: See Sections 3.3.1. and 4.3.

4.3. Statistical Design and Power

As agreed upon with the *WHISH* DSMB, the outcomes in *WHISH* are:

1. CVD (composite of myocardial infarction, stroke, cardiovascular death)
2. Non-cardiovascular death
3. Hip fracture
4. Clinical (non-hip) fractures
5. Falls
6. Venous thromboembolic event
7. Peripheral artery disease
8. Physical function (Rand-36)
9. Coronary revascularization

Phase 2 adds to the above:

10. Short Physical Performance Battery (SPPB) (in a subset)
11. Sleep disturbance - WHI Insomnia Rating Scale
12. Depressive systems scale (CES-D)

As described elsewhere, 49,331 WHI participants have been randomized to either intervention or comparison in *WHISH.* All outcomes are ascertained on all (surviving) participants, except for the SPPB (outcome #10), which will only be ascertained on participants that are part of the WHI Long Life Study 2. Clinical outcomes are based on self-report and adjudication and on reports by the Center for Medicare

and Medicaid Services (CMS) of Medicare outcomes and information from the National Death Index (NDI), as described in Section D of the Research Plan. The other outcomes are based on self-report, or measured at an in-home visit (SPPB, #10).

For the time-to-event outcomes (1-4, 6, 7, and 9 above), effect estimates will be based on Cox-proportional hazards models. The rate of falls (5) will be compared using Poisson regression. For the remaining outcomes (8 and 10-12) the mean level of physical function between the groups will be compared using linear regression with a possible log transformation to improve normality.

The analyses will be stratified by outcomes data source (adjudicated or Medicare (CMS) data), age on April 1, 2015 (in tertiles), region of residence (West, Midwest, Northeast, and Southeast), and race/ethnicity. Using standard risk-set in survival analysis, participants who change from fee-for-service to managed care will have their self-reports adjudicated after their switch and will "move strata" at the time of the switch. Similarly, participants who have two types of data, but do not return their self-report Form 33, will have their outcomes after their last returned Form 33 assessed using CMS data.

All analyses will be carried out using an intent-to-treat analysis, including all women randomized: participants who "opt out" will be included in all analyses in their assigned arm for monitoring and primary reporting purposes. For example, the few deaths that occurred between randomization and the start of intervention are included in the analyses. Similarly, participants who stop the intervention, or participants who become ineligible will remain in these analyses. For safety considerations, intent-to-treat analyses may be supplemented with analyses restricted to the women in the intervention who consented to receive the intervention, or to women who made noteworthy changes in exercise levels.

Additional analyses will be provided for these pre-specified subgroups:

- Participants with adjudicated outcomes versus those with only Medicare data.
- Stratification by age in tertiles.

For several thousand participants we obtain both Medicare and adjudicated outcomes. For these participants the adjudicated outcomes are used for monitoring. Analysis comparing the Medicare and adjudicated outcomes for these participants are presented to provide insight in how comparable both sources of data are.

We note that not all outcomes are used for interim monitoring (see the DSMB plan, Section 3.3, for details); however, all outcomes are provided to the DSMB at each monitoring meeting.

Power

This section provides more details on how we obtained the power estimates described in the Research Plan.

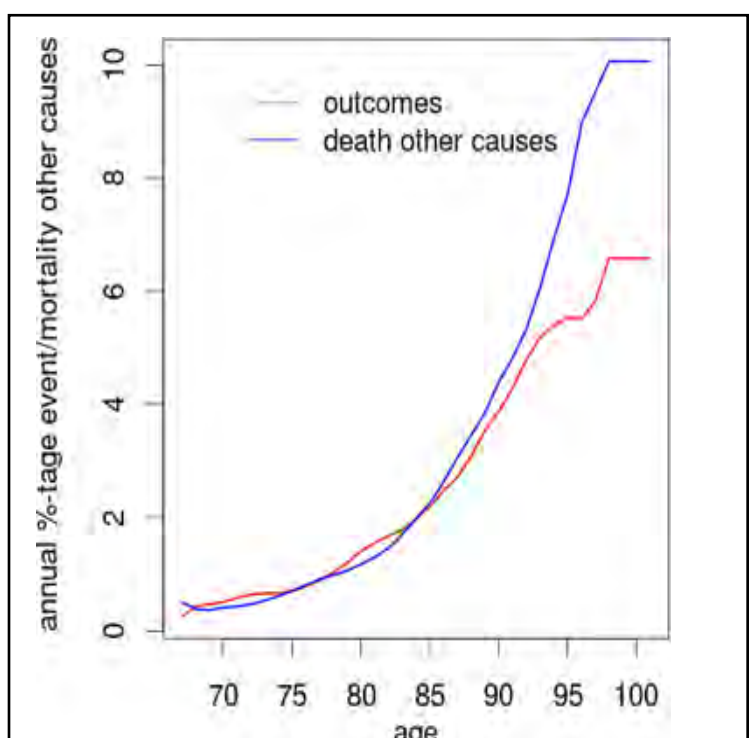

Figure 7. Observed event and censoring rates in the combined *WHISH* arms.

Primary Outcome:

1. We combined all outcomes data from both arms of the *WHISH* trial as of the time of the last DSMB meeting (Fall 2018).
2. Using this data, for each one-year interval, we computed that, within the *WHISH* cohort, the probability that a participant, who was age X at the beginning of the year, during that year will get either the primary CVD outcome of MI, stroke, or cardiovascular death, or the censoring outcome of non-cardiovascular death (acknowledging computing risks). These rates are averaged over three-year

overlapping intervals for smoothing (e.g., to estimate the rate for 78-year-olds, we averaged the observed rate of 77-year-olds, 78-year-olds, and 79- year-olds), and we kept the rate constant for the over-98-year-olds. This yielded the rates shown in Figure 7 of the Research Plan, which is reproduced here.

3. Using these observed rates, and the actual ages of the randomized women, we generated 100,000 data sets with various effect sizes and various lengths of follow-up, noting that for an effect size of x%, we assume that the comparison arm has rate (100+x/2)%, and the intervention arm has (100-x/2)%, as the rates in Figure 7 combine both arms.

4. We then used Cox models, as described above, to test (at level 0.05) whether the simulated results yielded a significant effect. The number of significant results yield the power curves of Figure 8 of the Research Plan, also reproduced here.

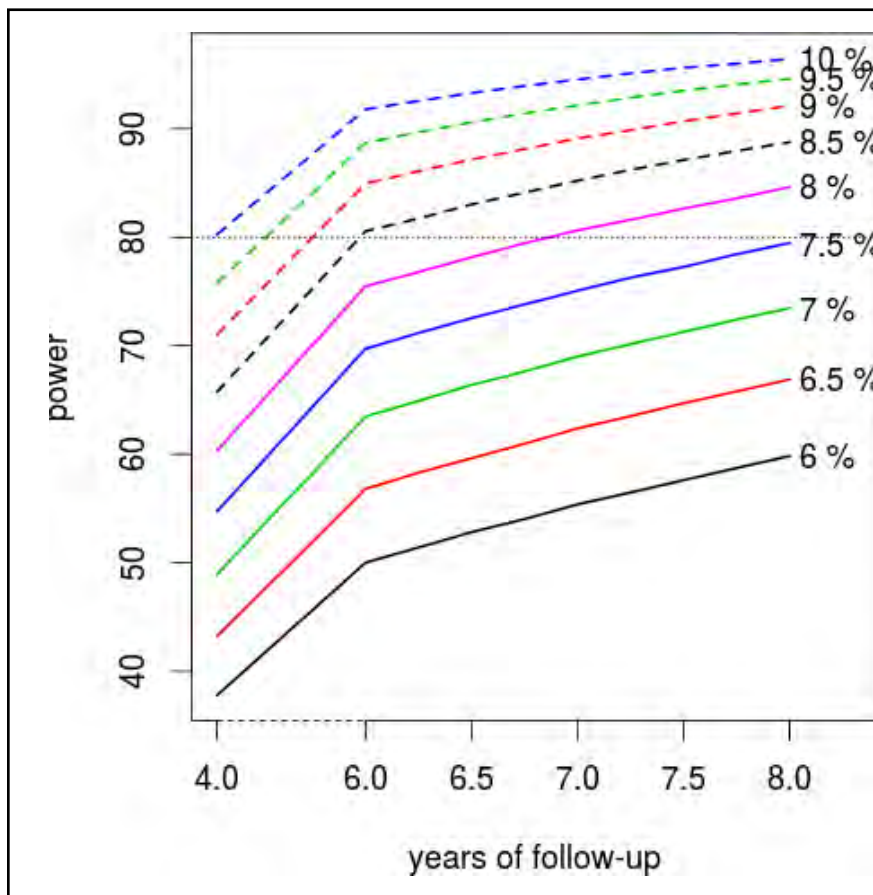

Figure 8. Estimated power for the *WHISH* primary endpoint as a function of the observed effect size of physical activity.

Our conclusion was that with an effect size of about 8.5% we have 85- 90% power to observe significant results after 8 years of follow-up. The effect size of 8.5% is consistent with the effect that we observed in walking (see Section C.d of the Research Plan). The effect size of walking has been consistently increasing over the length of the trial.

Additional Outcomes. The additional outcomes for which we provide power calculations are the Rand-36 Physical Function outcome, the Short Physical Performance Battery (SPPB), the WHI Insomnia Rating Scale (WHIIRS), and the Depressive Systems Scale (CES-D). These outcomes would only be tested after the primary outcome has been evaluated. As described in the Research Plan, we divide the type 1 error into 0.05/2 for the Rand-36 scale, and 0.05/6 for the remaining three outcomes, so that the combined type 1 error for the additional outcomes is controlled by 0.05. Other than the SPPB, these outcomes are evaluated among all the surviving participants at the end of the study (estimated to be ~36,000); the SPPB is measured in 5,000 participants who are part of the LLS2. For the Rand-36 and SPPB we have good estimates for the standard deviation, for the other two outcomes, we provide the power in terms of SDs. The power was shown in Table 8 of the Research Plan, reproduced here.

Table 8. Detectable differences for the additional outcomes measures of the *WHISH* trial with 8% and 90% power. The type I error for the Rand-36 outcome is .05/2, the type 1 error for the other three outcomes is .05/6.

	SD	Differences detectable with	
		80% power	90% power
Rand-36	26	0.85	0.97
WHIIRS, CES-D	Differences in SDs of measure	0.039	0.042
SPPB	2.4	0.26	0.29

Biomarker Pilot (Aim 3). The biomarker Aim 3 is intended to be a pilot study for future projects. As described in the Research Plan, the actual biomarkers and study design are not yet determined. As such, we do not provide power estimates – in line with the goal of this aim, to provide power estimates for future studies.

Conditional Power Analyses. Please see 3.3.3. Interim Analysis for the WHISH Trial to be taking place in 2022.

SECTION 5 - OTHER CLINICAL TRIAL-RELATED INFORMATION

5.1 Project Management Plan

Because *WHISH* began in 2015 and is underway, recruitment is complete. In addition, the intervention program is underway and retention rates for return of behavior change and outcomes forms is exceptionally high. Therefore, our trial benchmarks will focus on continuing this record of rigorous trial implementation through this trial continuation period.

The project management team includes the three multiple PIs, Dr. Charles Kooperberg, Dr. Marcia Stefanick and Dr. Andrea LaCroix. Refer to Section 3.5 for the project oversight responsibilities. The team will be responsible for administrative functions of the project in addition to the scientific functions. Drs. Kooperberg, Stefanick and LaCroix each bring extensive knowledge and necessary expertise in the creation, management and analysis, as well as the commitment to make major decisions on the project via consensus. Dr. Kooperberg will be designated the contact PI at FHCRC. He will be responsible for communications with the CCC investigator team at Stanford, including regular (approximately monthly) project conferences that will also include Dr. LaCroix and Dr. Stefanick. Except for Dr. Kooperberg, all other PIs will be blinded to the data and data analytic activities. Decisions regarding the project management to meet the scientific objectives will be made jointly by the PIs with the exception of Dr. Kooperberg, who will not be a part of making decisions regarding the intervention component of the study. Drs. Stefanick, LaCroix, and LaMonte will all take active roles in unblinded primary data analyses under the project specific aims during the entirety of the blinded intervention phase. All PIs will take an active role in ongoing evaluation of data quality and manuscript writing; and will provide support in all aspects of study administration and scientific dissemination. Staff and investigators will meet regularly, and as needed, to address questions, review progress, and manage the work at-hand. Drs. Kooperberg and LaCroix will share responsibility for communicating with the WHI. A project management timeline with and milestones pre- milestone benchmarks will be developed and monitored at each conference call. The pre-milestone benchmarks will be used to determine if contingency plans need to be put into place. The team is confident of meeting progress goals and budget based on their individual and combined history of meeting these goals.

The team has worked together successfully for the past four years with *WHISH* and has demonstrated early attentiveness to and resolution of all study aspects, including budget, monitoring and attending to corrective responses, meeting all reporting timelines. The multi-PI team meet annually with the WHI DSMB, which oversees *WHISH,* to monitor safety and give advice about the trial continuing.

Decisions will be made by consensus. In the event of not reaching consensus, Dr. Charles Kooperberg will decide.

Dissemination of *WHISH* trial data and results is described under the Dissemination Plan (Section 4.7) and will be executed in accordance with the WHI data sharing plan established with the NHLBI Program Office.

The *WHISH* study protocol and standard WHI operating procedures for data collection are followed. The protocol is reviewed periodically and revisions made as needed.

5.2 Single Site Justification Plan

The *WHISH* trial is conducted primarily by mail with no participant clinic visits. The nature of the trial lends itself to be enrolled through a single site. All *WHISH* participants were enrolled and randomized out of the Women's Health Initiative (WHI) Clinical Coordinating Center (CCC) at the Fred Hutchinson Cancer Research Center in Seattle, WA, although the participants themselves span across the United States and are associated with one of four WHI regional centers. *WHISH* is a novel and pragmatic clinical trial for its use of a randomized Zelen consent design. Women were pre-screened for eligibility based on previously collected WHI data (ambulatory, enrolled in the WHI Medical Record Cohort, or in

fee-for-service Medicare with CMS data). A total of 49,331 WHI participants were randomized with 24,674 to usual-activity comparison and 24,659 to physical activity (PA) intervention. The *WHISH* comparison participants receive their usual WHI contact through mailings but, as part of the *WHISH* pragmatic design, do not receive materials from *WHISH*. Women randomized to intervention receive materials, have access to an intervention website, and have the opportunity for telephone contact through Stanford University. Enrollment in the *WHISH* trial was completed as planned in 2015.

An Accelerometry Substudy within *WHISH* objectively monitors changes in PA in the intervention and comparison groups through accelerometry wear. The substudy invited, consented, and ultimately enrolled 2,350 *WHISH* participants (1,182 [50%] comparison, 1,168 [50%] intervention) within the allotted timeline. Deployment of the substudy among enrolled participants occurred through a single site. The accelerometers were delivered by mail with written instructions. Experienced staff contacted participants just before accelerometers were mailed as well as during the 7-week wear period as support for providing instructions. This substudy is directed by the *WHISH* team at the WHI-CCC and conducted by the University of California, San Diego (UCSD) Exercise and Physical Activity Resource Center (EPARC). During the proposed trial period, approximately 2,000 *WHISH* participants will be recruited to wear an accelerometer during one 7- day/night time period. Recruitment primarily will occur among participants who have successfully completed the initial *WHISH* accelerometer wearings and supplemented as needed by newly recruited *WHISH* participants. We plan to conduct the Accelerometry Substudy during the first year of the *WHISH* trial. Based on our past experience, we expect to successfully complete recruitment in the allotted timeframe.

NHLBI has classified *WHISH* to be a single-site study due to the centralized pragmatic nature of the trial structure, including enrollment, intervention and follow-up. There are no clinical sites managing participant intervention or follow-up.

5.3. Dissemination Plan

A thoughtful and broad-based plan to disseminate *WHISH* trial findings is critical to ensuring that the scientific research and public health community, policy makers, research study participants, and the general public are informed of the results, particularly as they pertain to health benefits and risks of increasing physical activity and decreasing sedentary behavior in older women in the U.S. We will use a diverse portfolio of strategies, including: (a) media coverage through press releases and interviews targeted to local and national newspapers, television and radio outlets; (b) production of the research summary document and fact sheet targeted to the general public, which clearly and concisely summarizes the key conclusions of the trial; (c) production of professionally designed flyers, posters, brochures, and research briefs targeted to broad audiences; (d) study newsletters targeted to study participants who were randomized to the intervention arm; (e) distribution of dissemination materials to community agencies, professional societies, and health-related websites and list-serves; (f) hosting and attending seminars, conferences, community forums and health fairs; and (g) mailing of personal thank-you letters to research study participants. The *WHISH* Dissemination Plan also includes: 1) Full support and compliance with the data sharing policies of NHLBI; 2) Rapid publication of initial trial results after year 8, during the NCE; 3) Simultaneous posting of results to ClinicalTrials.gov (NCT02425345); and 4) Use of a data sharing plan that follows policies established for other WHI core studies. Analytic datasets will be made available to the WHI investigator community after the data have been prepared and documented for distribution and the main trial outcome paper has been published, typically within 12 months of trial closure. Any investigator (in the US or internationally) can request use of the data by submitting a paper proposal to the WHI Publications & Presentations (P&P) committee. The data will also be submitted to the NHLBI Project Office on a pre-specified schedule and made available to the public through limited release datasets. In addition, if the *WHISH* Intervention is found to be beneficial, we will develop a plan and seek additional resources to package and disseminate this unique and timely resource to the American public on a broad scale.

SECTION 6 - CLINICAL TRIAL MILESTONE PLAN

6.1. Study Primary Completion Date: 12/31/24 Anticipated

6.2. Study Final Completion Date: 12/31/24 Anticipated

6.3. Enrollment and randomization Enrollment of the First Participant:

(Study Start Date) 4/2/15 Actual

6.3.1. Milestones for Protocol Phase 2

- Year 01 - Milestone 1: Submission of a revised protocol to NHLBI for transmission to DSMB for review/approval in 2020
- Year 01 - Milestone 2: Submission of a plan to NHLBI by early September 2020 for the conduct of the interim analysis on the primary CVD measure, to occur no later than July 1, 2022.
- Year 01- Milestone 3: Submission of IRB approval
- Year 01 - Milestone 4: Submission of an interim progress report by the 9th month to transition to the R33.
- Year 01- Milestone 5: Send quarterly reports of the number of cases (observed and expected) month 3, via e-connect
- Year 01 -Milestone 6: Send quarterly reports of the number of cases (observed and expected) in month 6, via e-connect
- Year 01 - Milestone 7: Send quarterly reports of the number of cases (observed and expected) in month 9, via e-connect

6.4. Completion of primary endpoint data analyses

6.5. Reporting of results in ClinicalTrials.gov

6.6. Is this an applicable clinical trial under FDAAA? No

7. REFERENCES (Phase 2; numbering continued from Phase 1)

28. U.S. Department of Health and Human Services. 2008 Physical Activity Guidelines for Americans. Washington, DC: U.S. Department of Health and Human Services. 2008.

29. U.S. Department of Health and Human Services. 2018 Physical Activity Guidelines for Americans, 2nd edition. Washington, DC: U.S. Department of Health and Human Services. 2018.

30. Bandura A. Social cognitive theory: an agentic perspective. Annu Rev Psychol. 2001;52:1-26.

31. Marshall SJ and Biddle SJ. The transtheoretical model of behavior change: a meta-analysis of applications to physical activity and exercise. Ann Behav Med. 2001;23:229-246.

32. Ryan RM and Deci EL. Self-determination theory and the facilitation of intrinsic motivation, social development, and well-being. Am Psychol. 2000;55:68-78.

33. MacNab M, Lee SH, McCloughan L, Hanley J, McKinstry B and Pinnock H. Oximetry-supported self-management for chronic obstructive pulmonary disease: mixed method feasibility pilot project. BMC Health Serv Res. 2015;15:485. PMC4624181.

34. NIA. Go4Life ® from the National Institute on Aging at NIH. (http://go4life.nia.nih.gov/). Accessed May 27, 2019.

35. King AC, Hekler EB, Grieco LA, Winter SJ, Sheats JL, Buman MP, Banerjee B, Robinson TN and Cirimele J. Harnessing different motivational frames via mobile phones to promote daily physical activity and reduce sedentary behavior in aging adults. *PLoS One*. 2013;8:e62613. PMC3636222.

36. Prentice RL, Mossavar-Rahmani Y, Huang Y, Van Horn L, Beresford SA, Caan B, Tinker L, Schoeller D, Bingham S, Eaton CB, Thomson C, Johnson KC, Ockene J, Sarto G, Heiss G, Neuhouser ML. Evaluation and comparison of food records, recalls, and frequencies for energy and protein assessment by using recovery biomarkers. Am J Epidemiol. 2011;174(5):591-603. PMCID: PMC3202154.

37. Hays RD, Sherbourne CD and Mazel RM. The RAND 36-Item Health Survey 1.0. Health Econ. 1993;2:217-227.

38. Rikli RE and Jones CJ. Development and validation of criterion-referenced clinically relevant fitness standards for maintaining physical independence in later years. Gerontologist. 2013;53:255-267.

39. Althoff T, Sosic R, Hicks JL, King AC, Delp SL and Leskovec J. Large-scale physical activity data reveal worldwide activity inequality. England: Springer Science and Business Media LLC Nature (London), 2017;547(7663):336-339.

40. King AC, Hekler EB, Grieco LA, Winter SJ, Sheats JL, Buman MP, Banerjee B, Robinson TN and Cirimele J. Effects of Three Motivationally Targeted Mobile Device Applications on Initial Physical Activity and Sedentary Behavior Change in Midlife and Older Adults: A Randomized Trial. PLoS One. 2016;11:e0156370. PMC4924838.

Appendix B. WHISH Trial Design CONSORT

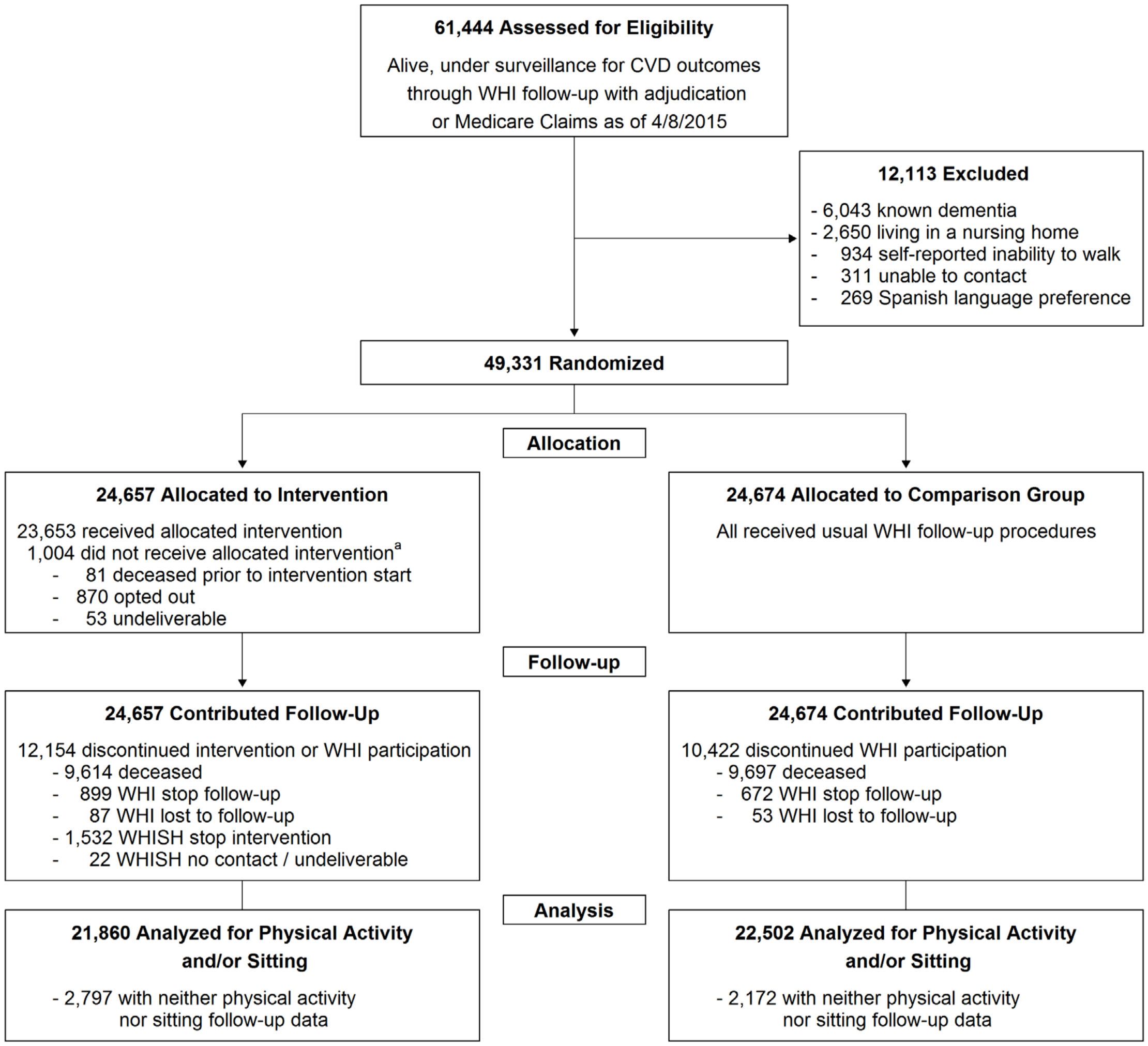

Alt-Text: a CONSORT diagram for the WHISH trial. 61,444 potential participants were assessed for eligibility, of which 49,331 were randomized. Of the 24,65757 participants allocated to the intervention, all 24,657 contributed follow-up, and 21,860 were analyzed for physical activity and/or sitting outcomes. Of the 24,674 participants allocated to the comparison group, all 24,674 contributed follow-up, and 22,502 were analyzed for physical activity and/or sitting outcomes.

WOMEN'S HEALTH INITIATIVE

Form 521 - Physical Activity Questionnaire

Ver. 1

MARKING INSTRUCTIONS

- You may use a pencil or pen (blue or black only).
- To change an answer:
  - If you are using a pencil, cleanly erase the incorrect mark.
  - If you are using a pen, mark an X through the incorrect mark.
- Please do not make any stray marks on this form.

CORRECT MARK

INCORRECT MARKS

OFFICE USE ONLY

Date Received: ___ - ___ - ___
Month Day Year

○ FCA ○ OU1 ○ OU2

AFFIX LABEL HERE

These questions ask about physical activity and other habits that may affect your health. Please answer each question as accurately as possible. There are no right or wrong answers.

The first question is about your walking.

1. Think about the walking you do outside the home. How often do you walk outside the home for more than 10 minutes without stopping? **Mark only one.**

○0 Rarely or never → **Go to the next page.**

○1 1 to 3 times each month
○2 1 time each week
○3 2 to 3 times each week
○4 4 to 6 times each week
○5 7 or more times each week

→ **Go to Question 1.1 below.**

When you walk outside the home for more than 10 minutes without stopping,

1.1 For how many minutes do you usually walk?

○1 Less than 20 minutes
○2 20 to 39 minutes
○3 40 to 59 minutes
○4 1 hour or more

1.2 What is your usual speed?

○2 Casual strolling or walking (less than 2 miles an hour)
○3 Average or normal (2-3 miles an hour)
○4 Fairly fast (3-4 miles an hour)
○5 Very fast (more than 4 miles an hour)
○9 Don't know

00303

PLEASE MAKE NO MARKS IN THIS AREA

R:\DOC\EXT2015\WHI\F521.DOC 3/25/15

Form 521 - Physical Activity Questionnaire

2. When you exercise or walk in your usual fashion how would you rate your level of exertion (degree of effort)? **Mark only one.**

Nothing at all	Very, very weak (just noticeable)	Very weak	Weak	Moderate	Somewhat strong	Strong (heavy)	Very strong	Very, very strong (almost maximal)	Maximal
○ 0	○ 1	○ 2	○ 3	○ 4	○ 5	○ 6	○ 7	○ 8	○ 9

3. Are you able to walk at a normal pace for a half hour (30 minutes) or more?

○ 0 No ○ 1 Yes

4. Are you able to walk slowly for a half hour (30 minutes) or more?

○ 0 No ○ 1 Yes

5. During a usual day and night, about how many hours do you spend sitting? Be sure to include the time you spend sitting at work, sitting at the table eating, driving or riding in a car or bus, and sitting up watching TV or talking.

Less than 4 hours	4-5 hours	6-7 hours	8-9 hours	10-11 hours	12-13 hours	14-15 hours	16 or more hours
○ 1	○ 2	○ 3	○ 4	○ 5	○ 6	○ 7	○ 8

6. During a usual day and night, about how many hours do you spend sleeping or lying down with your feet up? Be sure to include the time you spend sleeping or trying to sleep at night, resting or napping, and lying down watching TV.

Less than 4 hours	4-5 hours	6-7 hours	8-9 hours	10-11 hours	12-13 hours	14-15 hours	16 or more hours
○ 1	○ 2	○ 3	○ 4	○ 5	○ 6	○ 7	○ 8

Go to the next page. →

7. On a typical **DAY**, how much time do you spend (from when you wake up until you go to bed) doing the following? **Mark only one answer per question.**

	None	15 min. or less	30 min.	1 hour	2 hours	3 hours	4 hours	5 hours	6 hours or more
7.1 Sitting while watching television (including videos on VCR/DVD).	○1	○2	○3	○4	○5	○6	○7	○10	○11
7.2 Sitting while using the computer for non-work activities or playing video games.	○1	○2	○3	○4	○5	○6	○7	○10	○11
7.3 Sitting while doing non-computer office work or paperwork not related to your job (paying bills, etc.).	○1	○2	○3	○4	○5	○6	○7	○10	○11
7.4 Sitting listening to music, reading a book or magazine, or doing arts and crafts.	○1	○2	○3	○4	○5	○6	○7	○10	○11
7.5 Sitting and talking on the phone or texting.	○1	○2	○3	○4	○5	○6	○7	○10	○11
7.6 Sitting in a car, bus, train, or other mode of transportation.	○1	○2	○3	○4	○5	○6	○7	○10	○11

Go to the next page. →

PLEASE MAKE NO MARKS IN THIS AREA 00303

8. If you fell when you are away from your home, how confident are you that someone would be able to quickly help you?

- ○ 1 Not at all confident
- ○ 2 Somewhat confident
- ○ 3 Very confident
- ○ 9 Don't know/not sure

9. Have you had a fall in the **past 12 months**? By a "fall", we mean
- Fell all the way to the floor or the ground, or
- Fell and hit an object like a chair or stair

- ○ 0 No → **Go to page 6.**
- ○ 1 Yes

10. How many times have you fallen in the **past 12 months**? (If you are unsure, make your best guess.)

- ○ 1 One time
- ○ 2 Two or three times
- ○ 3 Four or five times
- ○ 4 Six or more times

Go to the next page. →

11. At the time of your most recent fall, were you:

	No	Yes
11.1 Walking outside the home?	○ 0	○ 1
11.2 Doing strenuous exercise (but not walking outside the home)? Strenuous means you work up a sweat and would be exhausted by prolonged participation. For example, aerobics, aerobic dancing, jogging, tennis, swimming laps.	○ 0	○ 1
11.3 Doing moderate exercise (but not walking outside the home)? Moderate means exercise that is not exhausting. For example, biking outdoors, using an exercise machine (like a stationary bike or treadmill), calisthenics, easy swimming, popular and folk dancing.	○ 0	○ 1
11.4 Doing mild exercise? For example, slow dancing, bowling, or golf.	○ 0	○ 1
11.5 Doing other exercise (not previously listed)?	○ 0	○ 1
11.6 Doing strenuous indoor household chores (such as scrubbing floors, sweeping, or vacuuming)?	○ 0	○ 1
11.7 Working in the yard (such as mowing, raking, gardening, or shoveling snow)?	○ 0	○ 1

12. Were you injured as a result of any fall in the **past 12 months**?

○ 0 No

○ 1 Yes →

12.1 Please indicate what types of injuries. **Mark all that apply.**

○ 1 Fracture

○ 2 Laceration/cut

○ 3 Bruising

○ 4 Sprained or strained joint (wrist, knee, ankle, etc.)

○ 5 Other injury (Please specify:________________________________)

12.2 Did you injure your head?

○ 0 No

○ 1 Yes

Go to the next page. →

Form 521 - Physical Activity Questionnaire

This set of questions is to help us understand the full range of activities you are doing. These questions are about activities that you may have done in the past 4 weeks. The questions on the following pages are similar to the example shown below.

If you **DID NOT** do the activity:

- Mark **NO** and move to the next question.

If you **DID** the activity in the past 4 weeks:

Step #1 Mark **YES**.

Step #2 Think about how many TIMES a week you usually did it and mark one answer.

Step #3 Mark one answer to show the TOTAL HOURS in a typical week you did the activity.

Here is an example of how Mrs. Jones would answer the first question: Mrs. Jones usually visits her friends Maria and Olga twice a week. She usually spends one hour on Monday with Maria and two hours on Wednesday with Olga. Therefore, the total hours a week that she visits with friends is 3 hours a week.

**EXAMPLE** In a typical week during the past 4 weeks, did you…	No	Yes	How many TIMES a week? Think in terms of days				How many TOTAL hours a week did you usually do it?					
			1	2	3-4	5 or more	Less than 1 hour	1-2.5 hours	3-4.5 hours	5-6.5 hours	7-8.5 hours	9 or more hours
Visit with friends or family (other than those you live with)?	○0	●1	○1	●2	○3	○4	○1	○2	●3	○4	○5	○6

**13. In a typical week during the past 4 weeks, did you…**	No	Yes	How many TIMES a week? Think in terms of days				How many TOTAL hours a week did you usually do it?					
			1	2	3-4	5 or more	Less than 1 hour	1-2.5 hours	3-4.5 hours	5-6.5 hours	7-8.5 hours	9 or more hours
13.1 Jog or run (including use of treadmill)?	○0	○1	○1	○2	○3	○4	○1	○2	○3	○4	○5	○6

Go to the next page. →

In a typical week during the past 4 weeks, did you…	No	Yes	How many TIMES a week? Think in terms of days				How many TOTAL hours a week did you usually do it?					
			1	2	3-4	5 or more	Less than 1 hour	1-2.5 hours	3-4.5 hours	5-6.5 hours	7-8.5 hours	9 or more hours
13.2 Walk uphill or hike uphill (count only uphill part; include use of treadmill)?	○0	○1	○1	○2	○3	○4	○1	○2	○3	○4	○5	○6
13.3 Walk fast or briskly for exercise (do not count walking leisurely or uphill; include use of treadmill)?	○0	○1	○1	○2	○3	○4	○1	○2	○3	○4	○5	○6
13.4 Walk to do errands (such as to/from a store or to take children to school (count walk time only)?	○0	○1	○1	○2	○3	○4	○1	○2	○3	○4	○5	○6
13.5 Walk leisurely for exercise or pleasure?	○0	○1	○1	○2	○3	○4	○1	○2	○3	○4	○5	○6
13.6 Ride a bicycle or stationary cycle?	○0	○1	○1	○2	○3	○4	○1	○2	○3	○4	○5	○6
13.7 Do other aerobic machines such as rowing, or step machines (do not count treadmill or stationary cycle)?	○0	○1	○1	○2	○3	○4	○1	○2	○3	○4	○5	○6
13.8 Do water exercises (do not count other swimming)?	○0	○1	○1	○2	○3	○4	○1	○2	○3	○4	○5	○6

Go to the next page. →

Form 521 - Physical Activity Questionnaire

In a typical week during the past 4 weeks, did you…	No	Yes	How many TIMES a week? Think in terms of days				How many TOTAL hours a week did you usually do it?					
			1	2	3-4	5 or more	Less than 1 hour	1-2.5 hours	3-4.5 hours	5-6.5 hours	7-8.5 hours	9 or more hours
13.9 Swim moderately or fast?	○ 0	○ 1	○ 1	○ 2	○ 3	○ 4	○ 1	○ 2	○ 3	○ 4	○ 5	○ 6
13.10 Swim gently?	○ 0	○ 1	○ 1	○ 2	○ 3	○ 4	○ 1	○ 2	○ 3	○ 4	○ 5	○ 6
13.11 Do stretching or flexibility exercises (do not count yoga or Tai-chi)?	○ 0	○ 1	○ 1	○ 2	○ 3	○ 4	○ 1	○ 2	○ 3	○ 4	○ 5	○ 6
13.12 Do yoga or Tai-chi?	○ 0	○ 1	○ 1	○ 2	○ 3	○ 4	○ 1	○ 2	○ 3	○ 4	○ 5	○ 6
13.13 Do aerobics or aerobic dancing?	○ 0	○ 1	○ 1	○ 2	○ 3	○ 4	○ 1	○ 2	○ 3	○ 4	○ 5	○ 6
13.14 Do moderate to heavy strength training (such as hand-held weights of more than 5 lbs., weight machines, or push-ups)?	○ 0	○ 1	○ 1	○ 2	○ 3	○ 4	○ 1	○ 2	○ 3	○ 4	○ 5	○ 6
13.15 Do light strength training (such as hand-held weights of 5 lbs. or less or elastic bands)?	○ 0	○ 1	○ 1	○ 2	○ 3	○ 4	○ 1	○ 2	○ 3	○ 4	○ 5	○ 6
13.16 Do general conditioning exercises, such as light calisthenics or chair exercises (do not count strength training)?	○ 0	○ 1	○ 1	○ 2	○ 3	○ 4	○ 1	○ 2	○ 3	○ 4	○ 5	○ 6

Thank you for completing this questionnaire.
Please take a moment to review any questions you may have missed.

PLEASE MAKE NO MARKS IN THIS AREA

00303

296362-1 / 1 / 2 /

WOMEN'S HEALTH INITIATIVE

Form 521 - Physical Activity Questionnaire

Ver. 2

MARKING INSTRUCTIONS

- You may use a pencil or pen (blue or black only).
- To change an answer:
  - If you are using a pencil, cleanly erase the incorrect mark.
  - If you are using a pen, mark an X through the incorrect mark.
- Please do not make any stray marks on this form.

CORRECT MARK

○ ● ○ ○

INCORRECT MARKS

OFFICE USE ONLY	- Affix label here-
Date Received: ___-___-___ (MM/DD/YY)	Participant ID: __ __ __ __ - __ __ __ - __
☐FCA ☐OUI ☐OU2	First Name __________ M.I.____
	Last Name __________

These questions ask about physical activity, habits, and feelings that may affect your health. Please answer each question as accurately as possible. There are no right or wrong answers.

1. Think about the walking you do outside the home. How often do you walk outside the home for more than 10 minutes without stopping? **Mark only one.**

○0 Rarely or never → **Go to Question 2.**

○1 1 to 3 times each month
○2 1 time each week
○3 2 to 3 times each week
○4 4 to 6 times each week
○5 7 or more times each week

→ **Go to Question 1.1 below.**

When you walk outside the home for more than 10 minutes without stopping,

1.1 For how many minutes do you usually walk?

○1 Less than 20 minutes
○2 20 to 39 minutes
○3 40 to 59 minutes
○4 1 hour or more

1.2 What is your usual speed?

○2 Casual strolling or walking (less than 2 miles an hour)
○3 Average or normal (2-3 miles an hour)
○4 Fairly fast (3-4 miles an hour)
○5 Very fast (more than 4 miles an hour)
○9 Don't know

013204

This question is about walking intentionally for at least 5 minutes. Think about how often you intentionally walked for at least 5 minutes without stopping. The walking could be outdoors or indoors.

**2.** In a typical week during the past 4 weeks, did you…	No	Yes	**2.1** How many times per day? 1	2	3-4	5 or more	**2.2** How many days per week? 1-2	3-4	5	6-7
Walk for at least 5 minutes?	○0	○1	○1	○2	○3	○4	○1	○2	○3	○4

This next question is about your walking in general.

3. How many days per week, in general, do you walk outside for any reason or any amount of time?

0 days	1 day	2 days	3 days	4 days	5 days	6 days	7 days
○0	○1	○2	○3	○4	○5	○6	○7

This next set of questions are about activities that you may have done in the past 4 weeks. The questions are similar to the example below.

If you did not do the activity: Mark "No" and move to the next question.

If you did the activity in the past 4 weeks:
- Step #1 Mark "Yes".
- Step #2 Think about how many times a week you usually did it and mark one answer.
- Step #3 Mark one answer for the total hours in a typical week you did the activity.

Example: Mrs. Jones usually visits her friends Maria and Olga twice a week. She spends 1 hour on Monday with Maria and 2 hours on Wednesday with Olga. The total hours a week that she visits with friends is 3 hours.

EXAMPLE

In a typical week during the past 4 weeks, did you…	No	Yes	How many times a week? 1	2	3-4	5 or more	How many total hours a week did you usually do it? Less than 1 hour	1-2.5 hours	3-4.5 hours	5-6.5 hours	7-8.5 hours	9 or more hours
Visit with friends or family (other than those you live with)?	○0	●1	○1	●2	○3	○4	○1	○2	●3	○4	○5	○6

**4.** In a typical week during the past 4 weeks, did you…	No	Yes	How many times a week?				How many total hours a week did you usually do it?					
			1	2	3-4	5 or more	Less than 1 hour	1-2.5 hours	3-4.5 hours	5-6.5 hours	7-8.5 hours	9 or more hours
**4.1** Jog or run (including use of treadmill)?	○0	○1	○1	○2	○3	○4	○1	○2	○3	○4	○5	○6
**4.2** Walk uphill or hike uphill (count only uphill part; include use of treadmill)?	○0	○1	○1	○2	○3	○4	○1	○2	○3	○4	○5	○6
**4.3** Walk fast or briskly for exercise (do not count walking leisurely or uphill; include use of treadmill)?	○0	○1	○1	○2	○3	○4	○1	○2	○3	○4	○5	○6
**4.4** Walk to do errands, such as to/from a store (count walk time only)?	○0	○1	○1	○2	○3	○4	○1	○2	○3	○4	○5	○6
**4.5** Walk leisurely for exercise or pleasure?	○0	○1	○1	○2	○3	○4	○1	○2	○3	○4	○5	○6
**4.6** Ride a bicycle or stationary cycle?	○0	○1	○1	○2	○3	○4	○1	○2	○3	○4	○5	○6
**4.7** Do other aerobic machines such as rowing, or step machines (do not count treadmill or stationary cycle)?	○0	○1	○1	○2	○3	○4	○1	○2	○3	○4	○5	○6
**4.8** Do water exercises (do not count swimming)?	○0	○1	○1	○2	○3	○4	○1	○2	○3	○4	○5	○6
**4.9** Swim moderately or fast?	○0	○1	○1	○2	○3	○4	○1	○2	○3	○4	○5	○6
**4.10** Swim gently?	○0	○1	○1	○2	○3	○4	○1	○2	○3	○4	○5	○6
**4.11** Do stretching or flexibility exercises (do not count yoga or Tai-chi)?	○0	○1	○1	○2	○3	○4	○1	○2	○3	○4	○5	○6
**4.12** Do yoga or Tai-chi?	○0	○1	○1	○2	○3	○4	○1	○2	○3	○4	○5	○6

013204

In a typical week during the past 4 weeks, did you…	No	Yes	How many times a week? 1	2	3-4	5 or more	How many total hours a week did you usually do it? Less than 1 hour	1-2.5 hours	3-4.5 hours	5-6.5 hours	7-8.5 hours	9 or more hours
**4.13** Do aerobics or aerobic dancing?	O0	O1	O1	O2	O3	O4	O1	O2	O3	O4	O5	O6
**4.14** Do moderate to heavy strength training, such as hand-held weights of more than 5 lbs., weight machines, or push-ups?	O0	O1	O1	O2	O3	O4	O1	O2	O3	O4	O5	O6
**4.15** Do light strength training, such as hand-held weights of 5 lbs. or less or elastic bands?	O0	O1	O1	O2	O3	O4	O1	O2	O3	O4	O5	O6
**4.16** Do general conditioning exercises, such as light calisthenics or chair exercises (do not count strength training)?	O0	O1	O1	O2	O3	O4	O1	O2	O3	O4	O5	O6

The next question is about the degree of effort you feel when walking or exercising.

5. When you exercise or walk in your usual fashion how would you rate your level of exertion (degree of effort)? **Mark only one.**

Nothing at all	Very, very weak (just noticeable)	Very weak	Weak	Moderate	Somewhat strong	Strong (heavy)	Very strong	Very, very strong (almost maximal)	Maximal
O0	O1	O2	O3	O4	O5	O6	O7	O8	O9

Please respond to the next 3 questions about where you live.

6. There are major barriers to walking in my local area that make it hard to get from place to place.

Strongly disagree	Somewhat disagree	Somewhat agree	Strongly agree
O1	O2	O3	O4

7. There are many places to go within easy walking distance of my home.

Strongly disagree	Somewhat disagree	Somewhat agree	Strongly agree
○ 1	○ 2	○ 3	○ 4

8. Are there places that you can walk to that help you relax, clear your mind, and reduce stress?

○ 0 No ○ 2 Prefer not to answer
○ 1 Yes ○ 9 Don't know

These questions are about sitting and lying down.

9. During a usual day and night, about how many hours do you spend sitting? Be sure to include the time you spend sitting at work, sitting at the table eating, driving or riding in a car or bus, and sitting up watching TV or talking. **Mark only one.**

Less than 4 hours	4-5 hours	6-7 hours	8-9 hours	10-11 hours	12-13 hours	14-15 hours	16 or more hours
○ 1	○ 2	○ 3	○ 4	○ 5	○ 6	○ 7	○ 8

10. Everybody sits throughout the day, but they do so in different patterns. Some people often sit for very long periods of time without getting up. Other people frequently get up and sit back down. Think about your sitting pattern. **Mark only one.**

Where do you fall on a scale from 0 to 10?

○ 0 ○ 1 ○ 2 ○ 3 ○ 4 ○ 5 ○ 6 ○ 7 ○ 8 ○ 9 ○ 10

Sit for very long periods without getting up — Frequently get up and sit down

11. During a usual day and night, about how many hours do you spend sleeping or lying down with your feet up? Be sure to include the time you spend sleeping or trying to sleep at night, resting or napping, and lying down watching TV. **Mark only one.**

Less than 4 hours	4-5 hours	6-7 hours	8-9 hours	10-11 hours	12-13 hours	14-15 hours	16 or more hours
○ 1	○ 2	○ 3	○ 4	○ 5	○ 6	○ 7	○ 8

These next questions are about your sleep habits. Please mark one of the answers for each of the following questions. Pick the answer that best describes how often you experienced the situation in the past 4 weeks.

		No, not in the past 4 weeks	Yes, less than once a week	Yes, 1 or 2 times a week	Yes, 3 or 4 times a week	Yes, 5 or more times a week
**12.**	Did you have trouble falling asleep?	○ 1	○ 2	○ 3	○ 4	○ 5
**13.**	Did you wake up several times at night?	○ 1	○ 2	○ 3	○ 4	○ 5
**14.**	Did you wake up earlier than you planned to?	○ 1	○ 2	○ 3	○ 4	○ 5
**15.**	Did you have trouble getting back to sleep after you woke up too early?	○ 1	○ 2	○ 3	○ 4	○ 5

16. Overall, was your typical night's sleep during the past 4 weeks:

Very sound or restful	Sound or restful	Average quality	Restless	Very restless
○ 1	○ 2	○ 3	○ 4	○ 5

These questions ask about falls you might have had.

17. Have you had a fall in the past 12 months? By a "fall", we mean

- Fell all the way to the floor or the ground, or
- Fell and hit an object like a chair or stair

○ 0 No ⟶ **Go to Question 20.** ○ 1 Yes ⟶ **Go to Question 18.**

**18.**	**At the time of your most recent fall, were you:**	No	Yes
**18.1**	Walking outside the home?	○ 0	○ 1
**18.2**	Doing strenuous exercise (but not walking outside the home)? Strenuous means you work up a sweat and would be exhausted by prolonged participation. For example, aerobics, aerobic dancing, jogging, tennis, swimming laps.	○ 0	○ 1
**18.3**	Doing moderate exercise (but not walking outside the home)? Moderate means exercise that is not exhausting. For example, biking outdoors, using an exercise machine (like a stationary bike or treadmill), calisthenics, easy swimming, popular and folk dancing.	○ 0	○ 1

○○○○○○○○○○○○○○○○○○○○○○○ 013204

At the time of your most recent fall, were you:

		No	Yes
**18.4**	Doing mild exercise? For example, slow dancing, bowling, or golf.	○0	○1
**18.5**	Doing other exercise (not previously listed)?	○0	○1
**18.6**	Doing strenuous indoor household chores (such as scrubbing floors, sweeping, or vacuuming)?	○0	○1
**18.7**	Working in the yard (such as mowing, raking, gardening, or shoveling snow)?	○0	○1

19. Were you injured as a result of any fall in the past 12 months?

○0 No

○1 Yes →

19.1 Please indicate what types of injuries. **Mark all that apply.**

○1 Fracture

○2 Laceration/cut

○3 Bruising

○4 Sprained or strained joint (wrist, knee, ankle, etc.)

○5 Other injury (Specify: ______________________________)

19.2 Did you injure your head? ○0 No ○1 Yes

These questions are about your feelings during the past week. For each of the statements, please indicate the choice that tells how often you felt that way.

		Rarely or none of the time (less than 1 day)	Some or a little of the time (1-2 days)	Occasionally or a moderate amount of time (3-4 days)	Most or all of the time (5-7 days)
**20.**	You felt depressed (blue or down)	○0	○1	○2	○3
**21.**	Your sleep was restless	○0	○1	○2	○3
**22.**	You enjoyed life	○0	○1	○2	○3
**23.**	You had crying spells	○0	○1	○2	○3
**24.**	You felt sad	○0	○1	○2	○3
**25.**	You felt that people disliked you	○0	○1	○2	○3

26. In the past year, have you had two weeks or more during which you felt sad, blue, or depressed, or lost pleasure in things that you usually cared about or enjoyed?

○0 No ○1 Yes

27. Have you had two years or more in your life when you felt depressed or sad most days, even if you felt okay sometimes?

○ 0 No ○ 1 Yes →

> **27.1.** Have you felt depressed or sad much of the time in the past year?
> ○ 0 No ○ 1 Yes

This set of questions asks about your feelings in general. Please answer the following questions honestly and quickly using the scale below:

		Not at all true of me						Very true of me
**28.**	When I hear about someone (a stranger) going through a difficult time, I feel a great deal of compassion for him or her.	○ 0	○ 1	○ 2	○ 3	○ 4	○ 5	○ 6
**29.**	I tend to feel compassion for people, even though I do not know them.	○ 0	○ 1	○ 2	○ 3	○ 4	○ 5	○ 6
**30.**	One of the activities that provide me with the most meaning to my life is helping others in the world when they need help.	○ 0	○ 1	○ 2	○ 3	○ 4	○ 5	○ 6
**31.**	I would rather engage in actions that help others, even though they are strangers, than engage in actions that would help me.	○ 0	○ 1	○ 2	○ 3	○ 4	○ 5	○ 6
**32.**	I often have tender feelings toward people (strangers) when they seem to be in need.	○ 0	○ 1	○ 2	○ 3	○ 4	○ 5	○ 6

Thank you for completing this questionnaire.
Please take a moment to review any questions you may have missed.

○○○○○○○○○○○○○○○○○○○○○○○ 013204

APPENDIX D: *WHISH*ful Actions Newsletter Summary

*WHISH*ful Actions #, NEWSLETTER TITLE, (DATE), CONTENT SUMMARY		INFORMATION INCLUDED WITH MAILING (INSERTS, SURVEYS, POSTCARDS)
**WA0**	**Getting Started** (3 waves: mailed, APR-JUL2015) Tips to Get Moving; Moving Safely; Benefits of physical activity.	**Passive Consent** letter from WHI CCC, "What is *WHISH"* letter describing the study; **"Workout to Go"** pamphlet - Go4Life®
**WA1** .	**Let Us Help You Get Moving** (JUN-SEP2015) Move a little more; Stages of change; Setting goals.	Thank you. Welcome letter from PI; **Survey #1 (2015 Preference Survey)**
**WA2** .	**Get Ready, Get Set, Go** (JUN-SEP2015) What's your starting point; Readiness checklist; Self-Test; My PA activity plan; PA knowledge crossword puzzle.	**Go4Life® Exercise and Physical Activity Guidelines** book
**WA3**	**Every Step Counts** (JUL-OCT 2015) Steps to better health; Tips for counting steps with the *WHISH* pedometer; Coming soon – the *WHISH* automated telephone support line ("ADRIANA").	***WHISH* pedometer; 2015 *WHISH* Activity calendar (SEP2015-JAN2016);** Inserts - Adriana (IVR) user guide; Stay engaged with *WHISH*
**WA4**	**All about You (Fall 2015)** Survey #1responses; photos of participants in action; Step it Up! (Surgeon General's focus on walking); Pedometer wear instructions; Three "readiness for change" scenarios.	**Consent** for *WHISH* Support Tools (Stanford); **2016 WHISH Activity Calendar**
**WA5** .	**Spring into Action (Spring 2016)** Be active in spring; Tips for moving more; Walking; Launch of *WHISH* website; how to log on to website and use PA tracking tool and resources.	***WHISH* website (**launched 2/26/16) includes activity tracker and PA resources) Insert - Update my action plan worksheet
**WA6**	**Summer is Calling (Summer 2016)** Tips to stay cool and active; "In their own words" – participants share walkers, canes & walking poles experiences; "You Tell Us" - activities participants say they enjoy; using the website activity tracker.	Insert **- "Adriana is Here,"** - how to use the Interactive Voice Response (IVR) Telephone system ("Adriana")
**WA7**	**Fall in Love with Autumn Activity (Fall 2016)** Balance & flexibility; Caregiving Challenges; ways to track activity; holidays, just around the corner	**Survey #2 (2016 – Engagement survey)** **2017 *WHISH* Activity Calendar**
**WA8**	**Happy New Year (Winter 2017)** 2017 Activity Plan goals; Testimonials: WHISH participants doing strength training; resources participants use for staying active.	Insert - Resistance band exercises ***WHISH* resistance band** physical activity aid
**WA9**	**Everyday Activities (Spring 2017)** Everyday activities made easier (participants share how they stay active); Life Happens: Resuming activity after illness or injury-participant stories	Insert -*WHISH* Challenge insert: Endurance, Upper & Lower Body Strength; Balance & Flexibility.
**WA10**	**All About You (Summer 2017)** Survey #2 Findings; *WHISH* Challenge Spotlight; Participants share activities, resources they use.	[no inserts]
**WA11**	**Balance and Fall Prevention (Fall 2017)** Exercises to prevent falls; tips for breaking up inactive sitting; participants featured using *WHISH*	**Survey #3 (2017)** **2018 *WHISH* Activity Calendar** Postcard - Update your contact info

	resistance band; Life Happens - resilience and overcoming balance setbacks.	
**WA12** .	**Striding into the New Year (Winter 2018)**<br>Implementation Intention - setting goals; Winter walking; active sitting; pedometer instructions; participants share their goals & activities.	***WHISH* pedometer;**<br>**Stick-with-it Goalsetting notepad;**<br>Insert - What motivates you? (4 motivational drivers)**;** Postcard - You Tell Us: "When you don't feel like being active, how do you get yourself to move?"; Pedometer Belt Request
**WA13**	**Light Activity Counts (Spring 2018)**<br>Key messages: Sit Less, Move More throughout the day; Start where you are; do what you can; New WHI findings on light activity (from OPACH). Life Happens: recovering from back injury.	Insert - Motivation and goal setting – participants share what motivates them to be active.
**WA14**	**Every Minute Counts (Summer 2018)**<br>Summer Challenge: Replace 30 minutes of inactive sitting with active movement; 3 steps to replace inactive time – assess inactive time, find new activities, the Talk Test; Life Happens: Moving together through tough times.	**Survey #4 (2018)**<br>Insert: -All about You: responses to Survey #3 and participant responses to WA12 postcard – "What gets you to move when you don't feel like it?"<br>Postcard: Meet *WHISH* physical therapist
**WA15**	**Physical Activity: The Best Medicine (Fall 2018)**<br>2018 Physical Activity Guidelines Scientific Report, plus physical activity recommendations from the AHA, ACS, ADA, Arthritis Foundation, and Alzheimer's Association; Life Happens: Moving for your health.	**2019 *WHISH* Activity Calendar**<br>**<u>Targeted</u> Inserts:** Interval Activities for each of Low, Middle, and High PF/PA groups<br>Inserts **–** Using the upgraded *WHISH* website; Treasure Hunt contest (website), Survey Postcard #1 (assessing your interval activity)
**WA16**	**You are Making a Difference (Winter 2019)**<br>Importance of WHI & WHISH participants in research; 2018 PA Guidelines for Americans;Website treasure hunt winners; Motivation exercise -Reflecting on your health and participation; Life Happens: Step by step.	**Targeted Inserts:** Wiser Walking (exercises to improve gait)<br>***WHISH* website videos** featuring *WHISH* participants: Learn interval activity, Warm-up and cool-down exercises; Wiser walking: Improve gait, strength and balance.
**WA17**	**Activity, Strength, and Balance (Spring 2019)**<br>2018 Physical Activity Guidelines multicomponent goals for older adults; Relative intensity of PA; Well-rounded routine/24-hour Day; News & Research: PA and brain health; Life Happens: Ways to stay strong; Ask WHISH: PA and chronic conditions (COPD).	**Targeted Inserts**: Move Your Way (multi-component activities)<br>Postcard introducing *WHISH* physical therapist & website contest to promote the new Home Exercises page and *WHISH* exercise videos;<br>***WHISH* visor** physical activity aid
**WA18**	**All About You (Summer 2019)**<br>Results from Survey #4, including participant responses about physical limitations, aches and pains, and motivation; News & Research: PA and arthritis; Life Happens; Ask *WHISH*.	**Survey #5 (2019)**<br>**Targeted Inserts:** Stretch It Out. Exercises to alleviate aches and promote flexibility.
**WA19**	**A Balancing Act (Fall 2019)**<br>Myths about fall prevention; inspiration from winners of *WHISH* website contest; check-ups to prevent falls; Exercises to maintain PF; News & Research: benefits of light activity; Ask *WHISH*: PA and chronic conditions.	**2020 *WHISH* Activity Calendar**<br>**Targeted Inserts: Balance exercises**<br>Insert: How to Use the new *WHISH* website<br>Survey Postcard #2: Join email campaign, choice of a new *WHISH* resistance band<br>New *WHISH* website video: A Balancing Act

**WA20**	**Staying Active in Winter (Winter 2020)**<br>Feel better right away; Think differently about "Exercise"; Staying active in winter; A well-rounded routine; Ask a *WHISH* participant: testimonials.	**Targeted Inserts**: Multicomponent exercises and exercises using resistance bands.<br>**New *WHISH* resistance bands** included (sent only to participants who requested them)
**WA21**	**Preserve Your Reserve (Spring 2020)**<br>Functional capacity and self-assessment of functional fitness.	**Targeted Inserts:** Maintain your mobility: Home exercises to improve functional capacity while sheltering in place and social distancing.<br>Insert letter - CDC guideline for PA during COVID.<br>***WHISH* COVID-19 Survey (email)** sent MAY2020
**WA22**	**All About You (Summer 2020)**<br>Findings from the 2019 Annual *WHISH* Survey #5 Using activities you enjoy to maintain PA; participant testimonials about how PA enhances their lives.	Inserts- Responses to the COVID-19 survey; online resources during COVID; *WHISH* Stay active challenge (multicomponent activities); Walk across America challenge (cumulative step count of all contestants).
**WA23**	**Take Steps to Prevent Falls (Fall 2020)**<br>Steps to prevent falls; Modify your home to stay active; My *WHISH* Story- participant stories about resuming activity after a fall or modifying their home to stay active with a disability.	**2021 Activity Calendar** (includes functional fitness self-assessment)<br>**Targeted Inserts:** Fall Prevention (gentle yoga exercises for strength, balance and flexibility)<br>***WHISH* Bandana** physical activity aid
**WA24**<br>.	**Moving Forward (Winter 2021)**<br>*WHISH* Continuation Letter; Benefits of standing and light activity- all movement counts; Stay Active Challenge results and winners.	**Survey # 6 (2021)**<br>**Targeted Inserts:** Tune-up your joints – moving with music; exercises to loosen joints and build endurance and strength.
**WA25**	**A New Day (Spring/Summer 2021)**<br>The 24-hour day – break up inactive sitting with light, moderate and vigorous activity; the Talk Test.	Insert – Your 24-hour day worksheet<br>Postcard - Request *WHISH* materials and PA aids, intentional exercise vs. routine activity.<br>**New WHISH pedometer** (only by request)
**WA 26**	**All Activity Counts (Fall-Winter 2021)**<br>All movement counts toward maintaining physical function and health. It all adds up – move more and sit less; News & Research What Moves You? Research on motivation and findings from 2019 *WHISH* postcard.	**2022 *WHISH* Activity Calendar**<br>**Targeted Inserts:** 2021 Fall Prevention-exercises<br>Inserts- Home Inventory, Neighborhood Destinations<br>**Mindset survey** (email – MAR2022)
**WA27**<br>.	**All About You (Spring 2022)**<br>Responses from Survey #6; What participants want to tell other women their age about being active.	**Targeted Inserts:** Moving forward – what type of activity do participants want to improve most - Balance. Multicomponent exercises to improve balance, strength, endurance.
**WA28**	**Active For Life (Early Fall 2022)**<br>Celebrating three *WHISH* participants who have reached the age of 100 years: their thoughts about what helps them stay active; News and Research: Moving after meals for high blood sugar, Better balance for longer life; The value of	**Targeted Inserts:** Physical Activity Mindset substudy (mailed materials). Participants randomized to either Intervention insert (Make A New Goal With Exercise: To Enjoy It) or Control insert (Get Healthy Through Movement).

	intermittent bouts of light physical activity; Participant suggestions for moving more and sitting less.	
**WA29**	**Find the Right Balance (December 2022)**<br>What is balance; Balance confidence; Improve balance to reduce risk of falling; How to get started; How to get up from a fall; Introduction to Corey Rovzar, new *WHISH* physical therapist. Theme of WA29-WA33 –<br><br>Balance + Strength +Endurance + Flexibility= Mobility.	**Survey #7 2022**<br>**2023 *WHISH* Activity Calendar** includes Fall Prevention- Home Safety Checklist, How to get up from a fall, functional fitness assessment<br>**Targeted Inserts**: Find the right balance - **FOUR insert groups** – added Very Low group to Lower, Middle, and Higher. Balance exercises and use of walking sticks/ walkers to improve and preserve mobility.
**WA30**	**Strength and Stability (Spring 2023)**<br>Role of strength in improving balance; What does it mean to build strength; Strength building routine – how much and how often; ASK *WHISH*: participant asks about neuropathy	**Targeted inserts:** Strength and stability - lower body strength and stability exercises; daily strength and balance record; monthly progress test for strength.<br>***WHISH* Fanny pack** physical activity aid
**WA31**	**Endurance and Mobility (Summer 2023)**<br>Building endurance and stamina; Endurance activities; All About You: responses to the *WHISH* 2022 Survey, News & Research: The power of mindset; *WHISH* participant testimonial: My daily routine.	Letter to participants: request more or less challenging targeted inserts with next newsletter.<br>Inserts - All participants received both intervention and control Mindset materials.
**WA32**	**Flexibility and Fall Prevention (Late Fall 2023)**<br>What is Flexibility; Flexibility and arthritis; Stretching strategies; Walking to reduce joint pain and stiffness; *WHISH* participant suggestions for turning inactive minutes into active minutes.	**2024 *WHISH* Activity Calendar,**<br>**Targeted Inserts:** Flexibility and fall prevention - multicomponent strength and flexibility exercises
**WA33**<br>.	**Step Right Up (April 2024)**<br>You can improve your mobility; Step this way: The best way to walk and challenge your balance; *WHISH* participant testimonial: "I want to stay active as long as I can"; News & Research: Preservation of PA in older adults; the Gradual Progression of Function Model; Introducing the Boost Your Balance program.	Letter from Dr. Stefanick: the *WHISH* trial will be ending soon but WHI will continue.<br>**Targeted Inserts:** Step Right Up - exercises to improve strength and mobility.
**WA34**	**The Values Connection (July 2024)**<br>Let your values move you; How your values can motivate you to be active; Catch It. Check It. Change It: how our thoughts can hold us back from making changes; The basics components of physical activity; Participant testimonial – Lessons learned from WHISH: you don't have to run marathons.	**Survey #8 2024**<br>Insert - The Values connection –Values Card Sort exercise to determine your five most important personal values and how to connect those values to being physcally active and strong.

**WA35**	**Do What You Can (October 2024)**<br>Staying active with chronic conditions; recommendations from national organizations for managing chronic conditions with physical activity; News & Research: Resistance training for better health; Think differently about motivation: multiple strategies to promote successful behavior changes; Participant testimonial: successful aging and physical activity.	Insert – Effective communication for managing your health: Talking with friends and family; Talking with your health care team.
**WA36**	**Final Newsletter (February 2025)**<br>Key components for staying active; News & Research: What is the *WHISH* trial and why is it important; Frequently asked questions; Contact information, resources for *WHISH* participants.	**2025 WHISH Activity Calendar** (including blank calendar months to copy and use beyond 2025).
**WHISH Toolkit: (December 2025)** Greatest hits from *WHISH* materials: The basics of staying active; Essential exercises to maintain mobility; Motivational strategies; Tip sheets; WHISH website; Resources.		

Section/topic	No	CONSORT 2025 checklist item description	Reported on page no.
**Title and abstract**			
Title and structured abstract	1a	Identification as a randomised trial	1
	1b	Structured summary of the trial design, methods, results, and conclusions	3
**Open science**			
Trial registration	2	Name of trial registry, identifying number (with URL) and date of registration	24
Protocol and statistical analysis plan	3	Where the trial protocol and statistical analysis plan can be accessed	Appendix
Data sharing	4	Where and how the individual de-identified participant data (including data dictionary), statistical code and any other materials can be accessed	24
Funding and conflicts of interest	5a	Sources of funding and other support (eg, supply of drugs), and role of funders in the design, conduct, analysis and reporting of the trial	22, 24
	5b	Financial and other conflicts of interest of the manuscript authors	24
**Introduction**			
Background and rationale	6	Scientific background and rationale	5-6
Objectives	7	Specific objectives related to benefits and harms	6
**Methods**			
Patient and public involvement	8	Details of patient or public involvement in the design, conduct and reporting of the trial	NA
Trial design	9	Description of trial design including type of trial (eg, parallel group, crossover), allocation ratio, and framework (eg, superiority, equivalence, non-inferiority, exploratory)	7
Changes to trial protocol	10	Important changes to the trial after it commenced including any outcomes or analyses that were not prespecified, with reason	NA
Trial setting	11	Settings (eg, community, hospital) and locations (eg, countries, sites) where the trial was conducted	7
Eligibility criteria	12a	Eligibility criteria for participants	7
	12b	If applicable, eligibility criteria for sites and for individuals delivering the interventions (eg, surgeons, physiotherapists)	NA
Intervention and comparator	13	Intervention and comparator with sufficient details to allow replication. If relevant, where additional materials describing the intervention and comparator (eg, intervention manual) can be accessed	7-8
Outcomes	14	Prespecified primary and secondary outcomes, including the specific measurement variable (eg, systolic blood pressure), analysis metric (eg, change from baseline, final value, time to event), method of aggregation (eg, median, proportion), and time point for each outcome	Appendix, Protocol
Harms	15	How harms were defined and assessed (eg, systematically, non-systematically)	Appendix, Protocol, Safety Outcomes
Sample size	16a	How sample size was determined, including all assumptions supporting the sample size calculation	Appendix, Protocol, Statistics

	16b	Explanation of any interim analyses and stopping guidelines	Appendix, Protocol, Statistics
Randomisation:			
Sequence generation	17a	Who generated the random allocation sequence and the method used	Appendix
	17b	Type of randomisation and details of any restriction (eg, stratification, blocking and block size)	Appendix
			**Reported on page no.**
Allocation concealment mechanism	18	Mechanism used to implement the random allocation sequence (eg, central computer/telephone; sequentially numbered, opaque, sealed containers), describing any steps to conceal the sequence until interventions were assigned	Appendix
Implementation	19	Whether the personnel who enrolled and those who assigned participants to the interventions had access to the random allocation sequence	Appendix
Blinding	20a	Who was blinded after assignment to interventions (eg, participants, care providers, outcome assessors, data analysts)	9, Appendix
	20b	If blinded, how blinding was achieved and description of the similarity of interventions	9, Appendix
Statistical methods	21a	Statistical methods used to compare groups for primary and secondary outcomes, including harms	12-13, Appendix
	21b	Definition of who is included in each analysis (eg, all randomised participants), and in which group	12-13, Appendix
	21c	How missing data were handled in the analysis	NA
	21d	Methods for any additional analyses (eg, subgroup and sensitivity analyses), distinguishing prespecified from post hoc	12,13, Appendix
**Results**			
Participant flow, including flow diagram	22a	For each group, the numbers of participants who were randomly assigned, received intended intervention, and were analysed for the primary outcome	Figure 1
	22b	For each group, losses and exclusions after randomisation, together with reasons	Figure 1
Recruitment	23a	Dates defining the periods of recruitment and follow-up for outcomes of benefits and harms	7, Appendix, Protocol
	23b	If relevant, why the trial ended or was stopped	NA
Intervention and comparator delivery	24a	Intervention and comparator as they were actually administered (eg, where appropriate, who delivered the intervention/comparator, how participants adhered, whether they were delivered as intended (fidelity))	Appendix
	24b	Concomitant care received during the trial for each group	NA
Baseline data	25	A table showing baseline demographic and clinical characteristics for each group	Table 1
Numbers analysed, outcomes and estimation	26	For each primary and secondary outcome, by group:	
		● the number of participants included in the analysis	Table 2
		● the number of participants with available data at the outcome time point	Table 2
		● result for each group, and the estimated effect size and its precision (such as 95% confidence interval)	Table 2
		● for binary outcomes, presentation of both absolute and relative effect size	NA
Harms	27	All harms or unintended events in each group	NA
Ancillary analyses	28	Any other analyses performed, including subgroup and sensitivity analyses, distinguishing pre-specified from post hoc	NA
**Discussion**			

Interpretation	29	Interpretation consistent with results, balancing benefits and harms, and considering other relevant evidence	16-21
Limitations	30	Trial limitations, addressing sources of potential bias, imprecision, generalisability, and, if relevant, multiplicity of analyses	20 (limitations), 19 (generalisability)

Citation: Hopewell S, Chan AW, Collins GS, Hróbjartsson A, Moher D, Schulz KF, et al. CONSORT 2025 Statement: updated guideline for reporting randomised trials. BMJ. 2025; 388:e081123.
https://dx.doi.org/10.1136/bmj-2024-081123
© 2025 Hopewell et al. This is an Open Access article distributed under the terms of the Creative Commons Attribution License (https://creativecommons.org/licenses/by/4.0/), which permits unrestricted use, distribution, and reproduction in any medium, provided the original work is properly cited.

*We strongly recommend reading this statement in conjunction with the CONSORT 2025 Explanation and Elaboration and/or the CONSORT 2025 Expanded Checklist for important clarifications on all the items. We also recommend reading relevant CONSORT extensions. See www.consort-spirit.org.
